# Analysis of the Healthy Platelet Proteome Identifies a New Form of Domain-Specific *O-*Fucosylation

**DOI:** 10.1016/j.mcpro.2024.100717

**Published:** 2024-01-16

**Authors:** Callum B. Houlahan, Yvonne Kong, Bede Johnston, Michelle Cielesh, The Huong Chau, Jemma Fenwick, Paul R. Coleman, Huilin Hao, Robert S. Haltiwanger, Morten Thaysen-Andersen, Freda H. Passam, Mark Larance

**Affiliations:** 1 The Heart Research Institute, Charles Perkins Centre, The University of Sydney, Sydney, New South Wales, Australia; 2 Central Clinical School, The University of Sydney, Sydney, New South Wales, Australia; 3 Charles Perkins Centre, School of Medical Sciences, The University of Sydney, Sydney, New South Wales, Australia; 4 School of Natural Sciences, Macquarie University, Macquarie Park, New South Wales, Australia; 5 Department of Biochemistry and Molecular Biology, Complex Carbohydrate Research Center, University of Georgia, Athens, Georgia, USA; 6 Institute for Glyco-Core Research, Nagoya University, Nagoya, Aichi, Japan

**Keywords:** platelet, secretome, fucose, EMI domain, glycosylation, secretion, human

## Abstract

Platelet activation induces the secretion of proteins that promote platelet aggregation and inflammation. However, detailed analysis of the released platelet proteome is hampered by platelets’ tendency to preactivate during their isolation and a lack of sensitive protocols for low abundance releasate analysis. Here, we detail the most sensitive analysis to date of the platelet releasate proteome with the detection of >1300 proteins. Unbiased scanning for posttranslational modifications within releasate proteins highlighted *O-*glycosylation as being a major component. For the first time, we detected *O-*fucosylation on previously uncharacterized sites including multimerin-1 (MMRN1), a major alpha granule protein that supports platelet adhesion to collagen and is a carrier for platelet factor V. The N-terminal elastin microfibril interface (EMI) domain of MMRN1, a key site for protein–protein interaction, was *O-*fucosylated at a conserved threonine within a new domain context. Our data suggest that either protein *O-*fucosyltransferase 1, or a novel protein *O-*fucosyltransferase, may be responsible for this modification. Mutating this *O-*fucose site on the EMI domain led to a >50% reduction of MMRN1 secretion, supporting a key role of EMI *O-*fucosylation in MMRN1 secretion. By comparing releasates from resting and thrombin-treated platelets, 202 proteins were found to be significantly released after high-dose thrombin stimulation. Complementary quantification of the platelet lysates identified >3800 proteins, which confirmed the platelet origin of releasate proteins by anticorrelation analysis. Low-dose thrombin treatment yielded a smaller subset of significantly regulated proteins with fewer secretory pathway enzymes. The extensive platelet proteome resource provided here (larancelab.com/platelet-proteome) allows identification of novel regulatory mechanisms for drug targeting to address platelet dysfunction and thrombosis.

## Highlights

• Human platelet lysate and releasate proteomes were characterized in detail.
• >200 proteins were significantly released after high-dose thrombin stimulation.
• *O*-Glycosylation was detected as a dominant modification of the secreted proteins.
• A new form of domain-specific *O*-fucosylation was detected on the protein MMRN1.

## Graphical Abstract

**Figure:**
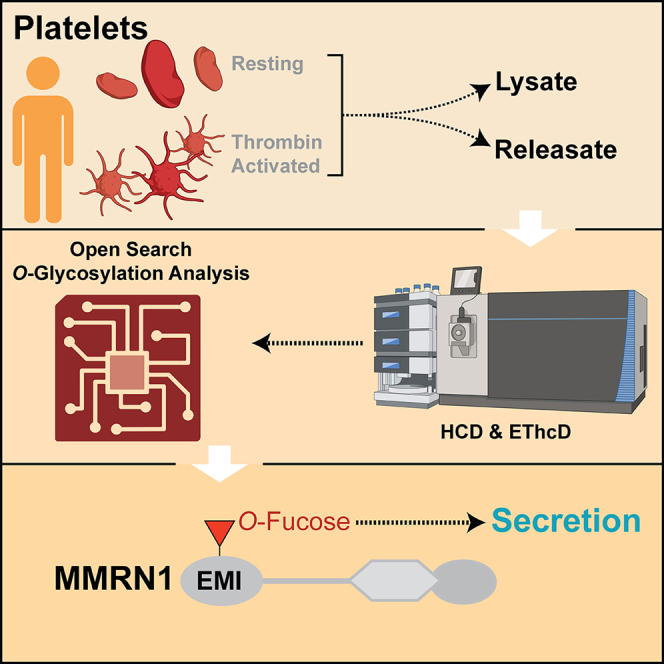

## In Brief

The human platelet lysate and releasate proteome was examined using an ultrasensitive protocol. By comparing releasates from resting and thrombin-treated platelets, 202 proteins were found to be significantly released after high-dose thrombin stimulation. Unbiased scanning for posttranslational modifications within releasate proteins highlighted *O*-glycosylation as being a major component. Elastin microfibril interface domain-specific *O*-fucosylation was detected on the protein MMRN1 and was demonstrated to be important for its secretion.

Platelets are circulating cells that activate and aggregate after contact with damaged vascular endothelium to promote thrombus formation during hemostasis. It is also well established that platelets play a role in inflammation and vascular repair (1). Activation, by both chemical and mechanical stimuli, leads to the release of granule contents including soluble proteins, cleaved membrane proteins, and vesicle-bound proteins (2), which subsequently activates a variety of signaling pathways. Released proteins (*i.e.*, the “releasate”) include those synthesized within the parent megakaryocyte cytoplasm, proteins endocytosed from plasma, as well as proteins synthesized from platelet mRNA (3, 4).

Thrombin activates platelets *via* the protease-activated receptors PAR1 and PAR4 (F2R and F2RL3) (5, 6, 7, 8). PAR1 mediates platelet activation at low thrombin concentrations (<0.05 U/ml), whereas PAR4 requires a comparatively higher thrombin concentration (>0.1 U/ml) to activate platelets (9, 10). Differential release of proangiogenic and antiangiogenic factors has been shown with selective stimulation of either PAR1 or PAR4 (6). However, Holten *et al.* (11) identified no qualitative differences upon activation with specific PAR1 and PAR4 agonists. After activation, platelets can release the contents of α-granules, dense granules, tertiary granules, and lysosomes (12). Platelet α-granules contain adhesive proteins such as fibrinogen, von Willebrand factor, multimerin-1 (MMRN1), and fibronectin, which enable effective thrombus formation. Growth factors (*e.g.*, platelet-derived growth factor subunit A, vascular endothelial growth factor C) and chemokines (*e.g.*, CCL5, CXCL3) are also released to regulate the immune response and tissue repair. Platelet lysosomes contain proteases, glycosidases, and acid hydrolases that have bactericidal activity (2) and may also play a role in receptor cleavage and fibrinolysis (13, 14).

Unbiased mass spectrometry (MS)-based proteomics can provide unique insights into cellular protein structure, modifications, and function. Many groups have performed platelet proteomic analysis to understand their protein composition and regulation (2, 15, 16). While most proteomic studies have been performed on total platelet lysates or subcellular compartments (17), the analysis of platelet releasate is much more challenging due to the low concentration of secreted factors. In 2004, Coppinger *et al.* (18) was the first to characterize human platelet releasates after thrombin activation (0.5 U/ml) reporting >300 different proteins using 2D-PAGE. With a novel protein labeling approach a subsequent study identified 124 releasate proteins, following high-dose thrombin (1 U/ml) and collagen (5 μg/ml) stimulation (19). Using different methods Parsons *et al.* (20) detected 894 released proteins after a similar high-dose thrombin stimulus (1 U/ml). The differences in these releasate proteomes primarily reflect variance in platelet isolation, platelet stimulation and protein analysis methods. An overlooked aspect of these released proteins is their decoration with posttranslational modifications (PTMs), including *O-*glycosylation, N-glycosylation, and proteolytic cleavage, which are important for protein function (21).

Important questions remain as to the regulation and composition of proteins in healthy human platelets. For example, no study has provided an unbiased view of the PTMs present on platelet releasate proteins. Here, we have addressed these questions using a robust platelet isolation method, coupled to the latest quantitative proteomics and glycomics methodologies for the best sensitivity and accuracy. Subsequent unbiased PTM analysis revealed a wealth of detail in the releasate proteome and highlighted *O-*glycosylation as a common modification with a large number of unique modification sites identified for the first time. The discovery of *O-*fucosylation of MMRN1 at T216 within its elastin microfibril interface (EMI) domain provides a novel putative substrate for either POFUT1 or a novel platelet POFUT. This ultrasensitive human platelet proteome is shared with the community (larancelab.com/platelet-proteome) to enable future studies in human platelet biology.

## Experimental Procedures

### Patient Blood Collection

Human ethics was from the University of Sydney (approval number 2014/244) and our study abides by the Declaration of Helsinki principles. Venepuncture was performed using a 19-gauge needle and light tourniquet. Blood was collected using 16 × 100 mm 8.5 ml vacutainer glass whole blood acid-citrate-dextrose (ACD) tubes (Becton Dickinson, Cat# 366645) and gently mixed following collection. Blood was collected from healthy volunteers free from medication for the past 10 days ranging from 22 to 60 years old (median age 32 years old).

### Platelet Isolation From Whole Blood

Platelets were isolated from whole blood within 3 to 4 h postvenepuncture. Whole blood was fractioned by centrifugation (200*g* for 20 min, brake = 0) to separate platelet-rich plasma from red blood cell (RBC) and white blood cell (WBC) fractions. All centrifugation steps were performed at room temperature. Platelet-rich plasma was rested for 30 min in a water bath at 37 °C before the addition of 20% (v/v) prewarmed (37 °C) ACD (Cat# C3821, Sigma-Aldrich). Platelets were separated from plasma by centrifugation (800*g* for 10 min, brake = 4). The platelets were resuspended in prewarmed (37 °C) modified Hepes/Tyrodes (HTGlc) buffer (129 mM NaCl, 0.34 mM Na_2_HPO_4_, 2.9 mM KCl, 12 mM NaHCO_3_, 20 mM Hepes, 5 mM glucose, 1 mM MgCl_2_; pH 7.4). Resuspended platelets were rested for 20 min in a 37 °C water bath, following the addition of 10% (v/v) prewarmed (37 °C) ACD and 0.02 U/ml apyrase. Platelets were pelleted by centrifugation (800*g* for 5 min, brake = 4) before resuspension in prewarmed HTGlc buffer at the working concentration of 400 × 10^3^/μl. Addition of prostaglandin E1 (Cat# P5515, Sigma-Aldrich) (2 μM) took place immediately before all centrifugation steps to minimize platelet activation. Platelet concentration was maintained below 10^6^/μl during washing stages. Platelet concentration was measured using a Sysmex KX-21N haemocytometer.

### Separation of Platelet Releasate and Lysate for Proteomics

Washed platelets (400 × 10^3^/μl) were divided into 250 μl aliquots. To a resting control, 0.02 U/ml apyrase was added, while other aliquots were activated with thrombin (T6884, Sigma-Aldrich) at a final activity of 0.2 U/ml for maximal stimulation or 0.025 U/ml for submaximal stimulation. All samples were incubated in a 37 °C water bath for 5 min. Following incubation, D-phenylalanyl-N-[(1S)-4-[(aminoiminomethyl)amino]-1-(2-chloroacetyl)butyl]-L-prolinamide dihydrochloride (Cat# ab141451, Abcam) was added at 25 nM to the thrombin-stimulated sample and 2 μM prostaglandin E1 was added immediately prior to centrifugation of the resting sample. The supernatant at this point was regarded as the “platelet releasate” and was aspirated and stored under argon at −80 °C. The remaining platelet protein was regarded as the “platelet lysate.” Pellet lysate was obtained *via* resuspension in sodium deoxycholate (SDC) lysis buffer (4% (w/v) sodium deoxycholate in 0.1 M Tris–HCl (pH 8) and heating at 95 °C for 10 min. Protein concentration was determined by bicinchoninic acid assay (Cat# 23227, Thermo Fisher Scientific). Platelet lysates were aliquoted and stored under argon at −80 °C.

### Flow Cytometry Analysis of Washed Platelet Contamination and Platelet Preactivation

The expression of key platelet membrane-specific proteins α_IIb_β_3_ and P-selectin (CD62p) was used to assess platelet preactivation prior to thrombin stimulation. Platelet preactivation was measured, following platelet releasate and lysate storage and within 5 h postvenepuncture. Washed platelets were suspended in HTGlc buffer at a concentration of 10 × 10^3^/μl and stained with either mouse antihuman α_IIb_β_3_ (PAC-1) antibody conjugated with FITC (BD Biosciences, Cat# 340507) or mouse antihuman CD62p antibody conjugated with allophycocyanin (BD Biosciences, Cat# 550888). PAC-1 detects the active confirmation of αIIbβ3. Platelet activation was achieved *via* the addition of thrombin (0.025 or 0.2 U/ml) to washed platelet preparation for 10 min at room temperature and was cancelled *via* addition of 25 μM D-phenylalanyl-N-[(1S)-4-[(aminoiminomethyl)amino]-1-(2-chloroacetyl)butyl]-L-prolinamide dihydrochloride. Washed unstained, stained resting, and stained thrombin-activated platelets were analyzed by flow cytometry using a Becton Dickinson Accuri C6 flow cytometer to confirm expression of α_IIb_β_3_ and CD62p. Data analysis was achieved using FlowJo software (FlowJo, LLC, https://www.flowjo.com/).

For analysis of WBC and RBC contamination in washed platelet preparations, samples were stained with antihuman CD45 IgG conjugated to PerCP-Cy5.5 (Becton Dickinson Biosciences, Cat# 340953,) and mouse anti-human CD235a (Glycophorin A) IgG conjugated to FITC (Cat# 349103, BioLegend), respectively. Both samples were additionally stained with mouse antihuman CD41 IgG conjugated with FITC (Cat# IMO649U, Beckman Coulter). Unstained washed platelets were used as the control and cell fluorescence was recorded by flow cytometry using a Accuri C6 flow cytometer. CD41+ cells were gated as platelets (supplemental Fig. S1). Since platelets are significantly smaller in size than RBCs and WBCs and have significantly less granularity than WBCs, events beyond the upper limits of platelet side scatter (granularity) and forward scatter (size) were regarded as either WBCs or RBCs (supplemental Fig. S1). Events above these upper limits which were CD45+ were regarded as WBCs, and CD235a+ events were regarded as RBCs. Event counts of each subpopulation were used to derive a ratio of platelets to WBCs and platelets to RBCs at a higher sensitivity than which was achievable using a cell counter.

### Determination of Flow Cytometry Cut-Offs for Preactivation and Thrombin Doses

The laboratory has an established routine platelet isolation protocol by sequential centrifugation steps as described previously (22) and detailed below. Using this method, we have determined the % staining for mouse anti-human CD62p allophycocyanin and antihuman αIIbβ3 IgG (PAC-1) FITC in the resting platelet population compared with unstained resting platelets (n = 30). The average % + 2 SD of this historical group was used as a cut-off for inclusion of resting platelet preparations from healthy donors included in this study. Samples that exhibited CD62PP >25% and PAC-1 >15% were excluded from further analysis. These percentages for CD62P and PAC-1 are in line with a previous study of platelet isolation for proteomic analysis (23).

For establishing sub-maximal (“low”) and maximal (“high”) thrombin dose, platelets were isolated from five healthy donors. Platelet aggregation was recorded over 10 min to doses of thrombin: 0.02, 0.03, 0.05, and 0.2 U/ml. Light transmission aggregometry was evaluated as described using an AggRAM 1484 (24). Experiments were conducted in 300 μl aliquots of washed platelets (400 × 10^3^/μl) buffered in HTGlc. The washed platelet suspension was mixed by magnetic stirrer. After the addition of thrombin, platelet aggregation was recorded for 10 min. The maximum aggregation was determined as the peak light transmission. Based on the aggregation response for this group of individuals and the batch of thrombin used in this study, we determined submaximal (low) dose of thrombin as 0.025 U/ml and maximal (high) dose of thrombin as 0.2 U/ml.

### Protein Sample Preparation for Mass Spectrometry–Based Proteomics

Proteins (5 μg for lysates, 1 μg for releasates, and 70 μg for plasma) were denatured, reduced, and alkylated by resuspension in 4% (w/v) SDC, 10 mM tris-2-carboxyethyl-phosphine, 40 mM chloroacetamide, and 100 mM Tris–HCl (pH 8), followed by heating to 95 °C for 10 min. Samples were then diluted to a final concentration of 1% (w/v) SDC using water and digested for 16 h with trypsin 1:50 (w/w) (Sigma Cat# T6567) at 37 °C at 1000 rpm in a Thermomixer-C (Eppendorf). Samples were mixed 1:1 (v:v) with 99% ethyl acetate in 1% (both v/v) trifluoroacetic acid and vortexed until all the precipitated SDC was resuspended. StageTips purification of peptides was performed as described (25). Peptides were reconstituted with 5% (v/v) formic acid in water at ∼0.2 μg/μl and stored at 4 °C until LC-MS/MS analysis.

### Proteome Analysis with LC-MS/MS and Data Analysis

Peptide samples (0.5 μg) were injected onto a 50 cm × 75 μm C18 (Dr Maisch, 1.9 μm) fused silica analytical column with a 10 μm pulled tip, coupled online to a nanospray electrospray ionization (ESI) source. Peptides were resolved over a gradient from 5% to 35% acetonitrile (ACN) over 70 min with a flow rate of 300 nl/min. Peptides were ionised by ESI at 2.4 kV. Tandem mass spectrometry (MS/MS) analysis was performed using a Fusion Lumos tribrid mass spectrometer (Thermo Fisher Scientific) with either higher energy collisional dissociation (normalized collision energy [NCE] = 30), or electron-transfer higher energy collisional dissociation (EThcD, charge state filtering z = 3–8, calibrated charge-dependent electron transfer dissociation parameters and higher-energy collisional dissociation (HCD) NCE = 15). MS/MS spectra were attained in a data-dependent acquisition of the top 20 most abundant ions in each MS1 full scan. RAW data files were analyzed using the integrated quantitative proteomics software and search engine MaxQuant (26) (version 1.6.3.4, https://maxquant.org/). A false discovery rate (FDR) of 1% using a target-decoy–based strategy was used for protein and peptide identification. The database used for identification contained the UniProt human database (downloaded fifth of May 2020) alongside the MaxQuant contaminants database. Mass tolerance was set to 4.5 ppm for precursor ions and 20 ppm for fragments. Trypsin was set as the digestion enzyme with a maximum of two missed cleavages. Oxidation of Met, deamidation of Asn/Gln, pyro-Glu/Gln, and protein N-terminal acetylation were set as variable modifications. Carbamidomethylation of Cys was set as a fixed modification. The Max label-free quantitation (LFQ) algorithm was used for LFQ (27).

### Unbiased (Open) PTM Search

Data from all high thrombin-treated platelet releasates was combined with Proteome Discoverer 2.5 (Thermo Fisher Scientific, https://www.thermofisher.com/) into an mzML file and searched using Byonic (Protein Metrics v3.11.3) (28). Initially the search was performed against the whole human proteome database without PTMs. The identified 1529 proteins were converted into a focused database (supplemental File 1) used for untargeted PTM detection using “wildcard” (open) searches PTMs (29). An FDR of 2% using a target-decoy–based strategy was used for protein and peptide identification. MS1 and MS2 mass tolerance was set to 4 ppm and 20 ppm, respectively. Trypsin was set as the digestion enzyme with a maximum of two missed cleavages. Oxidation of Met (common2), deamidation of Asn/Gln (common1), pyro-Glu/Gln(rare1), and protein N-terminal acetylation (rare1) were set as variable modifications. Carbamidomethylation of Cys was set as a fixed modification. Total common max and total rare max were both set to 1. The wildcard search was applied to “unmodified” peptides with a range from −40 to 1000 Da on any amino acid. Acceptance criteria for plotting of the glycopeptide peptide spectral matches was Log probability >8 and to be modified on any of the following amino acids: S,T,Y,K,R,D,E,N,Q,P,M, and W.

### *O*-Glycan Focused PTM Search

To look for fragment ions specific for *O*-glycan cleavage data from all high thrombin-treated platelet releasates was combined and searched using Byonic (v3.11.3) (28). The platelet releasate focused database (supplemental File 1) containing 1529 proteins was used. An FDR of 2% using a target-decoy–based strategy was used for protein and peptide identification. MS1 and MS2 mass tolerance was set to 4 ppm and 20 ppm, respectively. Trypsin was set as the digestion enzyme with a maximum of two missed cleavages. Oxidation of Met (common2), deamidation of Asn/Gln (common1), pyro-Glu/Gln(rare1), protein N-terminal acetylation (rare1), hydroxylation of Asn, and tryptophan C-mannosylation (rare3) were set as variable modifications. Additional variable modifications all set to rare1 for *O*-glycosylation (as observed in the open-search and/or as Byonic mammalian *O*-glycan defaults) were included: Fuc(1), HexNAc(1)Fuc(1), Hex(1)Fuc(1), HexNAc(1)Hex(1)Fuc(1), HexNAc(1)Hex(1)Fuc(1)NeuAc(1), HexNAc(1)Hex(1)NeuAc(2), Hex(2)Fuc(1), Hex(3), Hex(1), HexNAc(1), Hex(1)Pent(2), Hex(1)+15.9949, HexNAc(1)Hex(1)NeuAc(1), HexNAc(2)Hex(2)NeuAc(2), Hex(1)Pent(3), HexNAc(2)Hex(2)NeuAc(1), HexNAc(1)Hex(1)NeuAc(3), Hex(1)NeuAc(1), HexNAc(2)Hex(2)Fuc(1)NeuAc(1). Carbamidomethylation of Cys was set as a fixed modification. Total common max was set to 1 and total rare max was set to 2. Acceptance criteria for all glycopeptides was Log probability >3. In addition, glycopeptide peptide spectral matches from HCD spectra were all assigned as having ambiguous glycosylation site localisation due to the difficulty in their interpretation. EThcD spectra were labeled as having unambiguous glycosylation site localization only after manual validation of MS/MS spectra. It should be noted that Byonic does not indicate if insufficient information is available for glycosylation site localization.

### SDS-PAGE and Coomassie Staining

Protein samples were reduced and denatured in SDS and beta-mercaptoethanol at 95 °C for 10 min before loading onto precast 4 to 20% polyacrylamide gels (Cat# 4561094, Bio-Rad Laboratories). Electrophoresis was performed for 1.5 h at a voltage of 100 V alongside Novex prestained protein standards (Cat# LC5800, Invitrogen). Following electrophoresis, gels stained with Coomassie Brilliant Blue R-250 Staining Solution (Cat# 161-0436, Bio-Rad Laboratories) for 1 h with gentle agitation. Gels were imaged using a near IR fluorescence scanner (Odyssey CLx Imaging System, Li-Cor).

### *O-*Glycome Sample Preparation

Quantitative *O-*glycomics analysis of the platelet releasate fractions and bovine fetuin (sample handling and LC-MS/MS control, Sigma-Aldrich) were performed using an established porous graphitized carbon (PGC)-LC-MS/MS as previously described (30, 31). Briefly, 20 μg total protein from each platelet releasate sample (and from bovine fetuin) was reduced with 10 mM aqueous DTT for 45 min at 56 °C and carbamidomethylated with 25 mM aqueous iodoacetamide for 30 min in the dark at 20 °C. The alkylation reaction was quenched with 30 mM aqueous DTT (final concentrations stated). The proteins were spotted onto an activated 0.45 μm polyvinylidene difluoride membrane (MerckMillipore), dried, stained with Direct Blue, and excised. The excised spots were transferred to separate wells in a flat-bottomed polypropylene 96-well plate (Corning Life Sciences), blocked with 1% (w/v) polyvinylpyrrolidone in 50% (v/v) aqueous methanol, and washed with MilliQ water. The *N*-glycans were exhaustively released using 2 U recombinant *Elizabethkingia miricola* peptide:*N*-glycosidase F expressed in *Escherichia coli* (Promega) per 20 μg protein in 10 μl water per well and incubated for 16 h at 37 °C. A second round of peptide:*N*-glycosidase F–based *N*-glycan release was performed the next day to ensure complete removal of all *N*-glycans from the protein samples to avoid cross contamination of *N*-glycans in the subsequent *O-*glycan samples. The *O-*glycans were subsequently released by incubation with 20 μl 0.5 M sodium borohydride in 50 mM aqueous potassium hydroxide for 16 h at 50 °C. The reduction reaction was then quenched using 2 μl glacial acetic acid and the released and reduced *O-*glycans were transferred into fresh 1.5 ml Eppendorf tubes. Dual desalting of the reduced *O-*glycans was performed using firstly strong cation exchange resin (AG 50W-X8 Resin, Bio-Rad) (where the *O-*glycans were not retained), followed by PGC resin (where *O-*glycans were retained) custom packed as microcolumns on top of C18 discs (Merck Millipore) in P10 solid-phase extraction formats. Following microcolumn equilibration and sample loading and washing, the *O-*glycans were eluted from the PGC–solid-phase extraction microcolumns using 0.05% trifluoroacetic acid/40% ACN/59.95% water (all v/v), dried and resuspended in 20 μl water. Samples were centrifuged at 14,000*g* for 10 min at 4 °C and the clear supernatant fractions were carefully transferred to high recovery glass vials (Waters) to avoid debris and particulates in the LC-MS/MS injection vials.

### *O-*Glycan Profiling With PGC-LC-MS/MS

The *O-*glycans were profiled using a well-established PGC-LC-MS/MS method (30, 31). In brief, the *O-*glycan samples were injected on a HyperCarb KAPPA PGC-LC column (particle/pore size, 3 μm/250 Å; column length, 30 mm; inner diameter, 0.181 mm, Thermo Hypersil) heated to 50 °C. The *O-*glycans were separated over a 60 min linear gradient of 0 to 45% (v/v) pure ACN (solvent B) in 10 mM aqueous ammonium bicarbonate (solvent A) on a 1260 Infinity Capillary HPLC system (Agilent) operating with a constant flow rate of 20 μl/min. The separated *O-*glycans were introduced directly into the mass spectrometer, ionized using ESI and detected in negative-ion polarity mode using a linear trap quadrupole Velos Pro ion trap mass spectrometer (Thermo Fisher Scientific). The acquisition settings included a full MS1 scan acquisition range of *m/z* 300 to 2000, resolution of *m/z* 0.25 full-width half maximum and a source voltage of +3.2 kV. The automatic gain control for the MS1 scans was set to 5 × 10^4^ with a maximum accumulation time of 50 ms. For the MS/MS events, the resolution was set to *m/z* 0.25 full-width half maximum, the automatic gain control was 2 × 10^4^ and the maximum accumulation time was 300 ms. Data-dependent acquisition was enabled for all samples. The three most abundant precursors in each MS1 full scan were selected for fragmentation using resonance activation (ion trap) collision-induced dissociation at a NCE of 33%. Dynamic exclusion of precursors was inactivated. All MS and MS/MS data were acquired in profile mode. The mass accuracy of the precursor and product ions was typically better than 0.2 Da. The LC-MS/MS instrument was tuned and calibrated, and its performance bench marked using well-characterized bovine fetuin *O-*glycan standards analyzed at the same time as the samples of interest. The generated LC-MS/MS raw data files (made publicly available *via* GlycoPOST (32), accession number GPST000211) were browsed, interrogated, and manually annotated using Xcalibur v2.2 (Thermo Fisher Scientific, https://www.thermofisher.com/), GlycoMod (33), and GlycoWorkBench v2.1 (34) as previously described (35). Briefly, glycans were identified based on the monoisotopic precursor mass, the match between the observed and theoretical MS/MS fragmentation pattern *in silico* generated using GlycoWorkBench, and the relative and absolute PGC-LC retention time of each glycan. Additional support for some structures was obtained using PGC-LC retention time matching of observed platelet *O-*glycans to known bovine fetuin *O-*glycans (36). Further, the reported platelet *O-*glycan structures were backed by observations of identical or similar *O-*glycan structures in the mammalian glycobiology literature. The relative abundances of the confidently identified *O-*glycans were determined from area-under-the-curve measurements based on extracted ion chromatograms performed for all relevant charge states of the monoisotopic precursor *m/z* using Xcalibur v2.2 (Thermo Fisher Scientific).

### MMRN1 Plasmids and Site-Directed Mutagenesis

Mammalian expression vectors were constructed for MMRN1 analysis, which include pcDNA3.1-hMMRN1-Myc-His6 (WT), pcDNA3.1-hMMRN1 T216A-Myc-His6 (made by gene synthesis, Genscript) For the generation of pcDNA3.1-hMMRN1 T1055A-Myc-His6, the T1055A mutation was introduced by PCR using CloneAmp HiFi PCR Premix (Takara Bio Inc) with mutagenic primers 5′-ATGGGGGCGCGTGCATAAATGGAAGAACTAGCTTTACC-3′ and 5′-TATGCACGCGCCCCCATTTTGGCACGGATGC-3′, using the parental plasmid pcDNA3.1-hMMRN1-Myc-His6 as a template. PCR products were digested with DpnI for 1 h at 37 °C to remove the parental plasmid before transformed into DH5α-competent cells (Invitrogen). All mutated plasmids were confirmed by sequencing.

### MMRN1 Secretion Assay

To compare MMRN1 secretion in HEK293T WT, *POFUT1* KO (37), or *POFUT2* KO (38) cells, cells were seeded at 1 × 10^6^ cells per well in 6-well dishes with 2 ml Dulbecco's modified Eagle's medium containing 10% calf serum. Cells were cultured overnight for attachment. The medium was changed to 1 ml Opti-MEM (Invitrogen) before transfection. Cells were transiently transfected using PEI (6 μl PEI per 1 μg plasmid) with 2 μg/well pcDNA3.1-hMMRN1-Myc-His6, 1 μg/well pSecTag-mNOTCH1 EGF1-18-Myc-His6, 1 μg/well pSecTag-hAdamTS9 TSR2-8-Myc-His6, or empty vector, together with 0.1 μg/well IgG plasmid as secretion control. Two days later, culture medium samples were collected. One hundred microliters of medium (250 μl for mNOTCH1 EGF1-18 transfected *POFUT1* KO cells) were precipitated with acetone and loaded onto 4 to 20% SDS-PAGE (Invitrogen), transferred to a nitrocellulose membrane. The membrane was incubated with anti-Myc antibody (Clone 9E10, Invitrogen, 1:2500), and subsequently with IDRye 800–conjugated goat anti-mouse IgG antibody (LI-COR, 1:2500) and IDRye 680–conjugated goat anti-human IgG antibody (LI-COR, 1:2500). The Western blot bands were visualized and quantified using Odyssey System (LI-COR).

For comparing MMRN1 WT with either T216A, or T1055A mutants in HEK293T cells, 1 × 10^6^ cells were seeded in 6-well dishes with 2 ml Dulbecco's modified Eagle's medium containing 10% calf serum. Following overnight attachment, the medium was replaced with 1 ml Opti-MEM. Cells were transiently transfected using PEI (6 μl PEI per 1 μg plasmid) with 2 μg/well pcDNA3.1-hMMRN1-Myc-His6, pcDNA3.1-hMMRN1 T216A-Myc-His6, pcDNA3.1-hMMRN1 T1055A-Myc-His6, or empty vector, together with 0.1 μg/well IgG plasmid as secretion control. Two days later, culture medium samples were collected. 200 μl of medium were acetone-precipitated and subjected to Western blot analysis, following the same methods as described above.

### Experimental Design and Statistical Rationale

All proteomics data were collected across five patients (n = 5), which provides sufficient power to detect significantly secreted proteins from platelets after activation. MMRN1 secretion assays were performed three times with independent transfections (n = 3). Data was analyzed using R (version 4.03, https://www.r-project.org/) and plotted using Tableau (version 2020.4, https://www.tableau.com/products/desktop). Outliers greater than 1.5 times the interquartile range were excluded for some plots to aid visualization. Fold changes were calculated based on median values on a per group basis. Statistical significance was determined using a repeated-measures one-way ANOVA for activation (resting *versus* low/high-dose thrombin), each dose was analyzed separately. Outputs were corrected for multiple testing using the Benjamini–Hochberg correction, with significance being set at *p* <0.05 for an FDR of 5%.

## Results

### Ultrasensitive Platelet Proteome Profiling

**Fig. 1:**
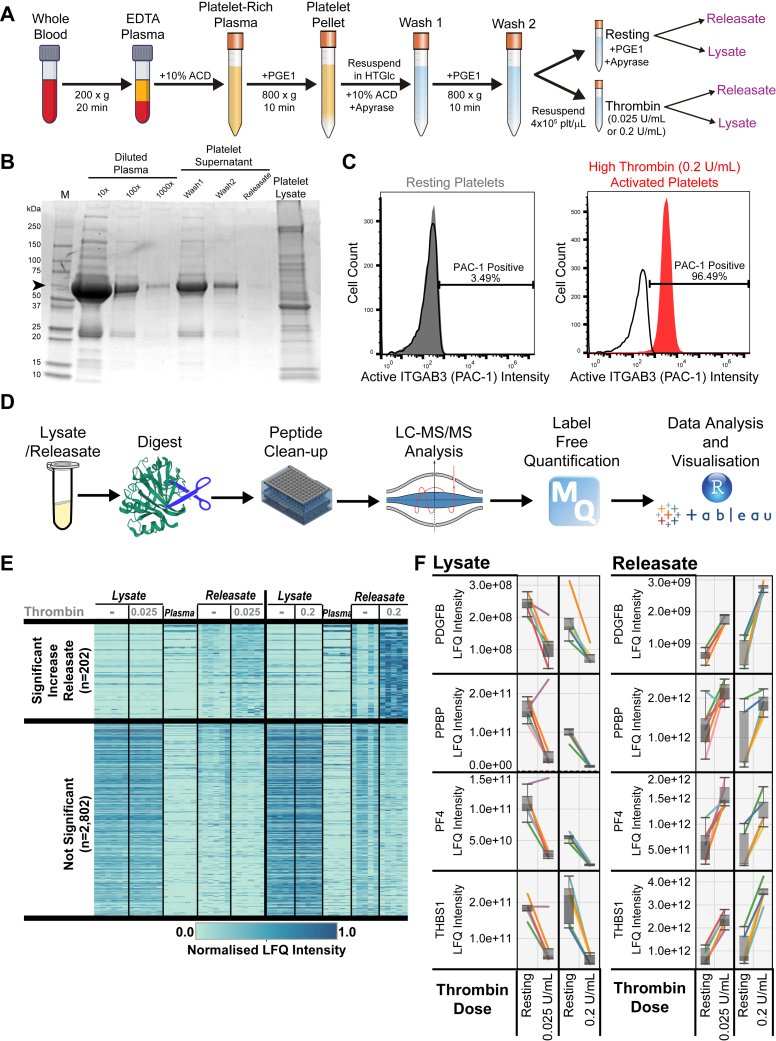
**Proteomic analysis of platelet lysates and releasates from healthy donors.***A*, workflow for platelet isolation and stimulation with thrombin. *B*, Coomassie stained SDS-PAGE gel to assess for plasma contamination in platelet lysates and releasates. *Arrow* indicates albumin. *C*, histograms of platelet activation using PAC-1 intensity (*x*-axis). Resting platelets are shown in *gray*, platelets stimulated with either low-dose (0.025 U/ml) or high-dose (0.2 U/ml) thrombin shown in *red*. *D*, workflow for lysate/releasate proteomic analysis. *E*, heat map showing platelet proteins that were significantly increased in the releasate by high-dose thrombin stimulation or not significantly regulated, n = 5. *F*, *boxplots* of proteins known to be secreted by platelets after thrombin activation. Each line represents a single donor. The *y*-axis shows the label-free quantitation (LFQ) intensity for each protein.

To examine the platelet proteome with high fidelity, we have employed a platelet isolation method that reduces aberrant platelet activation and minimizes cellular/plasma contamination (Fig. 1, *A* and *B* and supplemental Fig. S1*A*). Based on our thrombin-stimulation dose-response curve for these platelet preparations (supplemental Fig. S1*B*) we defined two doses of thrombin to achieve both low- (0.025 U/ml) and high- (0.2 U/ml) thrombin stimulation. To confirm platelet activation, we analyzed PAC-1 (Fig. 1*C* and supplemental Fig. S1*C*) and P-selectin (supplemental Fig. S1*D*) expression of these cells using flow cytometry. High-dose thrombin yielded >98% P-selectin^+^ and ∼92% PAC-1^+^ cells. In contrast, low-dose thrombin yielded >40% P-selectin^+^ and ∼30% PAC-1^+^ cells.

MS-based proteomics was used to examine the platelet lysates and corresponding releasates (Fig. 1*D*). Analysis of the platelet releasates identified >1300 proteins consistently detected across both the high- and low-dose groups (supplemental Table S1). From the platelet lysates >3000 proteins were consistently detected (supplemental Table S1). To visualize the thrombin-induced proteome changes, we plotted a heat map of the normalized LFQ intensity for all proteins detected (Fig. 1*E*). From this heat map the difference in protein releasate abundance of the high-thrombin–treated platelets is clearly apparent. Proteins known to be released upon thrombin activation, showed the expected responses after thrombin stimulation (Fig. 1*F*).

### Unbiased PTM Analysis of Releasate Proteins

**Fig. 2:**
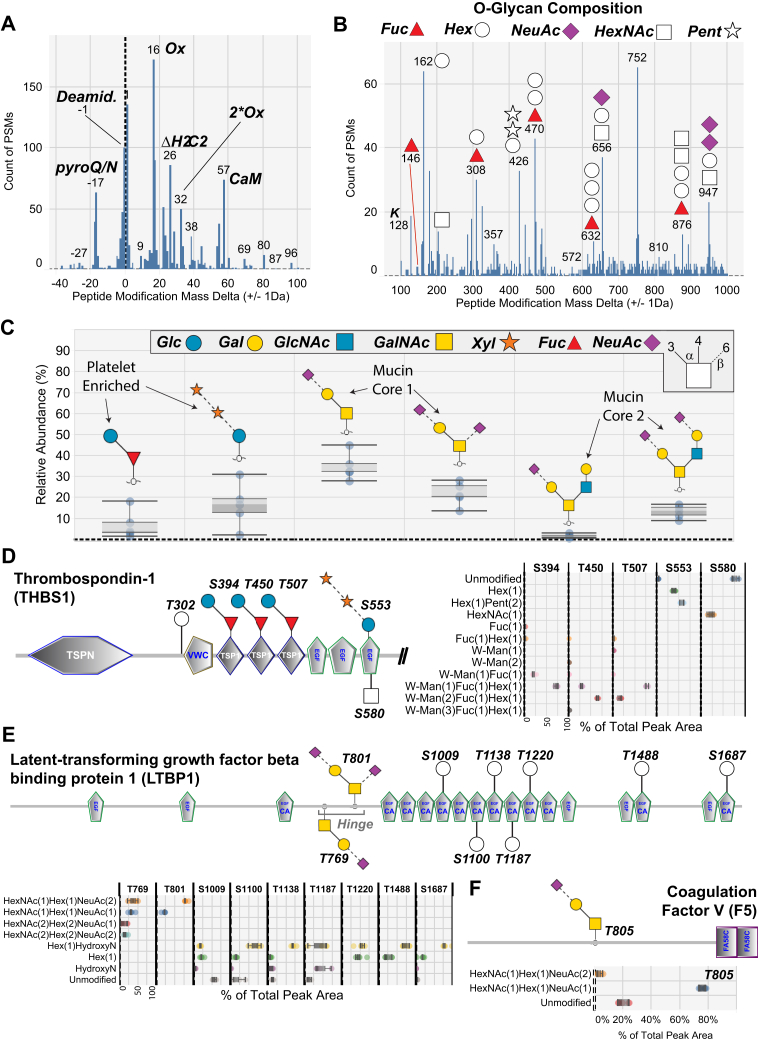
**Unbiased detection of protein modifications including *O-*glycosylation of platelet releasates.** Histograms of the open-search analysis of high-dose thrombin stimulated platelet releasates showing the number of peptide spectral matches (PSMs) (*y*-axis) across all platelet samples identified as having a range of mass adducts (*x*-axis, each bar represents a 1 Th wide mass adduct bin). PSMs with a Byonic log probability (Log Prob) score >8 and had modifications to S,T,Y,K,R,D,E,N,Q,P,M,W were plotted. Modifications were plotted separately for common low-mass modifications (*A*) and higher mass modifications (*B*). Delta masses corresponding to *O-*glycan masses are indicated by the glycan symbols. *C*, quantitative *O-*glycomics analysis of *O-*glycans detached by β-elimination from platelet releasate proteins as described in Supplementary methods (n = 5). Bond linkage types are indicated in the legend. The identified O-glycans are depicted as relative abundances out of all observed O-glycans (100%). *D*, *boxplots* showing quantitative analysis of thrombospondin-1 *O-*glycosylation at identified sites, (n = 5). W-Man is C-mannose modified tryptophan on the same peptide as the indicated Ser/Thr. *E*, *boxplots* showing quantitative analysis of latent-transforming growth factor beta-binding protein 1 *O-*glycosylation at identified sites, (n = 5). HydroxyN is hydroxylated asparagine on the same peptide as the indicated Ser/Thr. *F*, *boxplots* showing quantitative analysis of coagulation factor V *O-*glycosylation at identified sites, (n = 5).

PTMs are known to play important roles in the function of platelet releasate proteins. For example, the *O-*glycosylation of thrombospondin 1 (THBS1) has been proposed to mediate protein–protein interactions and protein folding (39). To investigate modifications to proteins in the releasate proteome, we employed an open-search strategy as described previously (29, 40). Peptide-level MS/MS data from all thrombin-treated platelet releasates were combined and searched for modifications ranging in mass from −40 to +1000 Da, representing a wide range of potential PTMs (supplemental Table S2). As expected, we detected many chemical modifications of <100 Da arising during sample preparation, such as oxidation (+16 Da) (Fig. 2*A*). However, it should be noted that several modification masses of ∼80 Da were observed and may correspond to biologically relevant modifications on secreted platelet proteins such as sulfation.

Unexpectedly, plotting peptide modifications >100 Da revealed ∼20 prominent peaks, whose masses corresponded with known *O-*glycan compositions and were associated with Ser/Thr residues (41) (Fig. 2*B*). To confirm this, an *O*-glycan variable modification search was performed on the platelet releasate LC-MS/MS data (supplemental Table S3). This search used a glycan database that was a combination of well-known mammalian glycans and any glycan that was putatively identified in the open search analysis (supplemental Table S2). While some of these glycans are not known to occur in mammals, we believe the unbiased analysis of the dataset is useful. Modifications containing fucose, hexose, pentose, and HexNAc units and combinations of these were confidently identified (42). A comparison of the open-search and *O*-glycan specific search demonstrated ∼45% overlap between these approaches for the most frequently identified *O*-glycans (supplemental Fig. S2 and Table S3). Two modifications had compositions consistent with mucin-type sialo*-O-*glycans. Peptides modified by fucose alone (146 Da) were identified, alongside many peptides modified by a single hexose (162 Da). In total 11 platelet-derived proteins were identified as being *O-*glycosylated, including a small group of proteins modified at multiple sites, including THBS1, latent-transforming growth factor beta-binding protein 1 (LTBP1), fibrinogens, and platelet basic protein. The discovery that these proteins carry *O-*glycans is important as this small protein subset constitutes >50% of the protein content released by platelets (supplemental Table S1). Importantly, it should be noted that assignment of glycosylation site localization is extremely difficult using HCD data alone, thus all sites given in supplemental Table S3 have been marked as ambiguous in their glycosylation site localization within each peptide. Many aspects of the search can decrease the accuracy of glycosylation site analysis, including nonspecific cleavages, fortuitous side reactions, the mixing up of N/C/O-glycosylation, and potential adduct formation.

The corresponding *O-*glycan profile was established by quantitative *O-*glycome analysis, where all *O-*glycans are detached through β-elimination from already de-*N*-glycosylated proteins and profiled by LC-MS/MS (35) (Fig. 2*C*). This *O-*glycome profiling method provides both the fine structures and relative abundances of *O-*glycans. *O-*Glycans with 11 different structures covering nine different compositions were identified as the main components attached to platelet releasate proteins (supplemental File 2). Four prominent glycans quantitatively agreed with our open search analysis of the proteomics data, as we observed a high abundance of *O-*fucose structures extended by glucose, (xylose)_2_-glucose conjugates, and mucin-type core 1 and 2 sialo*-O-*glycans (supplemental Table S4). Since PGC is known to be unable to retain monosaccharides, we were not able to observe the monosaccharides modifications (Hex, dHex, HexNAc) as indicated in the open search and conversely since the open search was capped at 1000 Da, a few larger penta- and hexa-saccharide glycans were not identified in that analysis while still being detected in our glycomics data. Further, the open search pointed to the existence of FucHex2/3 modifications; however, since these modifications were not identified in the matching glycomics analysis, these peptide modifications may instead arise from a combination of two or more PTMs, that is, C-mannosylation, *O*-Fuc.

Of the *O-*glycosylated sites detected on releasate proteins many were not previously observed in human samples including platelets. To validate these PTMs, we performed EThcD fragmentation analysis on a separate cohort of platelet samples (supplemental Table S5). EThcD fragmentation–based MS/MS analysis is known to preserve labile modifications such as *O-*glycans for more confident identification (43). A search was performed on these EThcD data with the same parameters as used for the HCD data analysis. However, the glycosylation site search of EThcD data can still be complicated by the same factors as HCD analysis. Thus, only those glycosylation site localizations that have been validated through manual interpretation of the MS/MS spectra have been labeled as having unambiguous glycosylation site localization in supplemental Table S5. Among the many releasate proteins confirmed to be *O-*glycosylated, four modified proteins were the most interesting for platelet biology and were annotated in the context of their known domains and modification sites (39, 44, 45, 46, 47, 48). Extracted ion chromatograms were generated and peak areas determined for each intact glycopeptide ion to compare the microheterogeneity at each modified site. It should be noted that this analysis is semiquantitative, given that the ionization efficiency of each form of glycopeptide will be different. First, we observed that ∼53% of S553 of THBS1 had a (pentose)_2_-hexose modification consistent with a (xylose)_2_-glucose conjugate (Fig. 2*D*). Second, we observed two sialylated mucin-type *O-*glycans on LTBP1 at T769 and T801 within the proteolytically sensitive hinge domain, which targets the protein to the extracellular matrix and is needed for transforming growth factor beta release (49) (Fig. 2*E*). Third, coagulation factor V (F5) carried mostly a core 1 mucin *O-*glycan (>75% sialyl T) in position T805, while ∼20% of the site was unmodified (Fig. 2*F*). Lastly, we observed >95% *O-*fucosylation of MMRN1 at T216, which is outside the epidermal growth factor (EGF)-like and thrombospondin type 1 repeats (TSR) domains known to contain this modification (50).

### Quantification of Releasate Proteins

**Fig. 3:**
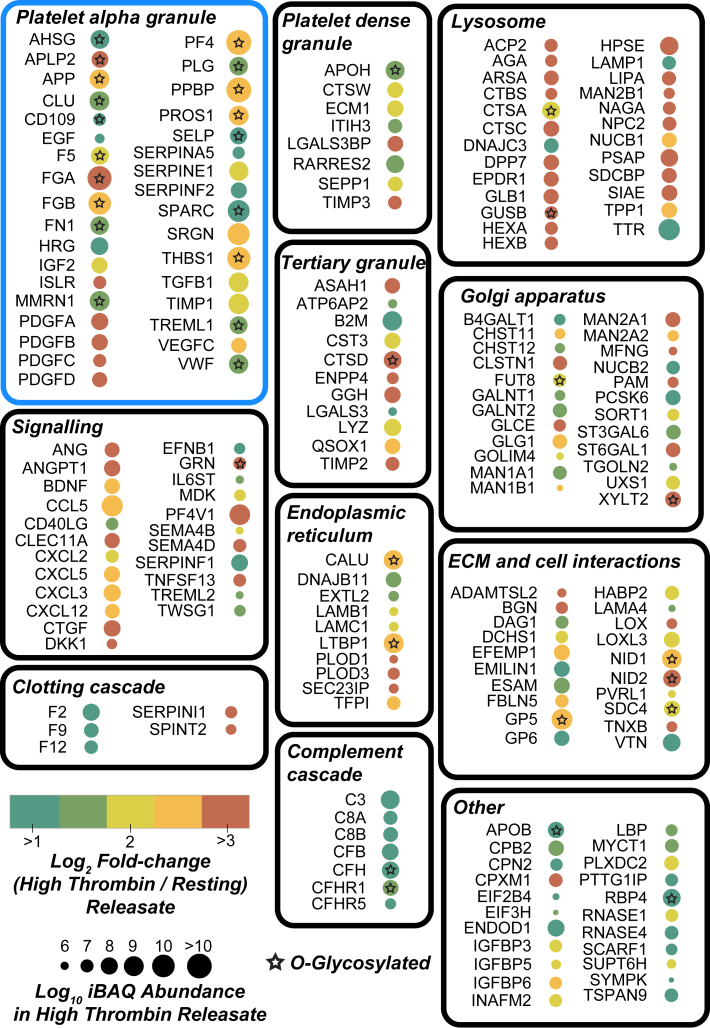
**Functional grouping and response of platelet proteins significantly increased in the releasate after high-dose thrombin stimulation.** Each protein is represented by a circle annotated with the gene name, and the circle color represents the log_2_ fold change (thrombin stimulated/resting) in the releasate (*i.e.*, the degree of change). The *circle siz*e represents the log_10_ iBAQ abundance of each protein in the high-dose thrombin stimulated releasate (*i.e.*, the proportion of the protein relative to the total proteins). *Stars* indicate releasate proteins that were identified to be *O-*glycosylated.

**Fig. 4:**
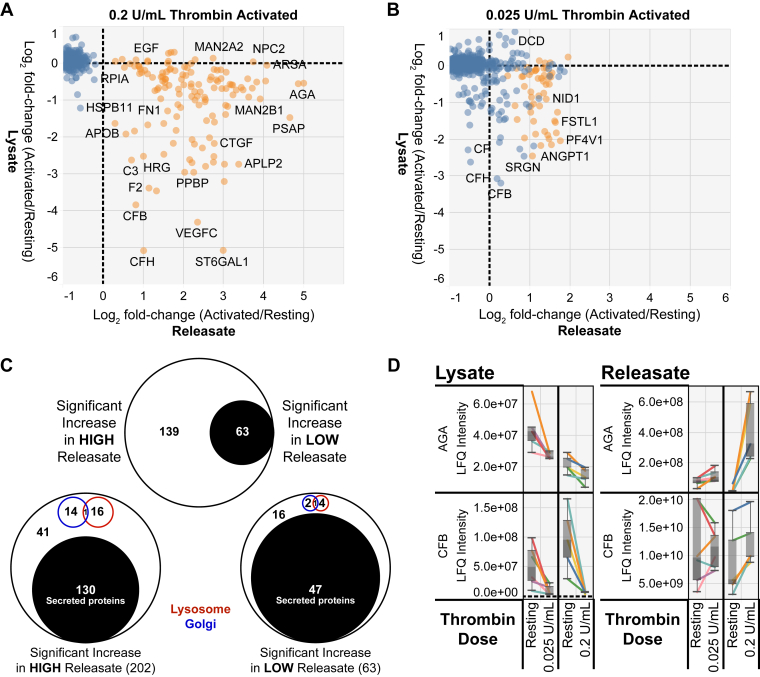
**Comparison of platelet lysate and releasate proteomes for confident detection of released proteins from thrombin-activated platelets.** Scatterplot of proteins significantly regulated in the high-dose (*A*) and low-dose (*B*) thrombin-stimulated releasate. Significantly regulated proteins are shown in *orange*, nonsignificant proteins are shown in *blue*. The log_2_ fold change (thrombin stimulated/resting) for both the each corresponding releasate (*x*-axis) and the corresponding lysate (*y*-axis) was used for plotting. *C*, *Venn diagrams* indicating the overlap between significantly increased releasate proteins in either the low-dose or high-dose thrombin groups, *top*. Analysis of the proportion of significantly increased releasate proteins that are annotated by UniProt as secreted, Golgi-associated, lysosome-associated, or other. *D*, *boxplots* of proteins significantly regulated in platelet releasates-only (AGA) or lysates-only (CFB) after thrombin activation. Each line represents a single donor. The *y*-axis shows the label-free quantitation (LFQ) intensity for each protein.

Overall, we identified 202 proteins with significantly increased total abundance in the high-dose thrombin releasates, whereas only 63 were significantly increased by the low-dose thrombin (supplemental Table S6). To delineate the functional groups of the proteins increased in high-dose thrombin releasates, we categorized proteins into ten distinct gene ontology groups based on either biological process or cellular component (Fig. 3). As expected, proteins known to reside in the platelet alpha granules underwent the largest fold change increase and were also the most abundant releasate constituents. This was closely followed by proteins involved in cellular signaling such as chemokines and growth factors (*e.g.*, C-X-C motif chemokine 3, C-C motif chemokine 5). In this signaling group, several proteins not previously described to be released from platelets were detected, including granulin (GRN) and midkine (MDK), with GRN having the largest fold change and final protein abundance. The group showing the largest average fold change were from the lysosome (51). Many proteins from the Golgi and endoplasmic reticulum were also observed in the releasate, a large proportion of which are involved in glycan processing/metabolism (52). Of the 133 proteins having accurate measurements of lysate fold change, only 44 decreased in lysate abundance after high thrombin by >2-fold (Fig. 4, *A* and *B* and supplemental Table S6). Several lysosomal proteins that were enriched in releasates (Fig. 4*C*), such as N4-beta-N-acetylglucosaminyl-L-asparaginase showed a 32-fold increase in platelet releasates after thrombin, but only <2-fold decrease in their lysate abundance (Fig. 4*D*). This suggests a large pool of these proteins likely remains within the cell and that only a small portion is secreted after thrombin treatment. Lastly, proteins that mediate cell–cell contacts and comprise the extracellular matrix were also released and included proteins such as glycoprotein V (GP5) (53) and nidogen 1, a basement membrane component.

### Characterization of MMRN1 Fucosylation Within the EMI Domain

**Fig. 5:**
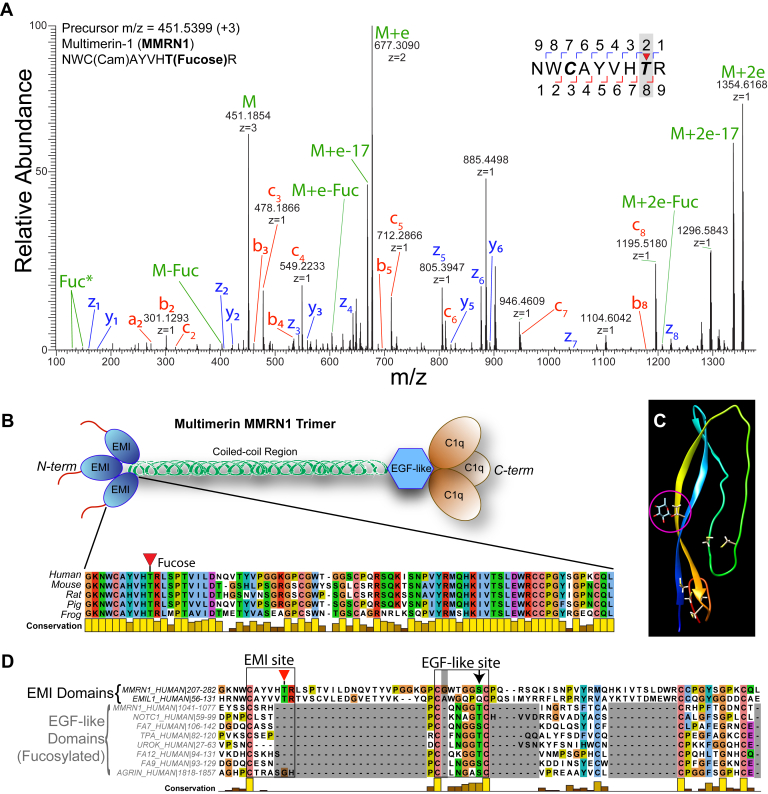
**Identification of a novel *O-*fucosylation site on platelet multimerin 1.***A*, electron transfer higher collision energy dissociation (EThcD) mass spectrum of the novel *O-*fucosylation site at T216 in MMRN1. The intact precursor ions and associated neutral losses are in *green*, the z and c fragment ion series are shown in *blue* and *red*, respectively. *B*, *cartoon* of the trimeric MMRN1 structure with key domains shown. *Inset*, protein sequence alignment of the MMRN1 EMI domain across diverse species. *C*, structural prediction of the EMI domain from RoseTTafold with cysteine residues shown as *ball* and *stick* and the fucosylated threonine residue highlighted inside a *pink circle*. Cysteine residues are highlighted by showing the side-chain atoms. *D*, protein sequence alignment of human EMI domains and EGF-like domains that are known to be *O-*fucosylated by POFUT1. The EMI site is highlighted with a *red triangle*, indicating the site of *O-*fucosylation. The EGF-like site is highlighted with the *black arrow*, indicating the modified residue in EGF-like domains. EGF, epidermal growth factor; EMI, elastin microfibril interface; POFUT1, protein *O-*fucosyltransferase 1.

One of the most abundant proteins released by activated platelets was MMRN1, a large ∼150 kDa protein that initially forms stable homotrimers, which are hypothesized to interact through EMI domain and C1q domain binding to form elongated multimers of up to many MDa in size (54). We observed a novel fucosylation of MMRN1 at T216, which is within the N-terminal EMI domain of the protein. The *O-*fucosylation on T216 of MMRN1 was confirmed by EThcD analysis as was the predicted *O-*fucosylation of the C-terminal EGF-like domain at T1055 (Fig. 5*A* and supplemental Table S5). The stoichiometry of T216 fucosylation is very high, as the fucosylated peptide was >10-fold more intense compared to the nonglycosylated form (supplemental Table S2). The modified T216 and its surrounding sequence are highly conserved in vertebrates (Fig. 5*B*). The EMI domain has six conserved cysteines, which likely form disulfide bonds (55). To examine the predicted 3D-structure of the MMRN1 EMI domain we used the RoseTTAfold program (56), which predicted the correct pairing of cysteine residues (57) (Fig. 5*C*). Mapping the modified threonine residue onto this structure illustrates the small size of the EMI domain relative to the fucose modification (*pink circle*, Fig. 5*C*). The EMI domain is hypothesized to contain two subdomains, where one is similar to the C-terminal region of the EGF-like domain (55). EGF-like domains are known to be *O-*fucosylated by the endoplasmic reticulum–resident enzyme GDP-fucose protein *O-*fucosyltransferase 1 (POFUT1) (58). *O-*Fucosylation of EGF-like domains by POFUT1 occurs in a specific motif (C^2^XXXX[S/T]C^3^) bracketed by the second and third conserved cysteine residues (59, 60, 61). Mutation of the modified Ser/Thr within this motif completely blocks *O*-fucosylation by POFUT1 (62, 63). Cocrystal structures of POFUT1 and an EGF repeat with this motif shows that the folded EGF repeat is bound in such a way to position the hydroxyl of Ser/Thr precisely in the active site for a nucleophilic attack on the anomeric carbon of fucose of GDP-fucose (64). In contrast, the related enzyme POFUT2 adds *O-*fucose to TSRs containing CXX[S/T]C motifs such as THBS1 as shown in Figure 2*D* (65). Sequence similarities were observed after alignment of the MMRN1 EMI-domain with EGF-like domains that are known to be *O-*fucosylated by POFUT1 (Fig. 5*D*). This showed T216 of MMRN1 is in a region of the EMI domain not conserved with EGF-like domains but displays a similar sequence motif that is only missing the C-terminal cysteine residue and would be compatible with transfer of fucose (64). The region of MMRN1’s EMI domain that is homologous to the EGF-like domain fucosylation site does not match the POFUT1 modification motif, as one extra amino acid has been inserted and this is known to be incompatible with fucosylation (64). Therefore, this modification of MMRN1 at T216 may represent a new recognition motif for POFUT1 within a new domain type that is missing the C-terminal cysteine residue.

**Fig. 6:**
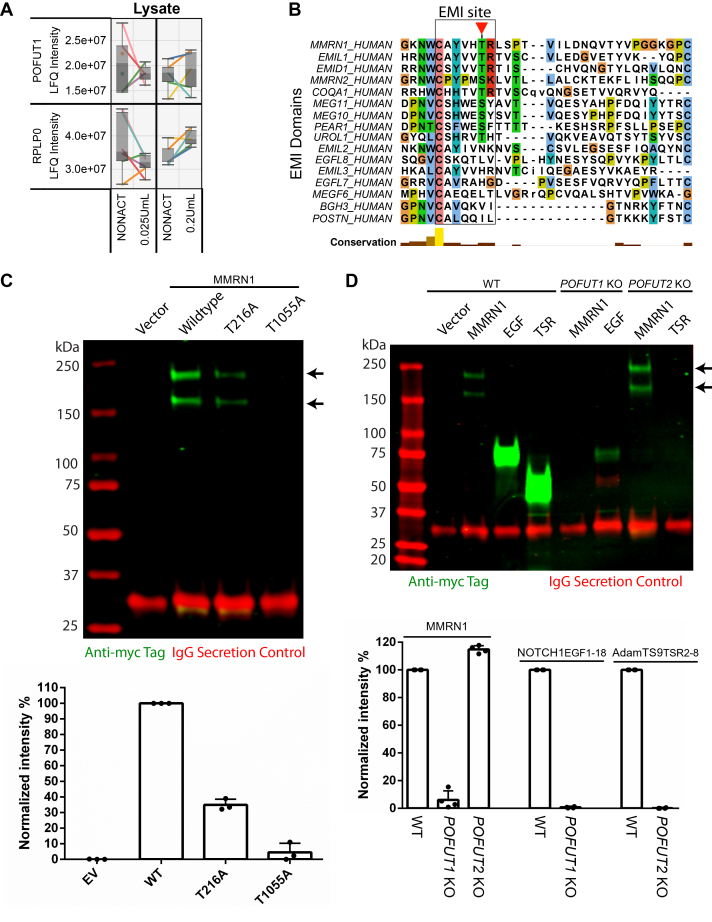
**Loss of POFUT1 affects multimerin-1 secretion.***A*, *boxplots* of POFUT1 and the negative control RPLP0 (an abundant ribosomal protein) in platelet lysates before and after thrombin activation. Each line represents a single donor. The *y*-axis shows the label-free quantitation (LFQ) intensity for each protein. *B*, protein sequence alignment of EMI domains across a wide range of human proteins. The EMI site is highlighted with a *red triangle*, indicating the site of *O-*fucosylation. *C*, *top*, HEK293T cells were transfected with plasmids encoding MMRN1 WT-Myc, MMRN1 T216A-Myc, MMRN1 T1055A-Myc, or empty vector (EV) and IgG (secretion control). Cells were cultured for 2 days. Cultured medium was collected and analyzed by Western blot probed with anti-Myc and anti-human IgG antibodies. *Bottom*, the *bar graph* shows quantified band intensity normalized with IgG bands from three independent transfection experiments (n = 3), plotted as mean ± SD. *D*, *top*, HEK-293T cells either WT, *POFUT1* KO, or *POFUT2* KO were transfected with plasmids encoding MMRN1-Myc, NOTCH1 EGF1-18-Myc, or AdamTS9 TSR2-8-Myc and IgG (secretion control). After 2 days, cultured medium was collected and analyzed by Western blot probed with anti-Myc and anti-human IgG antibodies. *Bottom*, the *bar graph* shows quantified band intensity normalized with IgG bands from three independent transfection experiments (n = 3), plotted as mean ± SD. EGF, epidermal growth factor; EMI, elastin microfibril interface; POFUT1, protein *O-*fucosyltransferase 1; TSR, thrombospondin type 1 repeats.

Using our proteomics dataset, POFUT1 was detected in the platelet lysates and underwent no significant change in abundance after thrombin stimulation (Fig. 6*A*). To determine the extent of possible POFUT1 targets, we aligned all human EMI domains (Fig. 6*B*). This showed EMILIN1, MMRN2, EMI domain–containing protein 1, and collagen alpha-1(XXVI) chain had the highest similarity and are also potential *O-*fucosylation targets. The *O-*fucosylation site within MMRN1 at T216 is a highly conserved position across EMI domains as it is present in approximately half the proteins. This suggests EMI domains are a previously uncharacterized target of *O-*fucosylation and that these modifications may play a key role in the function of EMI domain–containing proteins. A known function of *O-*fucosylation is to assist protein folding and aid in secretion (50, 65). Consistent with this, exogenous expression in HEK293T cells of a MMRN1 T1055A mutant (EGF *O-*fucose–deficient) showed a nearly complete loss of MMRN1 secretion. Notably, expression of a MMRN1 T216A mutant (EMI *O-*fucose–deficient) led to a >50% reduction in secretion, suggesting a role of EMI *O-*fucose in assisting MMRN1 secretion (Fig. 6*C*). To examine the role of POFUT1 in MMRN1 secretion, we expressed myc-tagged WT MMRN1 in HEK293T cells that were either WT, *POFUT1*-null mutants, or *POFUT2*-null mutants. Secretion of positive control domains, EGF-like and TSR, which require *O-*fucosylation for secretion, were absent from the media in *POFUT1* KO and *POFUT2* KO cells, respectively (POFUT1 does not modify TSRs and POFUT2 does not modify EGF repeats) (66, 67). Secreted MMRN1 could be detected in the tissue culture media from WT and *POFUT2*-null cell lines, but was not detected in the *POFUT1*-null cells (Fig. 6*D*). Since loss of *O-*fucose on T216 resulted in >50% loss of secretion, these data suggest that POFUT2 is not responsible for modifying the EMI domain, leading to the conclusion that either POFUT1 or a novel POFUT is responsible for this modification.

## Discussion

In this study, we mapped thrombin-induced platelet responses by combining high-quality platelet preparations and ultrasensitive MS-based proteomics and glycomics. This enabled the identification of new factors being released by platelets and observation of novel PTMs on platelet proteins. Our analysis generated three key findings. First, platelet releasate proteins were enriched for a wide range of *O-*glycan modifications and we quantified many novel modification sites and structures. Second, the abundant platelet releasate protein MMRN1 was determined to be *O-*fucosylated on a novel site (T216) within its EMI domain, and we show that the T216A mutation resulted in >50% reduction in MMRN1 secretion. We propose the *O-*fucosylation of the EMI domain is catalyzed by either POFUT1 or a novel (unknown) POFUT. Lastly, large differences in platelet secretion response were observed between low-dose and high-dose thrombin, with strong stimuli triggering increased secretion of lysosomal luminal enzymes. Collectively, these data provide an extensive proteome-wide analysis of platelet responses to thrombin activation, which may contribute to disease states through new functions in haemostasis. This proteomic resource is provided as a free web-based interactive visualization for the research community (larancelab.com/platelet-proteome).

*O-*Glycosylation is known to play an important regulatory role in cellular function; however, the majority of platelet studies have focused on the role of N-glycans and *O-*sialic acids in platelet production and clearance (68, 69). Importantly, defective *O-*glycosylation of platelet proteins results in a bleeding phenotype (70). Previous studies have used enrichment regimes to facilitate the detection of platelet *O-*glycosylation (71). However, these regimes often only allow the identification of specific glycan classes (*e.g.*, *O-*GalNAc glycans) and do not enable identification of some platelet-enriched modifications such as *O-*fucosylation or *O-*glucosylation. Here, we demonstrate that many of the released platelet proteins carry diverse *O-*glycan structures. This included high abundance of *O-*fucosylation and *O-*glucosylation and their extended forms, alongside the more broadly expressed mucin-type glycans. The high abundance of *O-*fucosylated proteins can be explained through domain enrichment analysis of the >200 platelet releasate proteins we detected using STRING-db (72), where 19 proteins (*e.g.*, THBS1, MMRN1, LTBP1, nidogen 1) contained either EGF-like domains or TSRs, which are the only previously known consensus sites for *O-*fucosylation. This study has now demonstrated for the first time that sites that were only predicted to be modified, such as the EGF-like domain in MMRN1 (50), are indeed *O-*fucosylated at high stoichiometry in human platelets. This likely contributes to platelet function as demonstrated by the loss of MMRN1 secretion when this domain cannot be *O*-fucosylated as shown by a T1055A mutant.

We have discovered that the abundant platelet releasate protein MMRN1 is constitutively *O-*fucosylated at T216 within its EMI domain. MMRN1 is known to have several functions including binding/release of coagulation factor V, thrombus formation (73), and interaction with collagen *via* an N-terminal RGD motif (74). We show that T216 was stoichiometrically fucosylated within the context of a potentially altered POFUT1 *O-*fucosylation motif (C^1^-X-X-X-X-T-X) derived from its known modification context in EGF-like domains (64). Mutation of the EMI *O-*fucose site led to >50% reduction in MMRN1 secretion. These results indicate a role for EMI *O-*fucosylation in regulating MMRN1 secretion. The fact that elimination of POFUT2 does not reduce MMRN1 secretion strongly suggests EMI *O-*fucosylation is mediated by either POFUT1 or a novel POFUT. EMI *O-*fucosylation may be required for correct folding of the tertiary or quaternary structure of MMRN1, which exists natively as a trimer (54). Given the proposed role of the N-terminal EMI domain to interact with the C-terminal C1q domain and enable MMRN1 multimerization (55), we propose that *O-*fucosylation is required for this interaction and is important for MMRN1 secretion and possibly multimerization efficiency.

High sensitivity MS-based proteomics of the platelet releasate has enabled us to identify several novel factors released from platelets in response to high-dose thrombin. These include two small, secreted proteins GRN, and MDK. Given the known links between lysosomal function and GRN activity (75, 76), we hypothesise that GRN plays a key role in the formation of the platelet lysosomes. MDK signaling through a range of receptors is known to control inflammatory processes, including the activation of neutrophils (77). Therefore, we hypothesize that the release of the proinflammatory MDK cytokine by platelets contributes to inflammation by neutrophil recruitment at the site of tissue damage and, in turn, enhances the innate immune defence.

This work catalogues the proteome-wide response of human platelets to thrombin activation. Platelets experience a range of thrombin levels *in vivo*; the thrombin concentrations during coagulation are estimated to range from 1 nM (0.1 U/ml) to over 500 nM (78). Different thrombin concentrations will also affect the platelet interacting environment, including the composition of fibrin strands (79). Alterations in the platelet releasate content in response to different thrombin concentrations may differentially modulate platelet functions, including immune, inflammatory, angiogenic, and tissue remodeling responses (80).

We show that high-dose (0.2 U/ml) thrombin can trigger the release of many proteins associated with the secretory pathway including the ER, Golgi, and lysosome. Our comparison of platelet lysate and releasate fold changes after thrombin treatment, showed that only a small pool of these secretory pathway proteins was released from platelets. Furthermore, several low abundance proteins detected in thrombin-stimulated releasates such as tumor necrosis factor ligand superfamily member 13 and calsyntenin-1 could not be reliably detected in the corresponding lysates (81, 82). Therefore, these proteins are likely of very low abundance within platelet cells and would have been overwhelmed by more abundant proteins during LC-MS/MS analysis of platelet lysates. In contrast, the simpler releasate protein mixture exhibits a lower dynamic range, which facilitates detection of minor protein components with high sensitivity. This demonstrates the advantage of platelet releasate analysis compared to analysis of lysates alone.

In conclusion, the multidimensional dataset we provide here on the platelet proteome provides the groundwork for future mechanistic studies investigating the functions of the novel proteins identified and the previously uncharacterized *O-*glycosylation sites. Analysis of how the platelet proteome is altered in disease states such as type II diabetes, where platelets are known to be hyperactivated (83), should prove fruitful to detect new mechanisms of platelet dysfunction. In addition to the abundant *O-*glycosylation decorating the platelet releasate proteins, many other PTMs remain to be explored in the context of platelet function and hemostasis.

## Data Availability

Raw MS data have been deposited to the ProteomeXchange Consortium (http://proteomecentral.proteomexhange.org) *via* the PRIDE partner repository with the dataset identifier PXD045535 (Username: reviewer_pxd045535@ebi.ac.uk, Password: 8rjlqMU2). The *O-*glycan LC-MS/MS raw data files are made publicly available *via* GlycoPOST(36), accession number GPST000211.

## Supplemental Data

This article contains supplemental data.

## Conflict of interest

The authors declare no competing interests.

## Supplemental Data

Supplemental File 1 Platelet releasate database for open search with decoys.

Supplemental File 2 Annotated HCD fragmentation MS/MS spectra for identified *O*-glycosylated peptides.

Supplemental Table S1 Platelet lysate and releasate proteome quantification data table.

Supplemental Table S2 Peptide identification data for the open-search detection of modified platelet releasate proteins.

Supplemental Table S3 HCD Peptide identification data for the *O*-glycosylation search of platelet releasate proteins.

Supplemental Table S4 *O-*Glycome profiling of platelet releasate proteins.

Supplemental Table S5 Peptide identification data for EThcD analysis of platelet releasate proteins.

Supplemental Table S6 Platelet releasate proteins significantly increased after thrombin stimulation.

Supplemental Figure 2

Supplemental File 3 PGC-LC-MS/MS-based *O-*Glycan characterisation including annotated spectra and peak quantification.

Supplemental File 4 Annotated EThcD fragmentation MS/MS spectra for identified *O*-glycosylated peptides.
