# Supplementary material for: Analysis of the Healthy Platelet Proteome Identifies a New Form of Domain-Specific O-Fucosylation
Source: Mol Cell Proteomics. 2024 Jan 16;23(2):100717. doi: 10.1016/j.mcpro.2024.100717 (PMC10879016; doi:10.1016/j.mcpro.2024.100717)
Supplement: Supplemental File 2 — Annotated HCD fragmentation MS/MS spectra for identified O-glycosylated peptides. [file mmc2.pdf]

Supplementary File 2

Annotated HCD fragmentation MS/MS spectra for identified *O*-glycosylated peptides.

Fragmentation Key:

Each MS/MS spectrum is annotated in the top left corner in the following format:
(GENE)_(Modified residue)_(Glycosylation modification)

Ions in the MS/MS spectrum are annotated as either:

- y-ions
- b-ions
- a-ions

- ~y-ions with a neutral loss (e.g. whole glycan loss)
- ~b-ions with a neutral loss (e.g. whole glycan loss)
- ~a-ions with a neutral loss (e.g. whole glycan loss)

- M (intact precursor)
- Pep_2+ (intact precursor that has undergone a neutral loss)
- HexNAc (oxonium and immonium ions)

- Mass error for fragment matching is <20 ppm
- Annotated spectra where insufficient ions were available to confirm *O*-glycosylation site localisation have been annotated with the text "Ambiguous site localization".

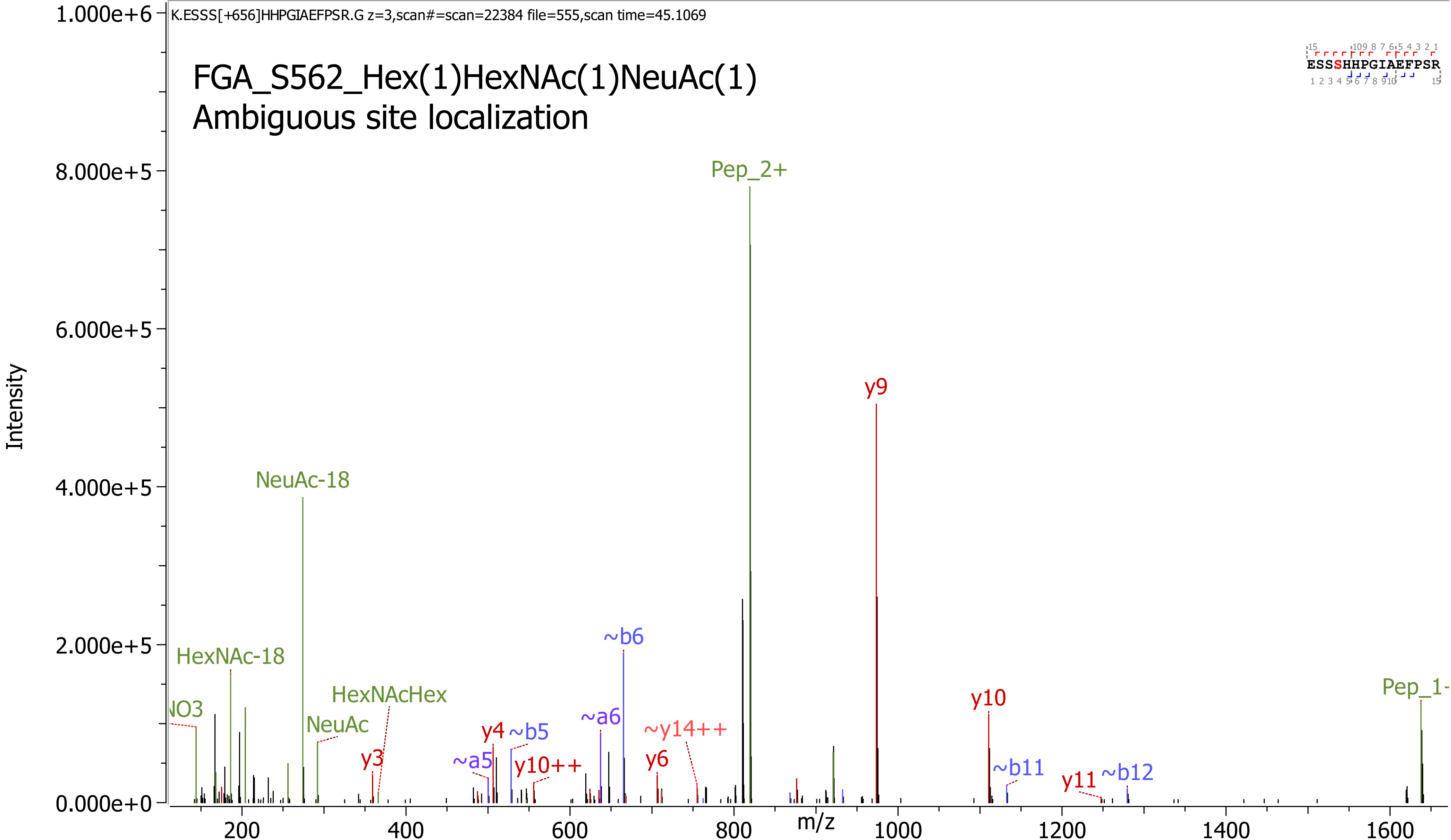
K.ESSS[+656]HHPGIAEFPSR.G z=3,scan#=scan=22384 file=555,scan time=45.1069
FGA_S562_Hex(1)HexNAc(1)NeuAc(1)
Ambiguous site localization
ESSSHHPGIAEFPSR
Intensity
m/z
Pep_2+
y9
NeuAc-18
~b6
HexNAc-18
HexNAcHex
NeuAc
y10
Pep_1-
~a6
~y14++
y4
~b5
~a5
y3
y10++
y6
~b11
~b12
y11

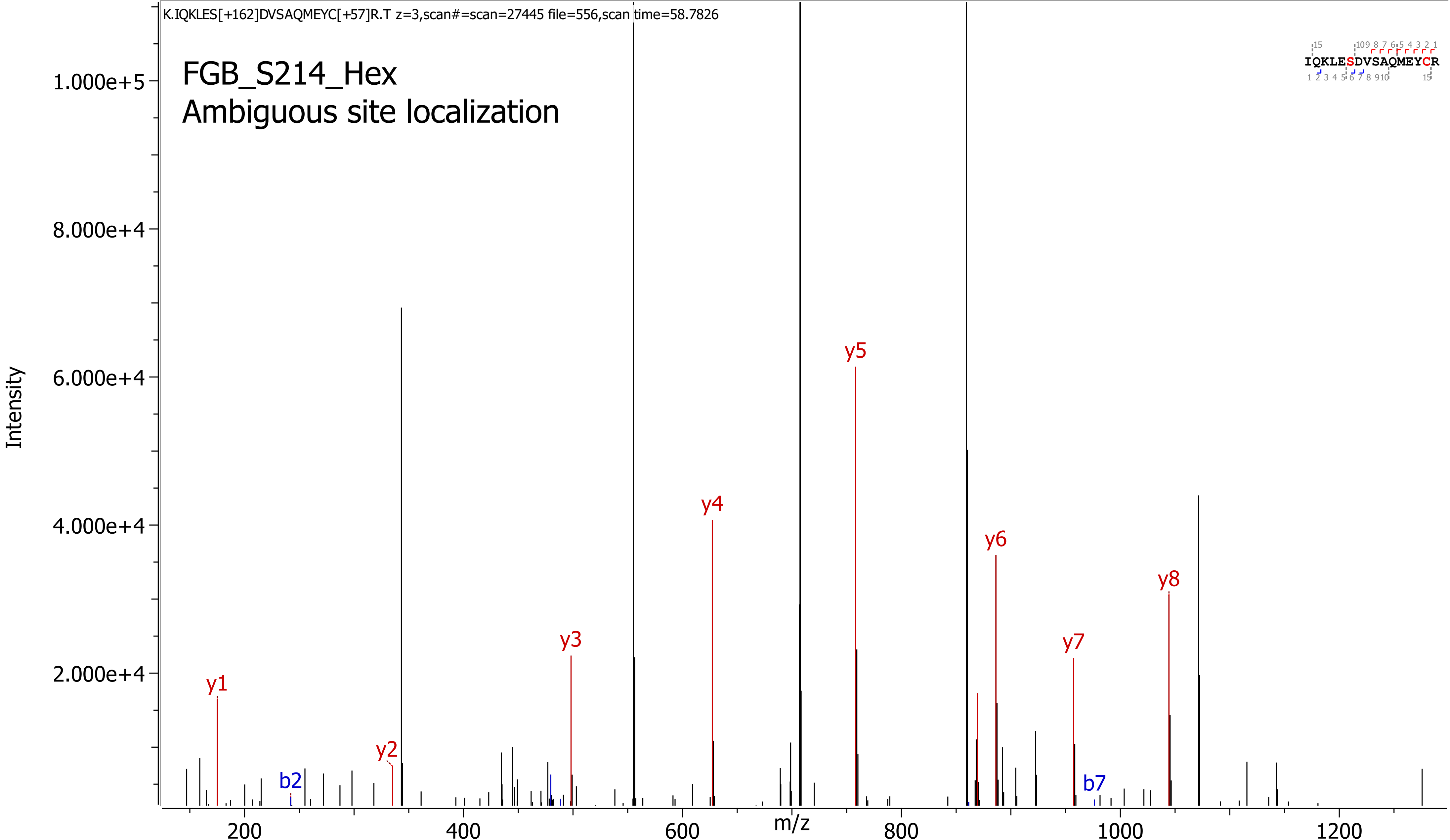

K.IQKLES[+162]DVSAQMEYC[+57]R.T z=3,scan#=scan=27445 file=556,scan time=58.7826
FGB_S214_Hex
Ambiguous site localization
IQKLESDVSAQMEYCR
Intensity
m/z
y1
y2
y3
y4
y5
y6
y7
y8
b2
b7

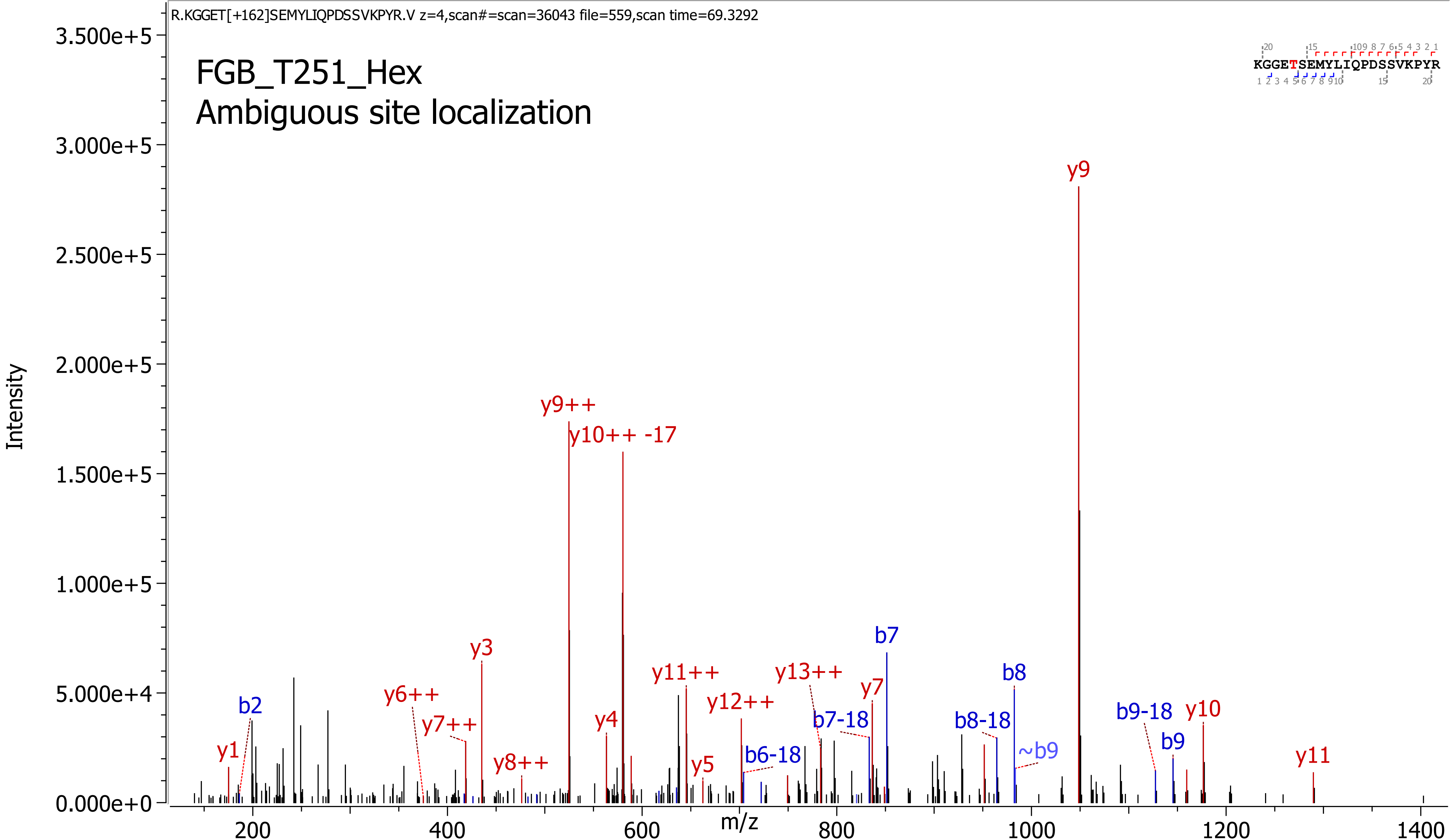
R.KGGET[+162]SEMYLIQPDSSVKPYR.V z=4,scan#=scan=36043 file=559,scan time=69.3292
FGB_T251_Hex
Ambiguous site localization
KGGETSEMYLIQPDSSVKPYR
Intensity
m/z
y9
y9++
y10++ -17
y3
y11++
y13++
y12++
y7
b7
b8
b7-18
b8-18
b6-18
~b9
b9-18
y10
b9
y11
y4
y5
y6++
y7++
y8++
b2
y1
3.500e+5
3.000e+5
2.500e+5
2.000e+5
1.500e+5
1.000e+5
5.000e+4
0.000e+0
200
400
600
800
1000
1200
1400

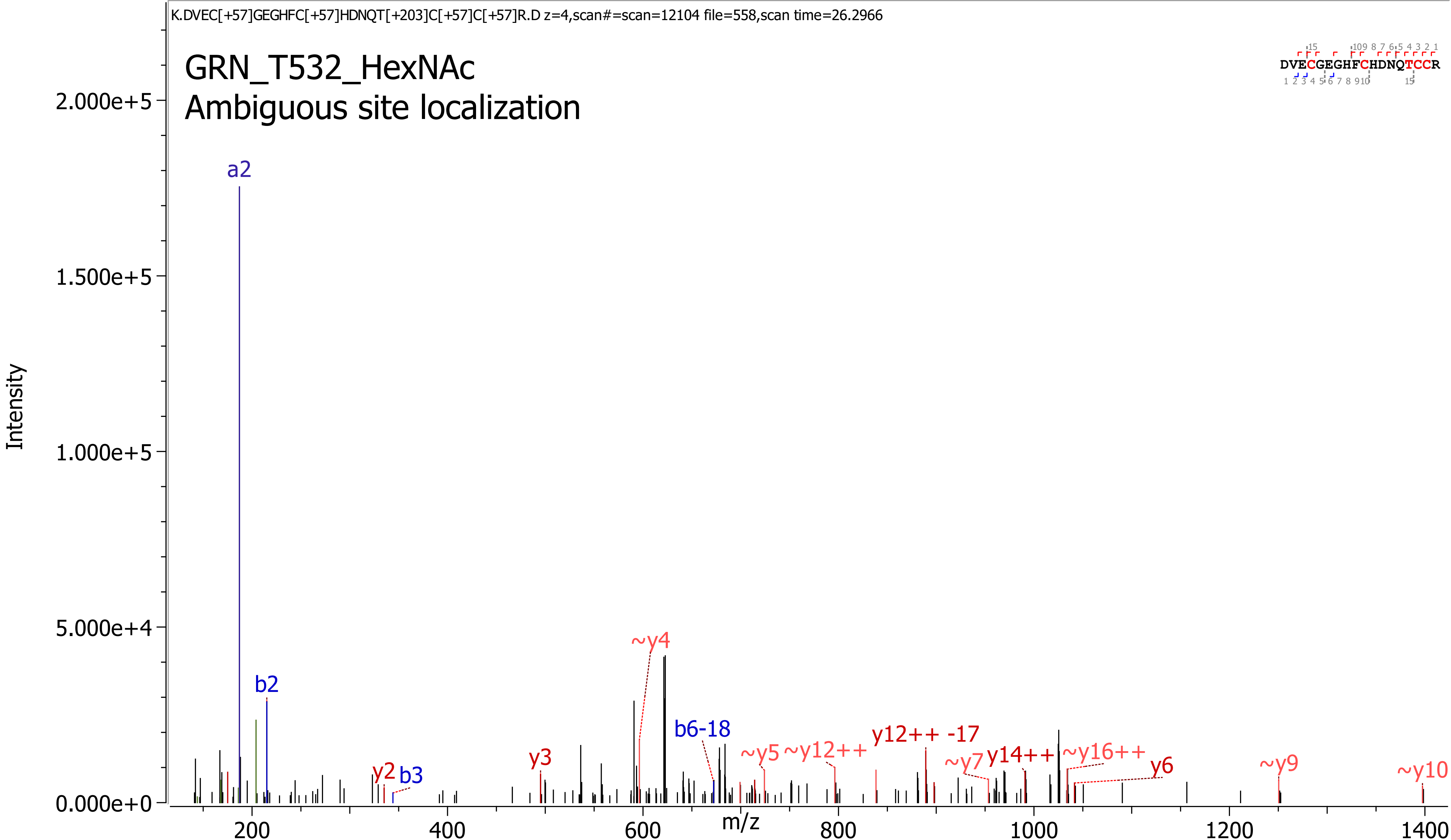

K.DVEC[+57]GEGHFC[+57]HDNQT[+203]C[+57]C[+57]R.D z=4,scan#=scan=12104 file=558,scan time=26.2966
GRN_T532_HexNAc
Ambiguous site localization
DVECGEGHFCHDNQTCCR
Intensity
m/z
a2
b2
b3
y2
y3
~y4
b6-18
~y5
~y12++
y12++ -17
~y7
y14++
~y16++
y6
~y9
~y10

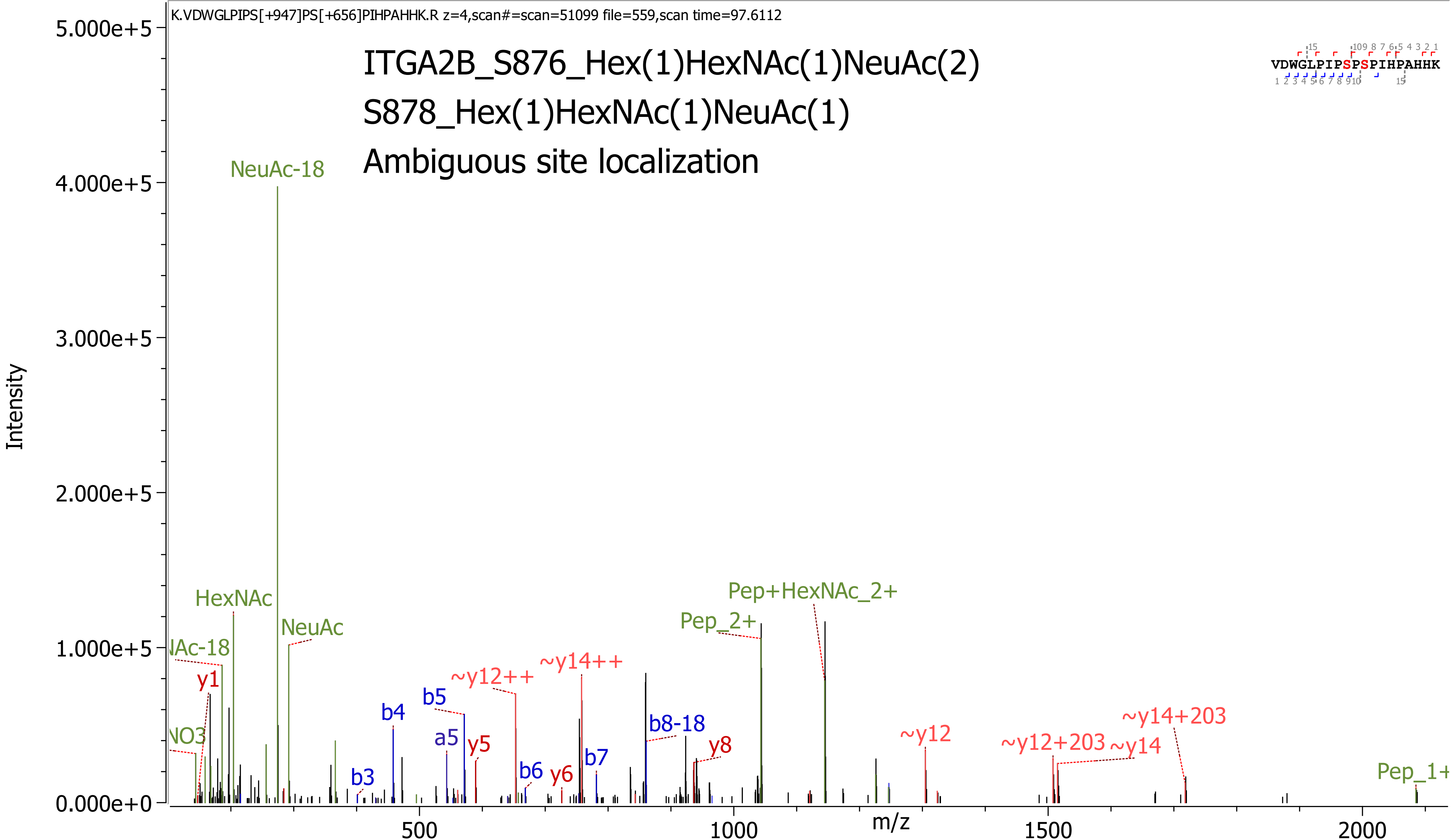
K.VDWGLPIPS[+947]PS[+656]PIHPAHHK.R z=4,scan#=scan=51099 file=559,scan time=97.6112
ITGA2B_S876_Hex(1)HexNAc(1)NeuAc(2)
S878_Hex(1)HexNAc(1)NeuAc(1)
Ambiguous site localization
VDWGLPIPSPSPIHPAHHK
Intensity
m/z
NeuAc-18
HexNAc
NeuAc
y1
b3
b4
b5
a5
y5
~y12++
~y14++
b6
y6
b7
b8-18
y8
Pep_2+
Pep+HexNAc_2+
~y12
~y12+203
~y14
~y14+203
5.000e+5
4.000e+5
3.000e+5
2.000e+5
1.000e+5
0.000e+0
500
1000
1500
2000

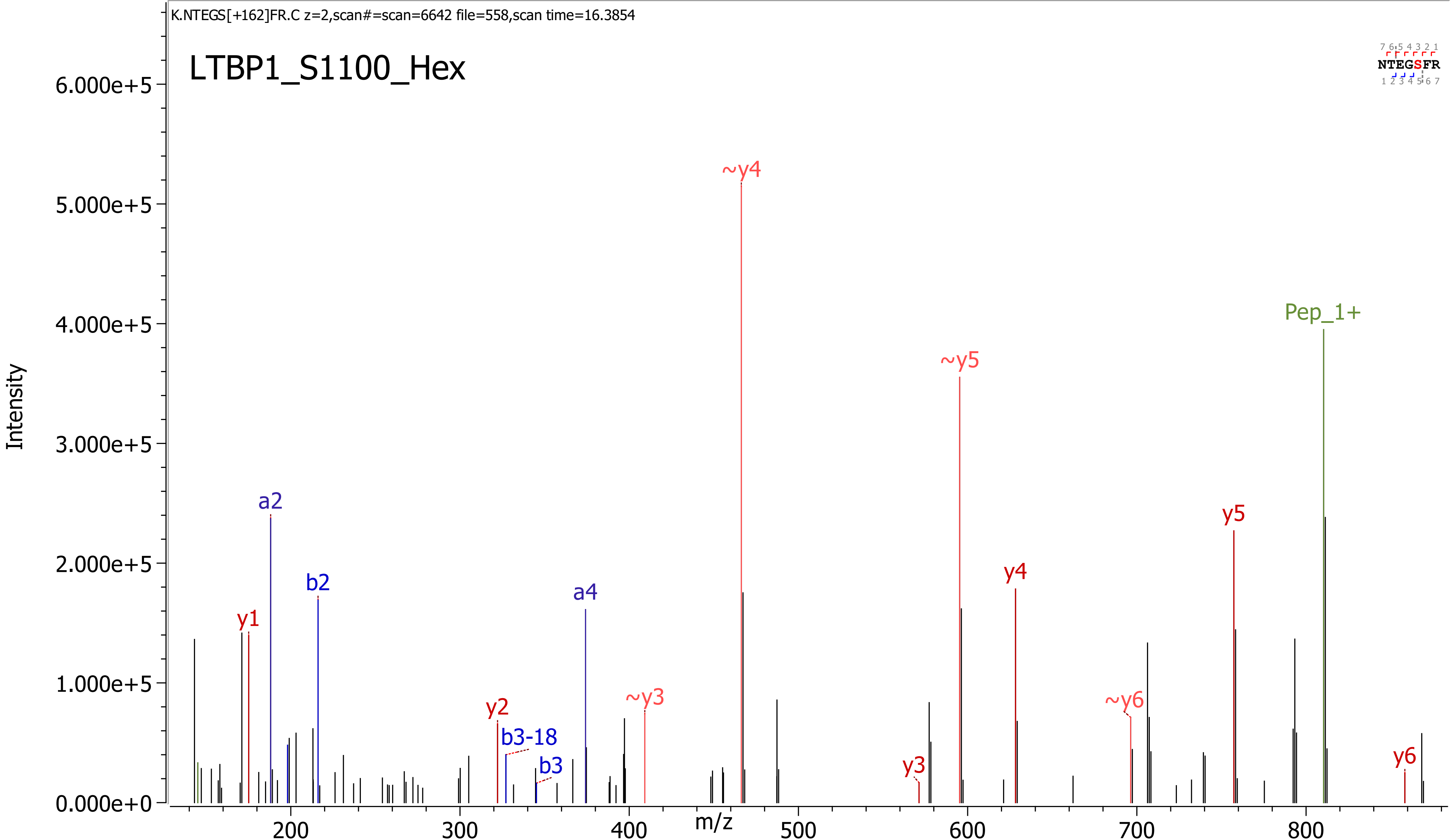
K.NTEGS[+162]FR.C z=2,scan#=scan=6642 file=558,scan time=16.3854
LTBP1_S1100_Hex
NTEGSFR
Intensity
m/z
~y4
Pep_1+
~y5
a2
y5
b2
a4
y4
y1
~y3
y2
b3-18
~y6
b3
y3
y6
6.000e+5
5.000e+5
4.000e+5
3.000e+5
2.000e+5
1.000e+5
0.000e+0
200
300
400
500
600
700
800

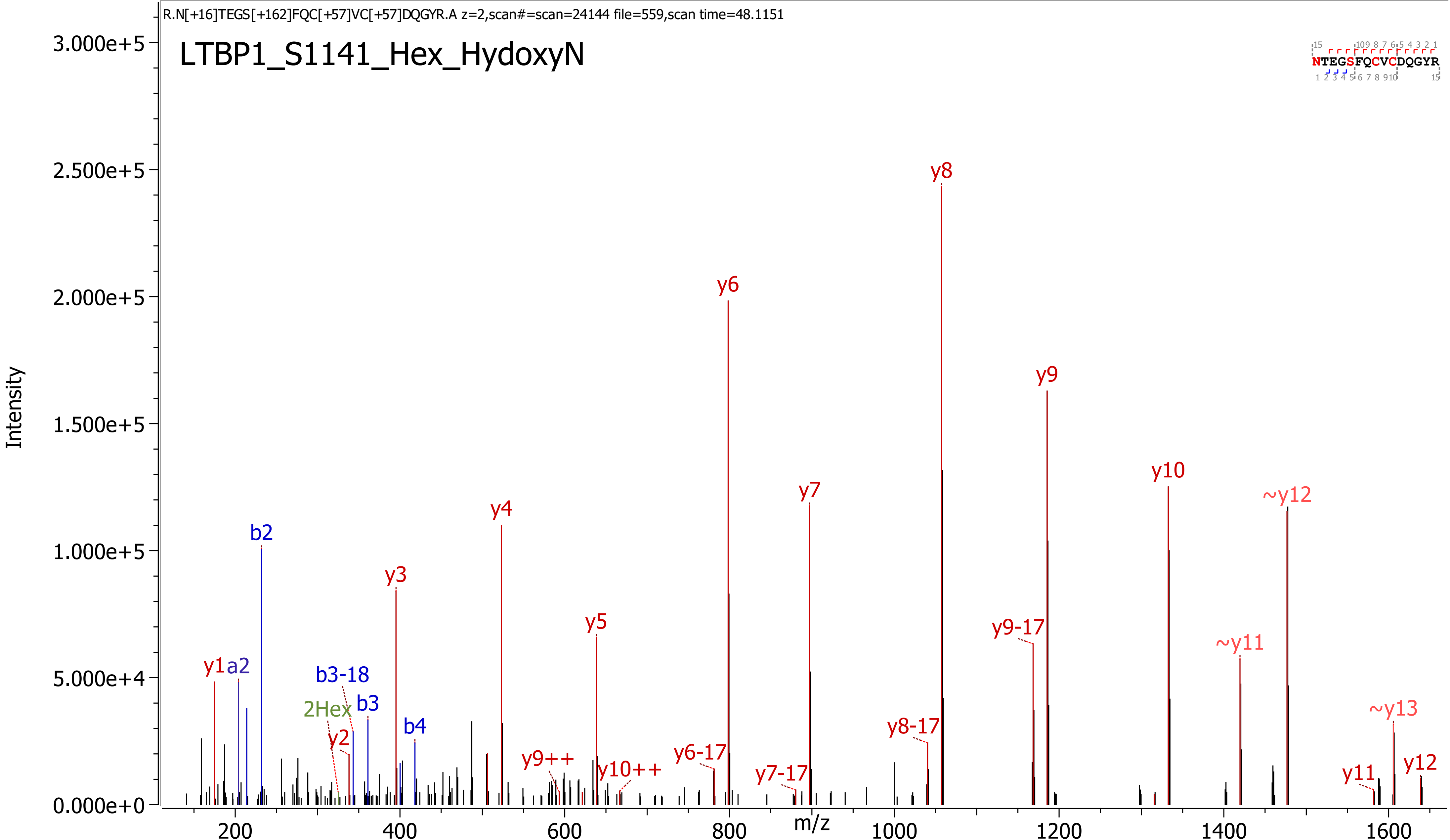
R.N[+16]TEGS[+162]FQC[+57]VC[+57]DQGYR.A z=2,scan#=scan=24144 file=559,scan time=48.1151
LTBP1_S1141_Hex_HydoxyN
NTEGSFQCVCDQGYR
Intensity
m/z
y1
a2
b2
2Hex
y2
b3-18
b3
y3
b4
y4
y9++
y5
y10++
y6-17
y6
y7-17
y7
y8-17
y8
y9-17
y9
y10
~y11
~y12
y11
~y13
y12

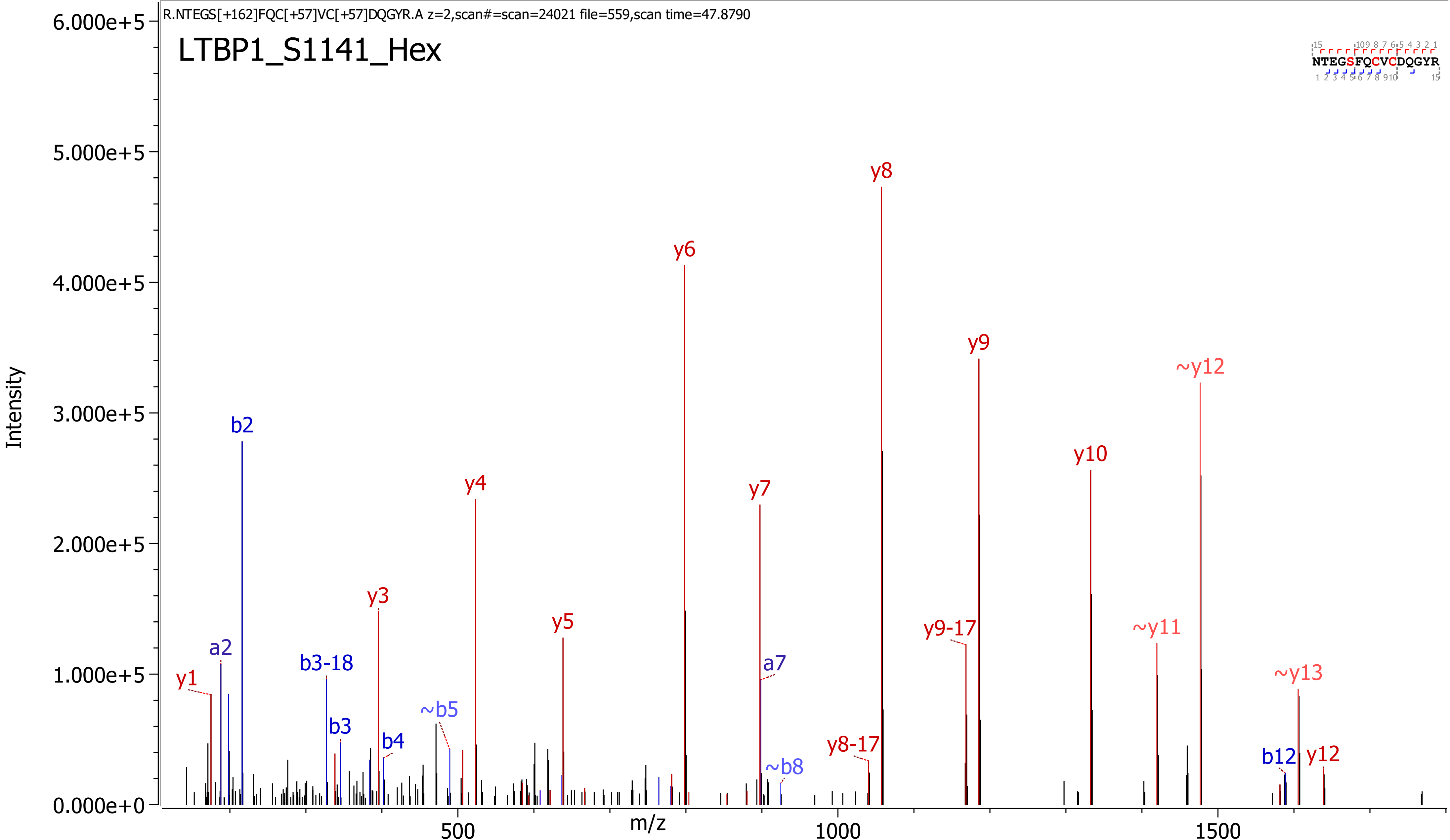
R.NTEGS[+162]FQC[+57]VC[+57]DQGYR.A z=2,scan#=scan=24021 file=559,scan time=47.8790
LTBP1_S1141_Hex
NTEGSFQCVCDQGYR
Intensity
m/z
6.000e+5
5.000e+5
4.000e+5
3.000e+5
2.000e+5
1.000e+5
0.000e+0
500
1000
1500
y1
a2
b2
b3-18
b3
y3
b4
~b5
y4
y5
y6
y7
a7
~b8
y8-17
y8
y9-17
y9
y10
~y11
~y12
b12
~y13
y12

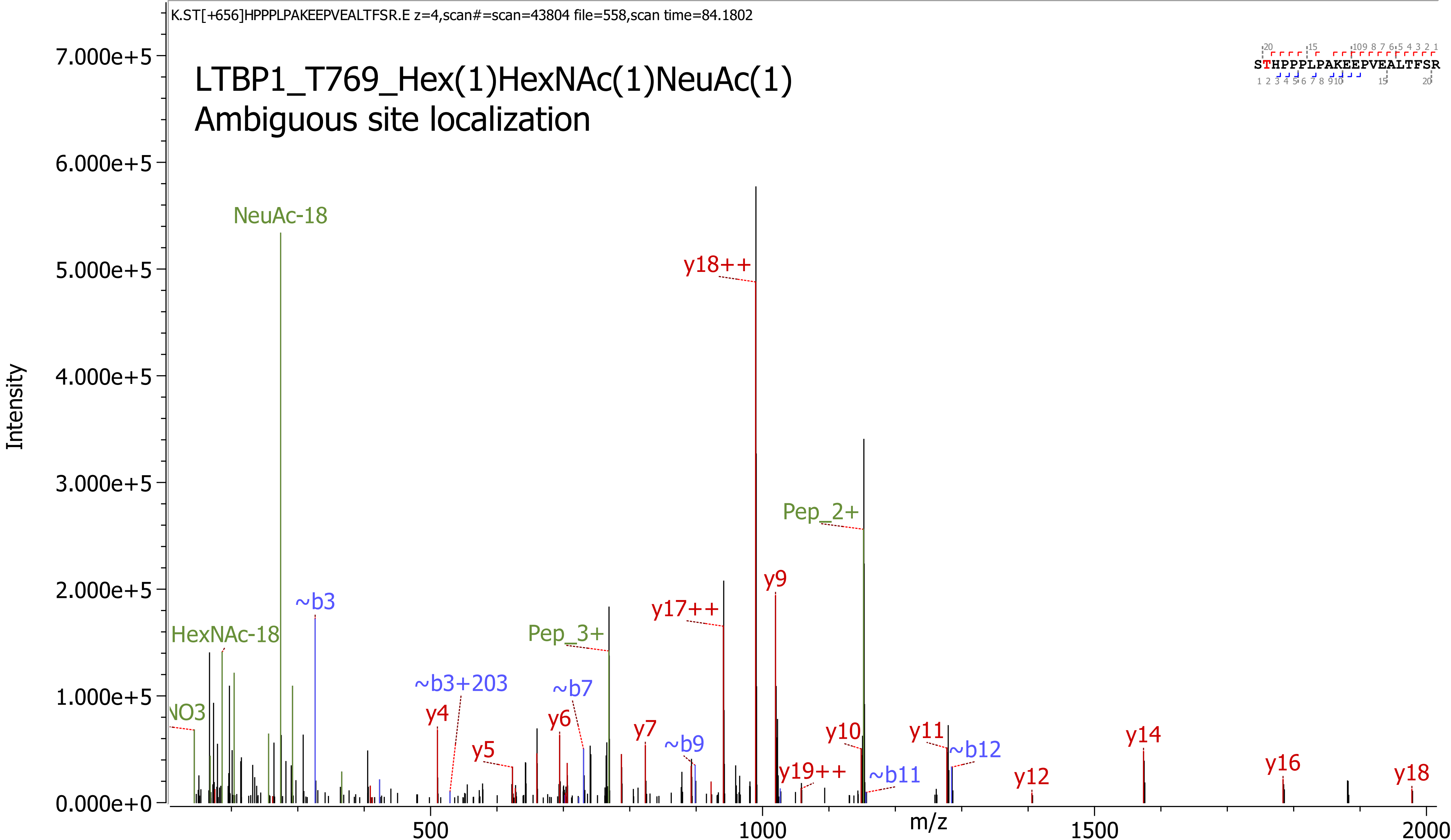
K.ST[+656]HPPPLPAKEEPVEALTFSR.E z=4,scan#=scan=43804 file=558,scan time=84.1802
LTBP1_T769_Hex(1)HexNAc(1)NeuAc(1)
Ambiguous site localization
STHPPPLPAKEEPVEALTFSR
Intensity
m/z
NeuAc-18
y18++
Pep_2+
y9
y17++
~b3
HexNAc-18
Pep_3+
~b3+203
~b7
y4
y6
y5
y7
~b9
y10
y11
~b12
y14
y19++
~b11
y12
y16
y18
NO3

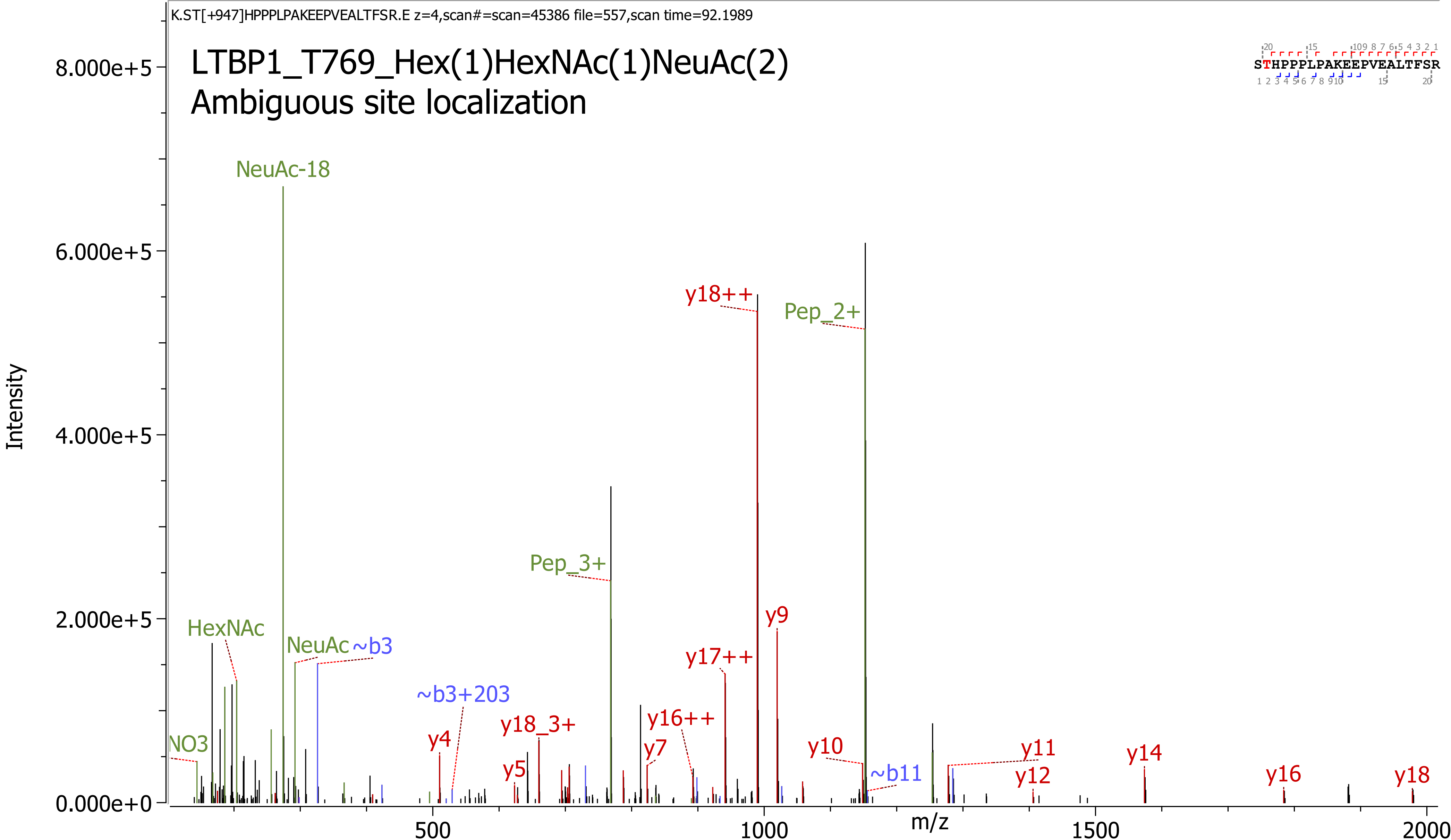

K.ST[+947]HPPPLPAKEEPVEALTFSR.E z=4,scan#=scan=45386 file=557,scan time=92.1989
LTBP1_T769_Hex(1)HexNAc(1)NeuAc(2)
Ambiguous site localization
STHPPPLPAKEEPVEALTFSR
Intensity
m/z
NeuAc-18
HexNAc
NeuAc
~b3
NO3
~b3+203
y4
y5
y18_3+
Pep_3+
y7
y16++
y17++
y18++
y9
Pep_2+
y10
~b11
y11
y12
y14
y16
y18

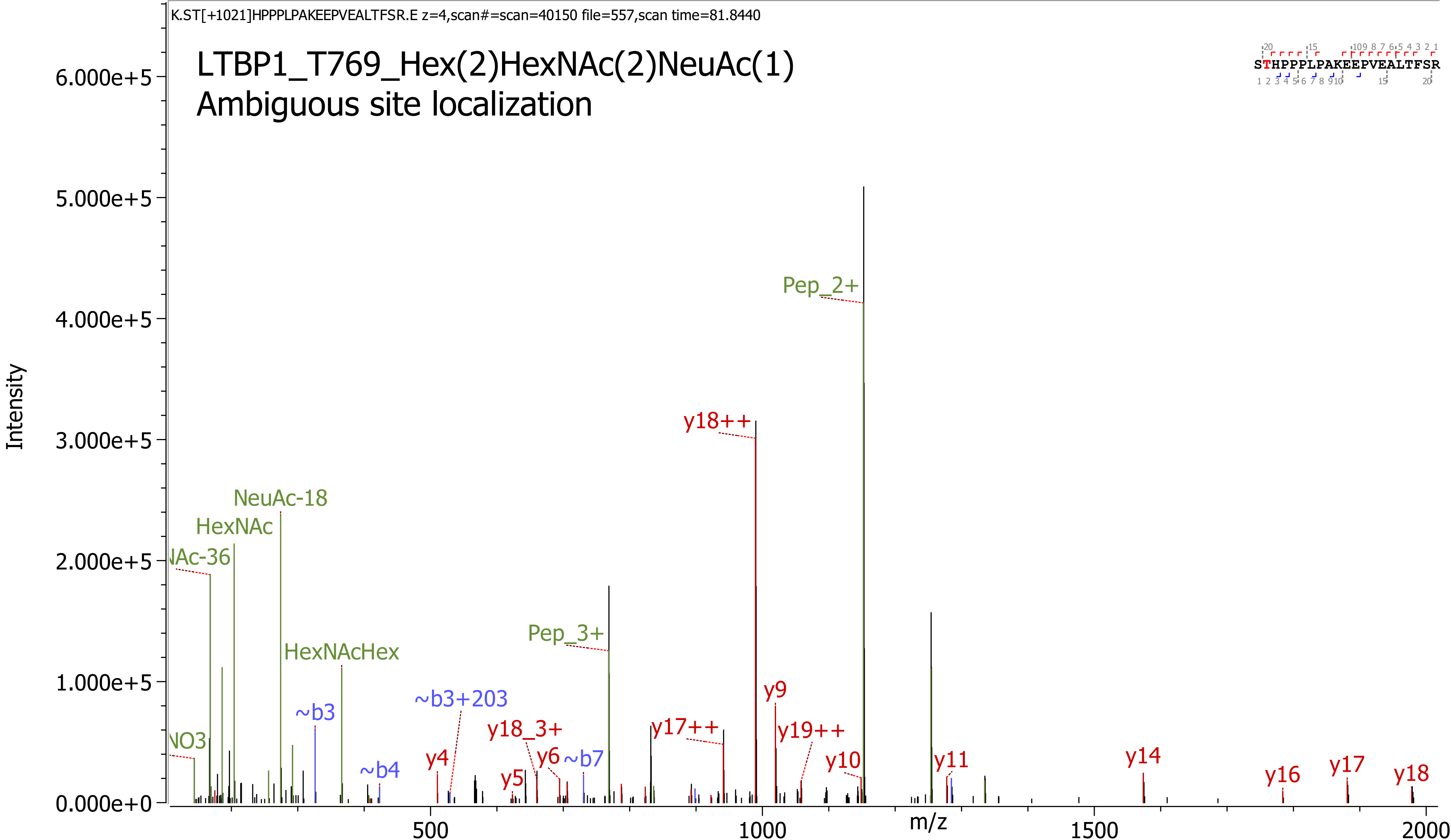
K.ST[+1021]HPPPLPAKEEPVEALTFSR.E z=4,scan#=scan=40150 file=557,scan time=81.8440
LTBP1_T769_Hex(2)HexNAc(2)NeuAc(1)
Ambiguous site localization
STHPPPLPAKEEPVEALTFSR
Intensity
m/z
Pep_2+
y18++
NeuAc-18
HexNAc
HexNAcHex
Pep_3+
~b3
~b3+203
~b4
y4
y5
y18_3+
y6
~b7
y17++
y9
y19++
y10
y11
y14
y16
y17
y18

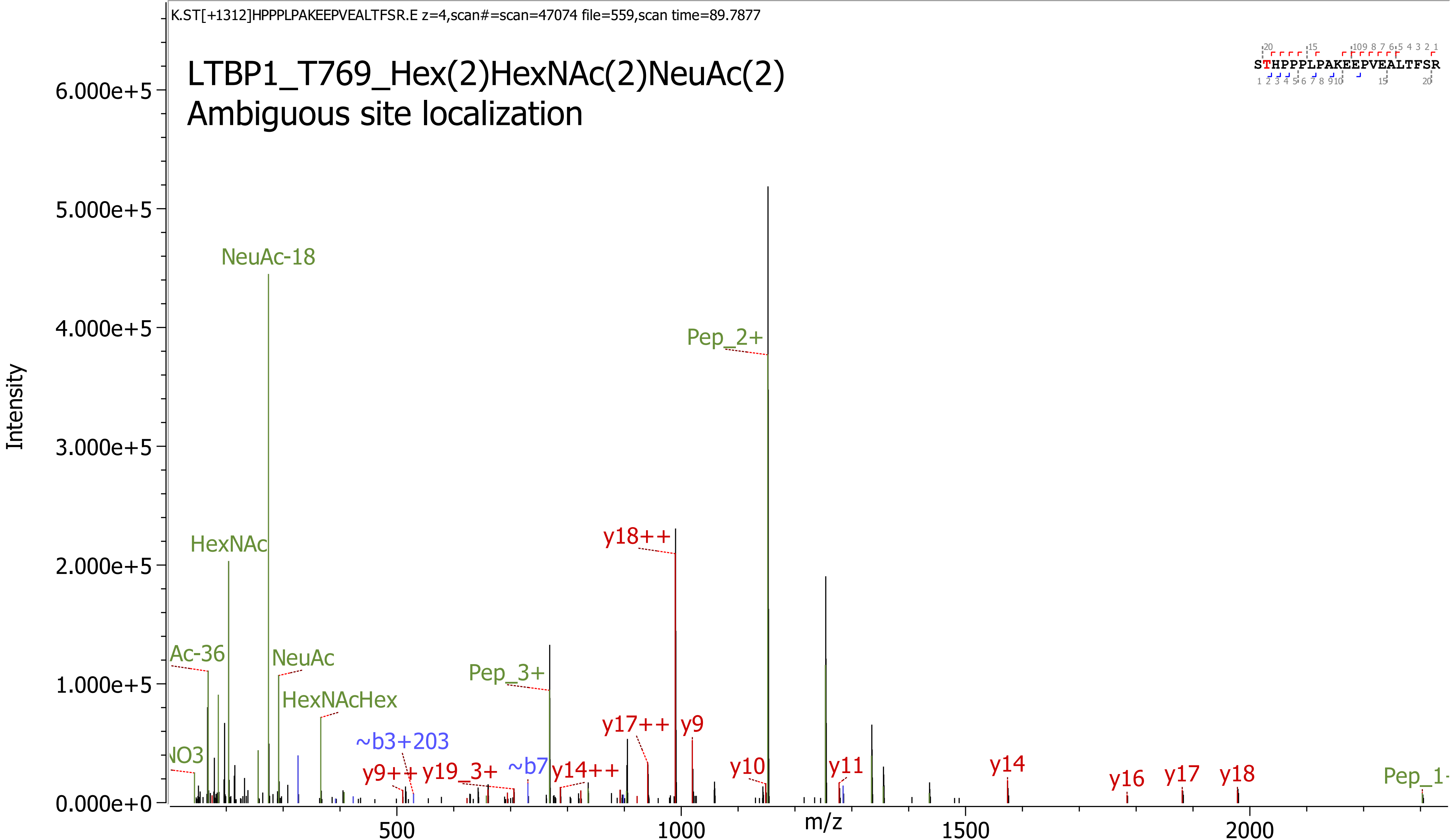

K.ST[+1312]HPPPLPAKEEPVEALTFSR.E z=4,scan#=scan=47074 file=559,scan time=89.7877
LTBP1_T769_Hex(2)HexNAc(2)NeuAc(2)
Ambiguous site localization
STHPPPLPAKEEPVEALTFSR
Intensity
m/z
NeuAc-18
HexNAc
Ac-36
NeuAc
HexNAcHex
NO3
~b3+203
y9++
y19_3+
~b7
y14++
Pep_3+
y18++
y17++
y9
y10
Pep_2+
y11
y14
y16
y17
y18
Pep_1

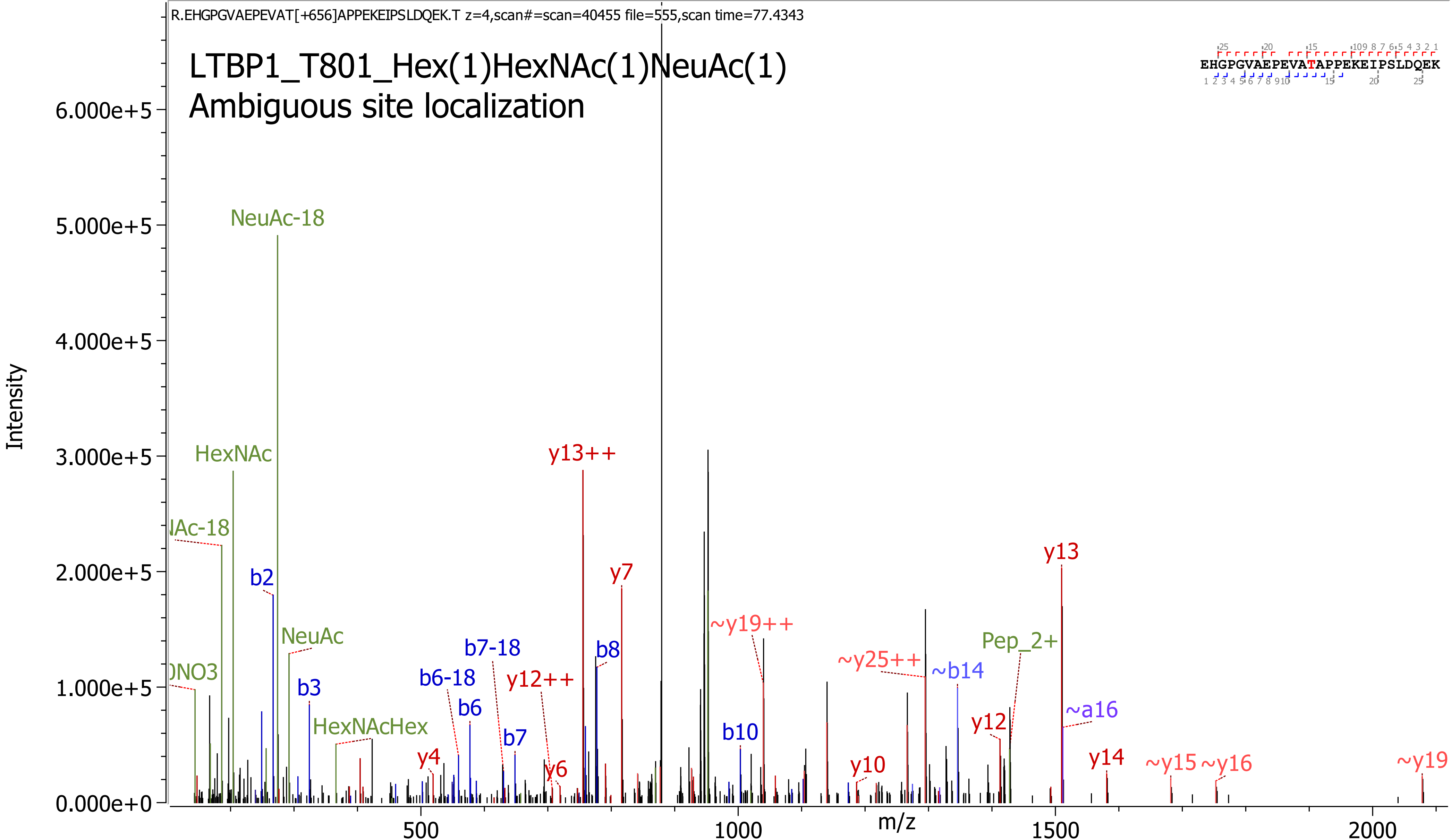
R.EHGPGVAEPEVAT[+656]APPEKEIPSLDQEK.T z=4,scan#=scan=40455 file=555,scan time=77.4343
LTBP1_T801_Hex(1)HexNAc(1)NeuAc(1)
Ambiguous site localization
EHGPGVAEPEVATAPPEKEIPSLDQEK
Intensity
m/z
NeuAc-18
HexNAc
NeuAc
HexNAcHex
Pep_2+
b2
b3
b6-18
b6
b7-18
b7
b8
b10
~b14
~a16
y4
y6
y7
y10
y12
y12++
y13
y13++
y14
~y15
~y16
~y19
~y19++
~y25++

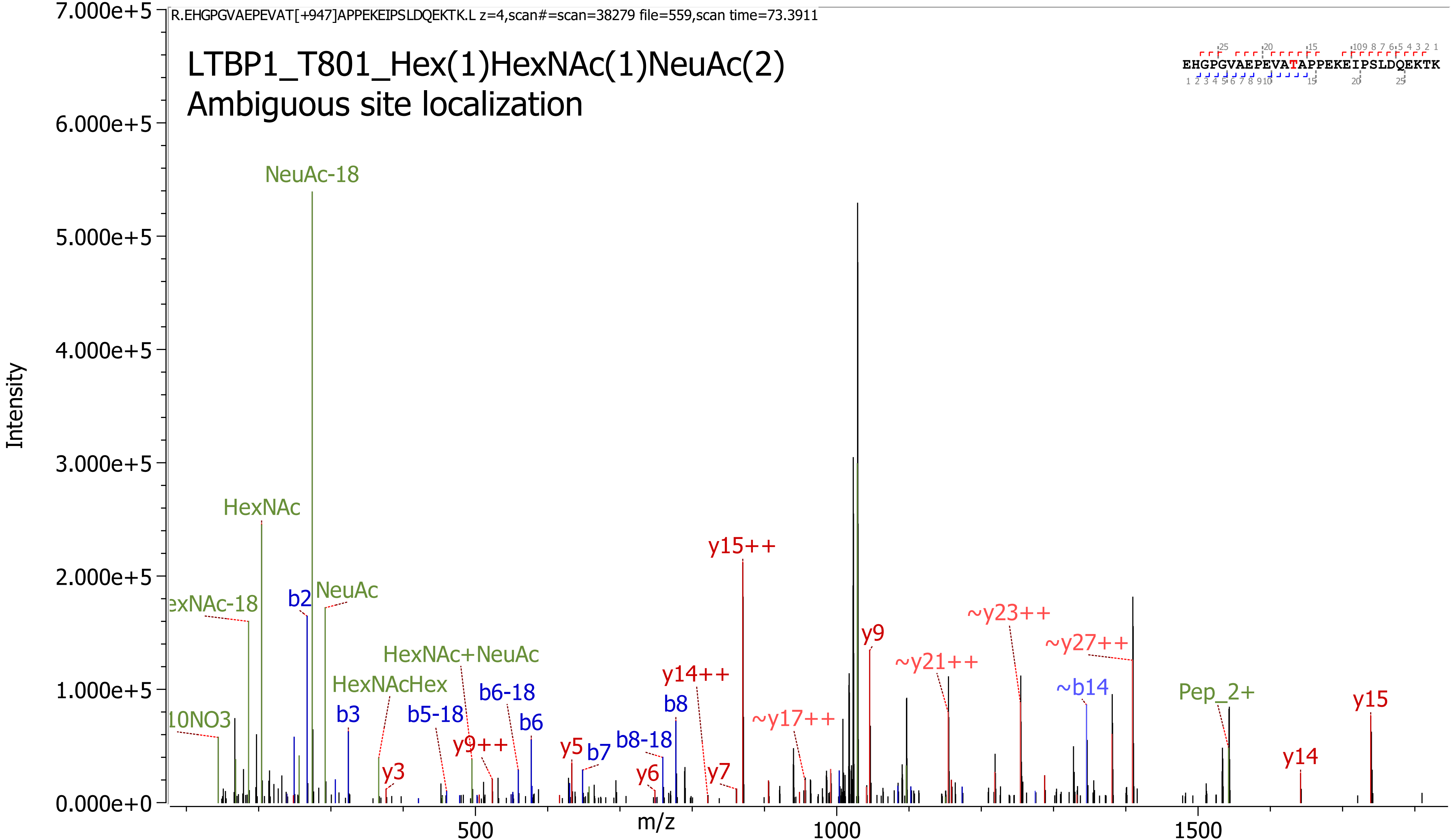

R.EHGPGVAEPEVAT[+947]APPEKEIPSLDQEKTK.L z=4,scan#=scan=38279 file=559,scan time=73.3911
LTBP1_T801_Hex(1)HexNAc(1)NeuAc(2)
Ambiguous site localization
EHGPGVAEPEVATAPPEKEIPSLDQEKTK
Intensity
m/z
NeuAc-18
HexNAc
NeuAc
HexNAc+NeuAc
HexNAcHex
y15++
y14++
y9
~y21++
~y23++
~y27++
~b14
Pep_2+
y15
y14
~y17++
b2
b3
b5-18
b6-18
b6
b7
b8
b8-18
y3
y5
y6
y7
y9++

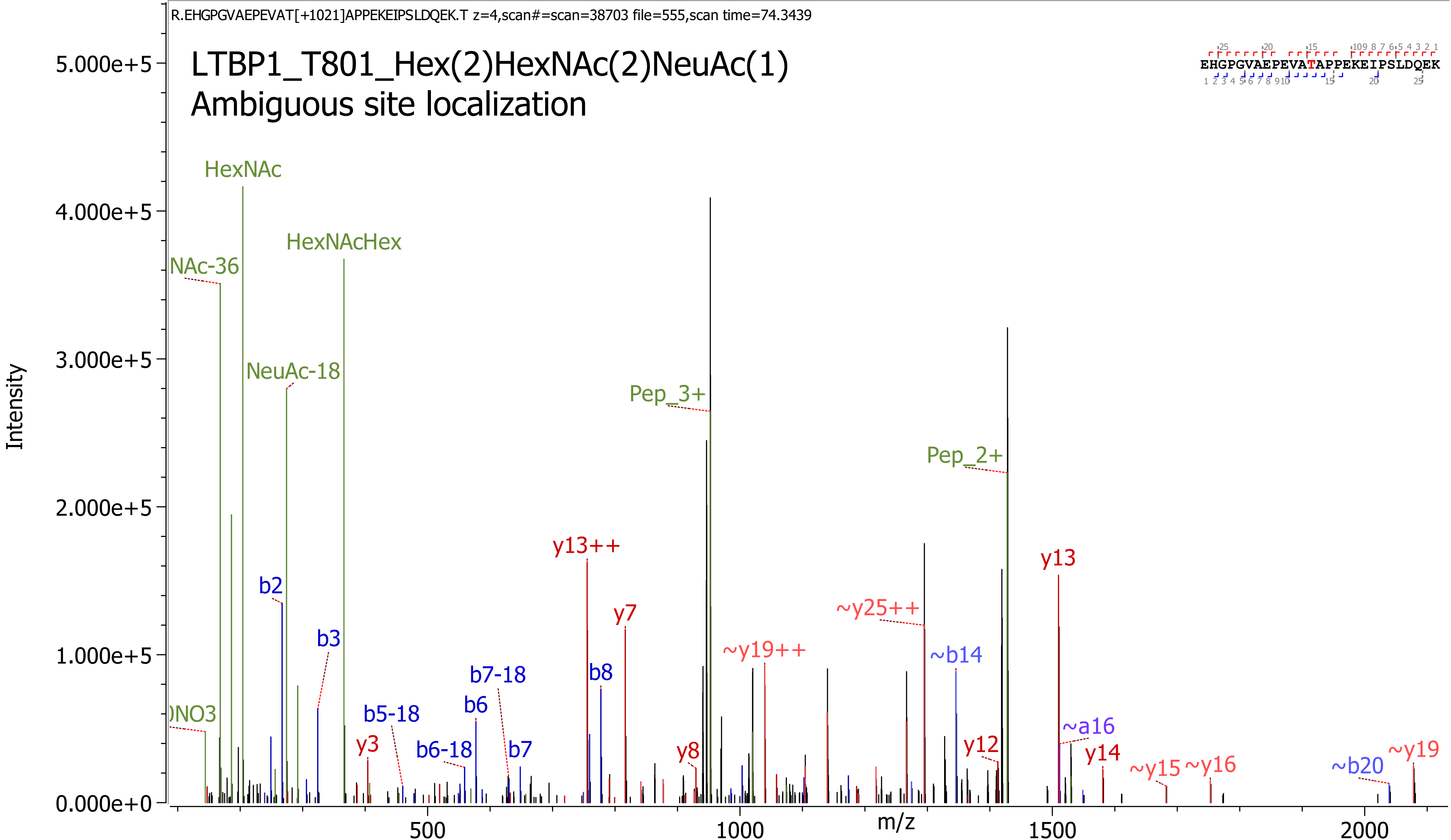
R.EHGPGVAEPEVAT[+1021]APPEKEIPSLDQEK.T z=4,scan#=scan=38703 file=555,scan time=74.3439
LTBP1_T801_Hex(2)HexNAc(2)NeuAc(1)
Ambiguous site localization
EHGPGVAEPEVATAPPEKEIPSLDQEK
Intensity
m/z
HexNAc
HexNAcHex
NAc-36
NeuAc-18
Pep_3+
Pep_2+
b2
b3
b5-18
b6-18
b6
b7-18
b7
b8
y3
y7
y8
y12
y13
y13++
y14
~y19++
~y25++
~b14
~a16
~y15
~y16
~b20
~y19
5.000e+5
4.000e+5
3.000e+5
2.000e+5
1.000e+5
0.000e+0
500
1000
1500
2000

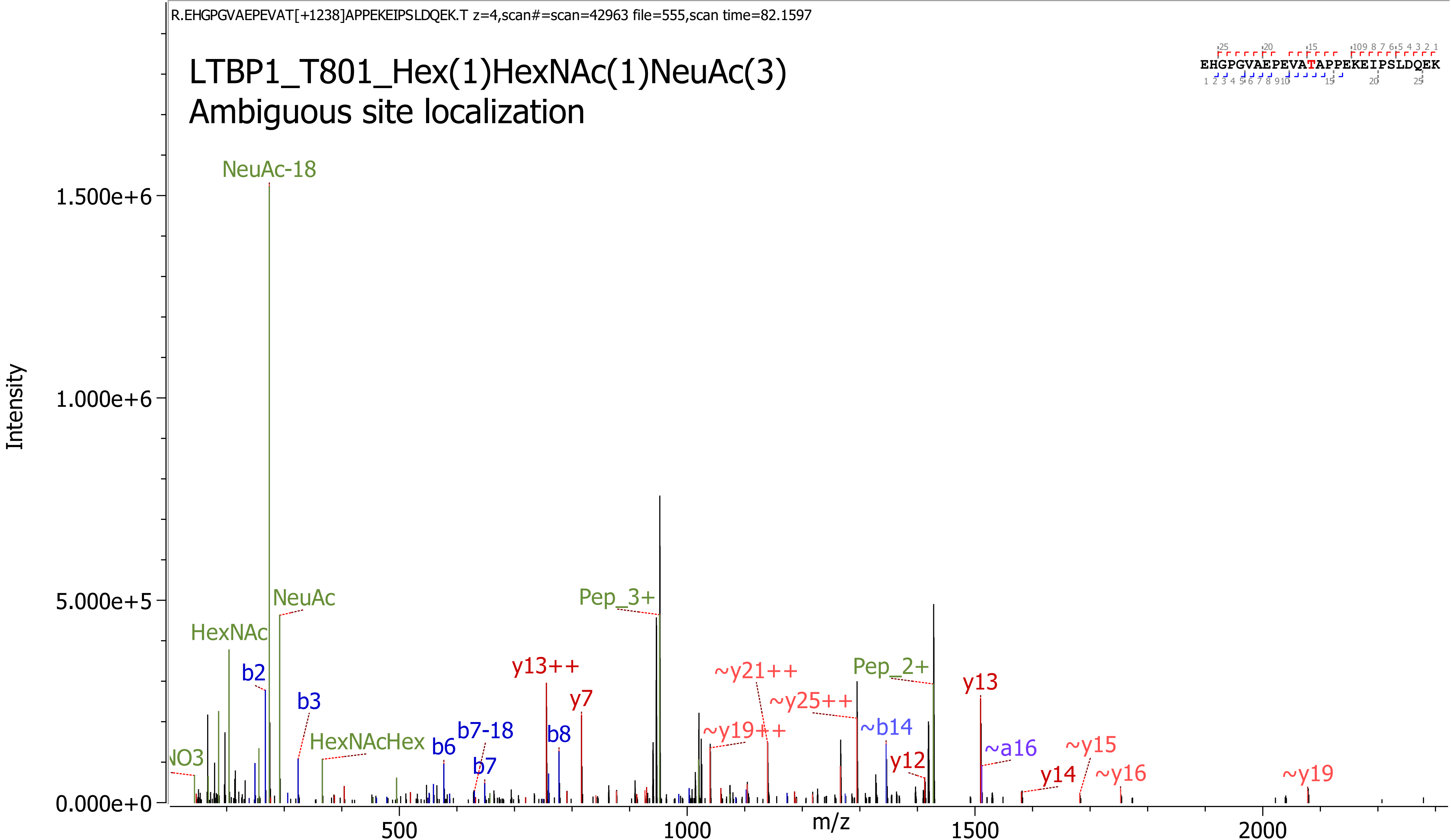
R.EHGPGVAEPEVAT[+1238]APPEKEIPSLDQEK.T z=4,scan#=scan=42963 file=555,scan time=82.1597
LTBP1_T801_Hex(1)HexNAc(1)NeuAc(3)
Ambiguous site localization
EHGPGVAEPEVATAPPEKEIPSLDQEK
Intensity
m/z
NeuAc-18
NeuAc
HexNAc
b2
b3
HexNAcHex
b6
b7-18
b7
b8
y13++
y7
Pep_3+
~y21++
~y19++
~y25++
Pep_2+
~b14
y12
y13
~a16
y14
~y15
~y16
~y19
1.500e+6
1.000e+6
5.000e+5
0.000e+0
500
1000
1500
2000

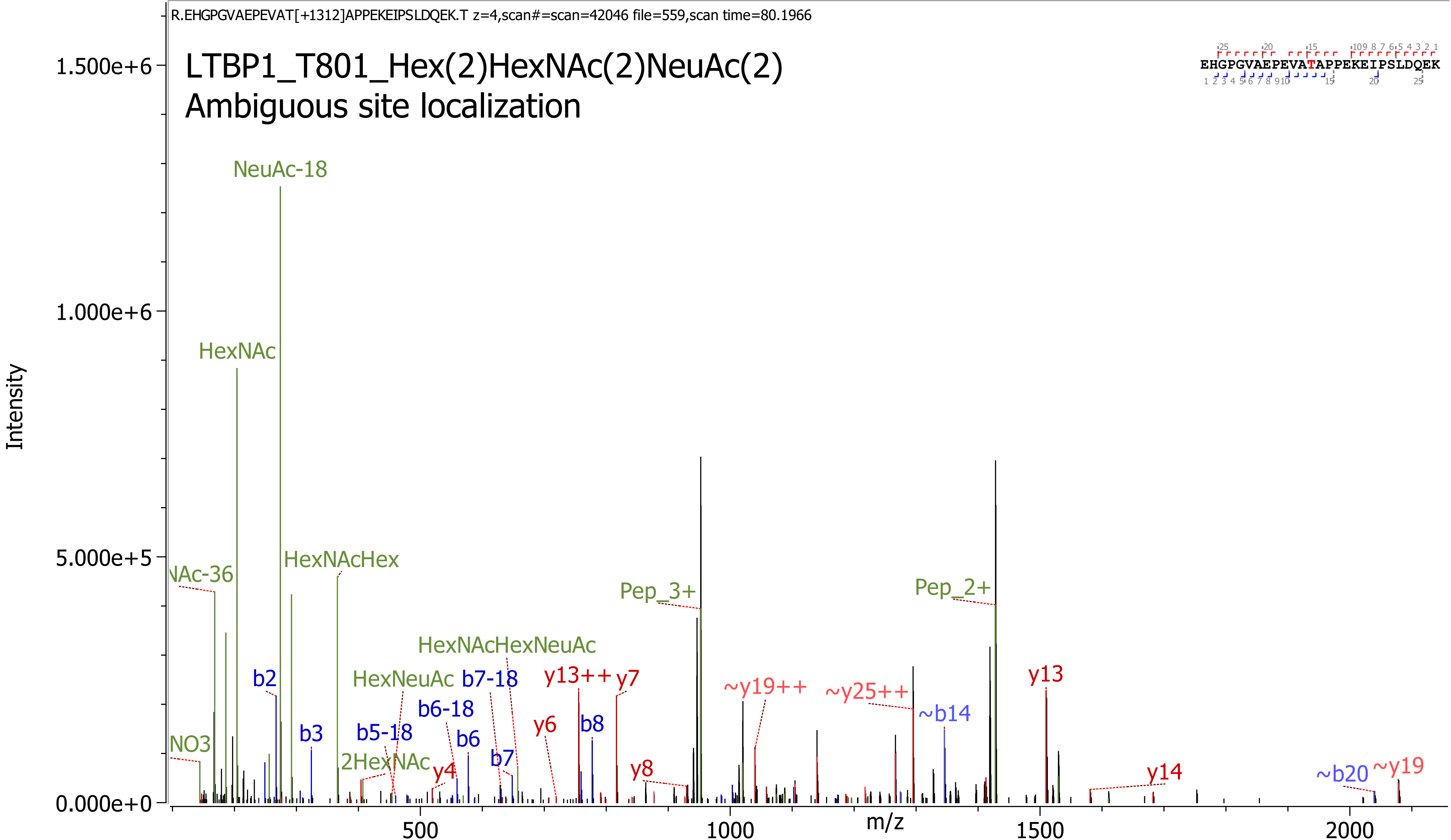

R.EHGPGVAEPEVAT[+1312]APPEKEIPSLDQEK.T z=4,scan#=scan=42046 file=559,scan time=80.1966
LTBP1_T801_Hex(2)HexNAc(2)NeuAc(2)
Ambiguous site localization
EHGPGVAEPEVATAPPEKEIPSLDQEK
NeuAc-18
HexNAc
HexNAcHex
Pep_3+
Pep_2+
HexNAcHexNeuAc
HexNeuAc
2HexNAc
b2
b3
b5-18
b6-18
b6
b7
b7-18
b8
y4
y6
y7
y8
y13++
~y19++
~y25++
~b14
y13
y14
~b20
~y19
Intensity
m/z

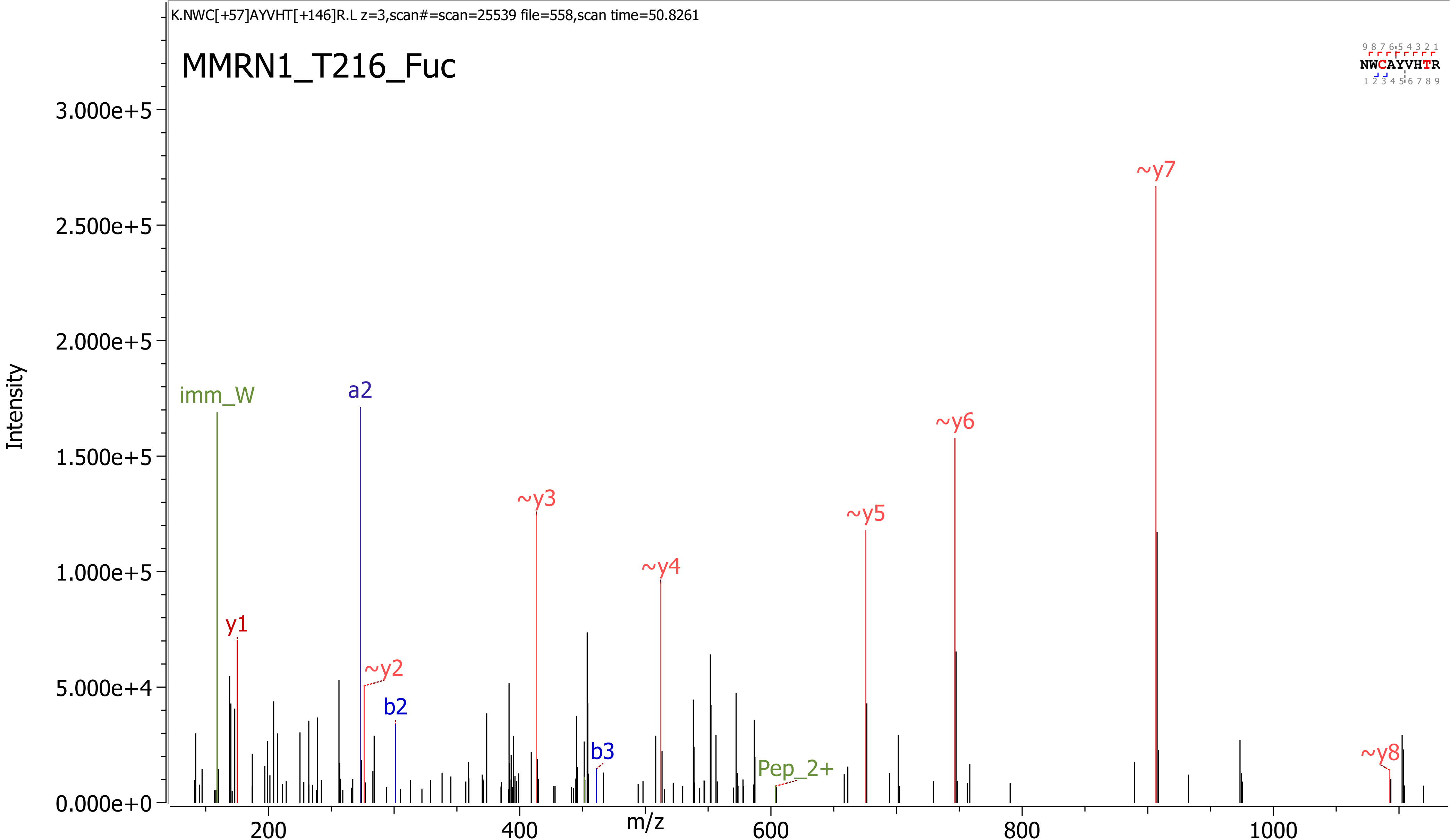
K.NWC[+57]AYVHT[+146]R.L z=3,scan#=scan=25539 file=558,scan time=50.8261
MMRN1_T216_Fuc
NWCAYVHTR
Intensity
m/z
imm_W
a2
y1
~y2
b2
~y3
b3
~y4
Pep_2+
~y5
~y6
~y7
~y8
0.000e+0
5.000e+4
1.000e+5
1.500e+5
2.000e+5
2.500e+5
3.000e+5
200
400
600
800
1000

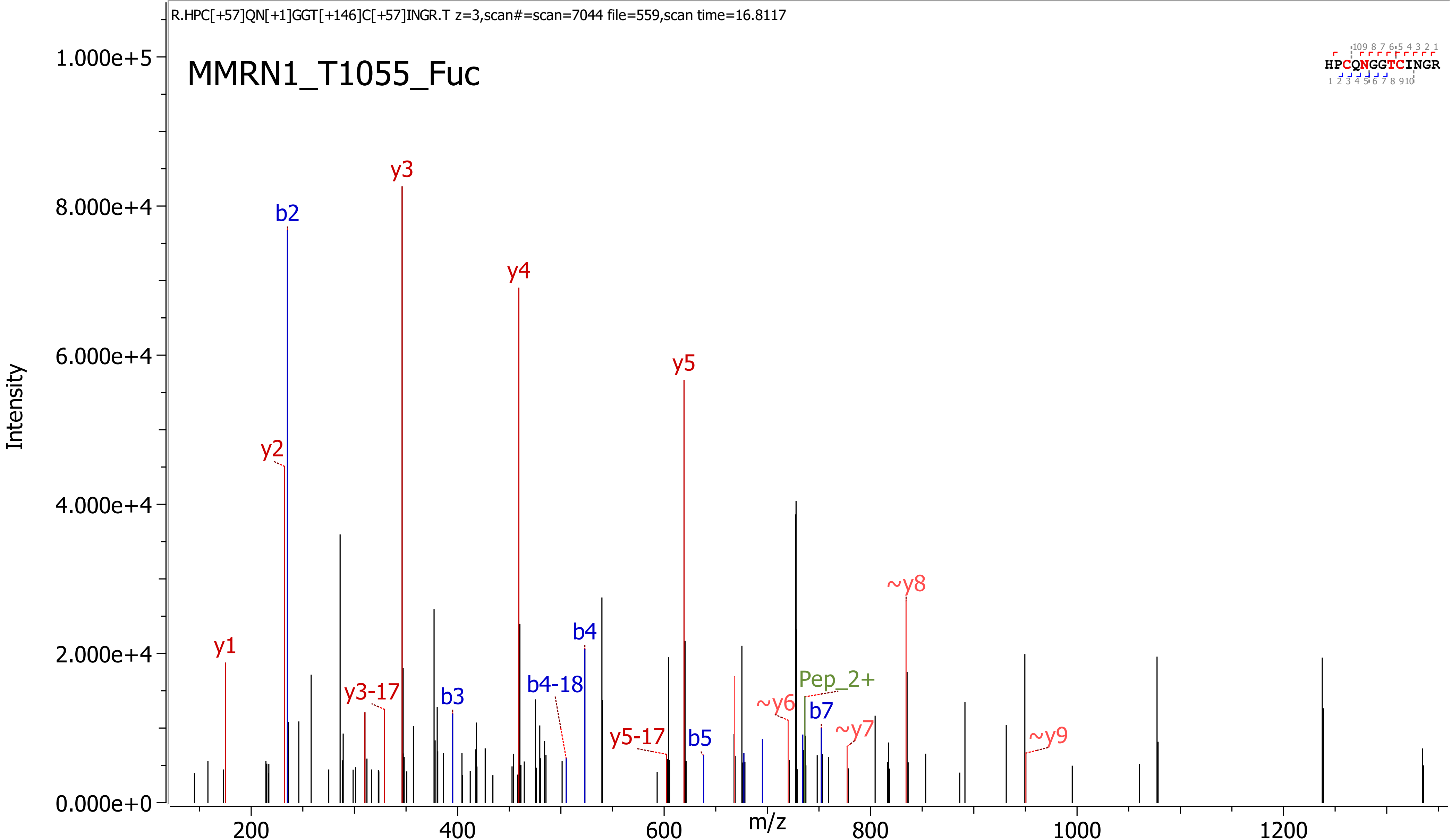
R.HPC[+57]QN[+1]GGT[+146]C[+57]INGR.T z=3,scan#=scan=7044 file=559,scan time=16.8117
MMRN1_T1055_Fuc
HPCQNGGTCINGR
Intensity
m/z
y1
y2
b2
y3
y3-17
b3
y4
b4-18
b4
y5-17
y5
b5
~y6
Pep_2+
b7
~y7
~y8
~y9

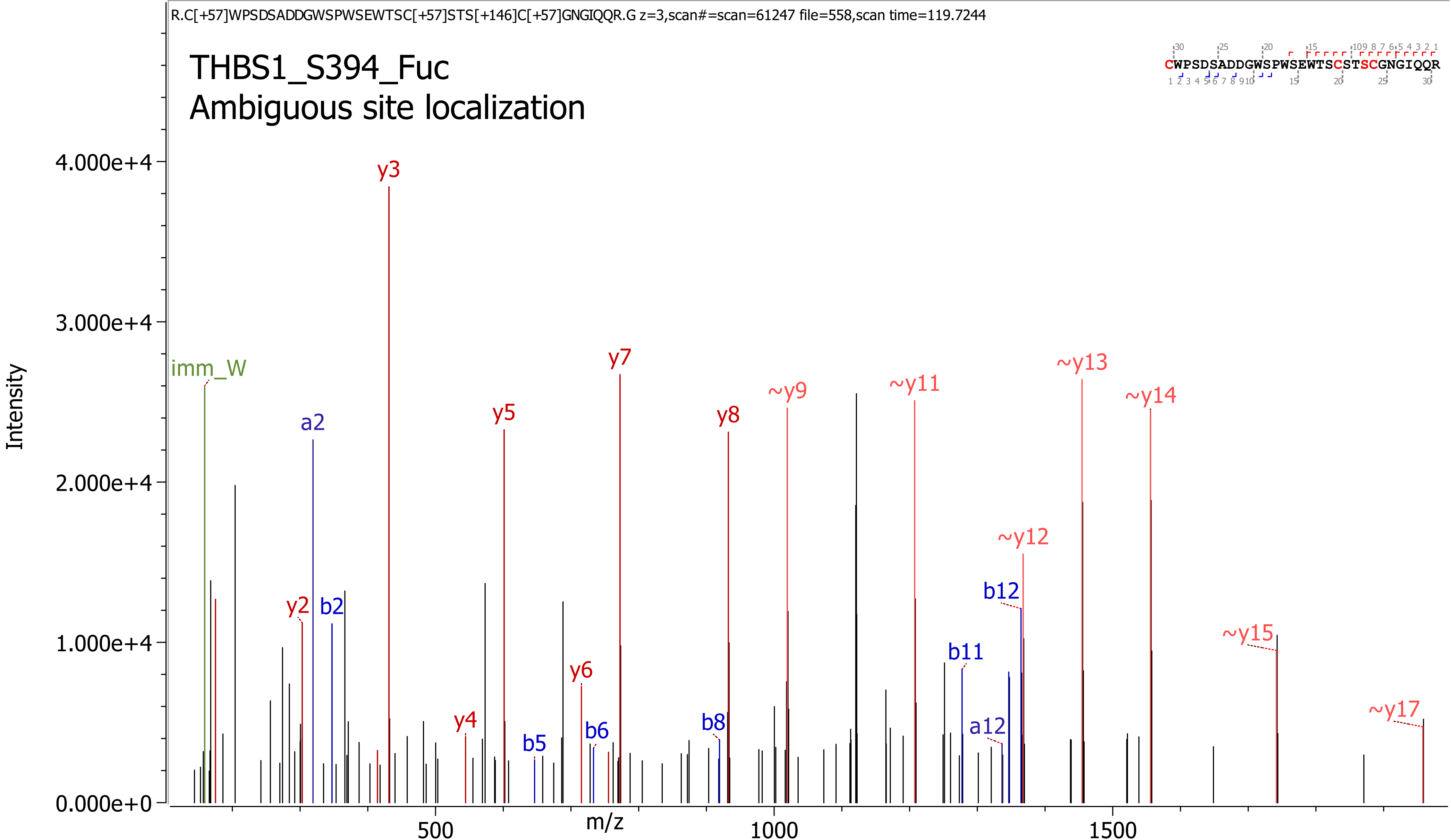
R.C[+57]WPSDSADDGWSPWSEWTSC[+57]STS[+146]C[+57]GNGIQQR.G z=3,scan#=scan=61247 file=558,scan time=119.7244
THBS1_S394_Fuc
Ambiguous site localization
CWPSDSADDGWSPWSEWTSCSTSCGNGIQQR
Intensity
m/z
imm_W
a2
y2
b2
y3
y4
y5
b5
y6
b6
y7
b8
y8
~y9
~y11
b11
a12
b12
~y12
~y13
~y14
~y15
~y17
4.000e+4
3.000e+4
2.000e+4
1.000e+4
0.000e+0
500
1000
1500

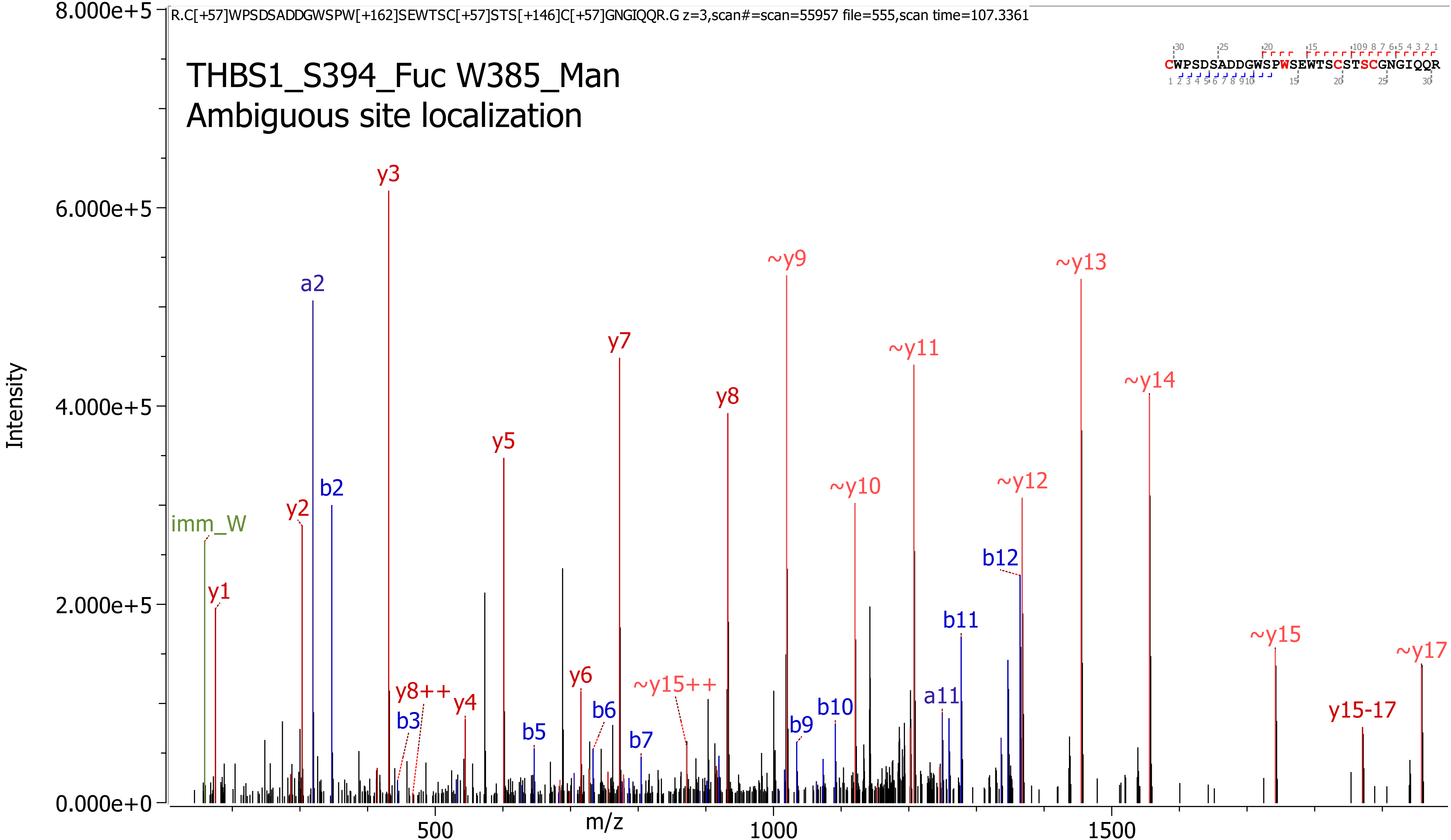

R.C[+57]WPSDSADDGWSPW[+162]SEWTSC[+57]STS[+146]C[+57]GNGIQQR.G z=3,scan#=scan=55957 file=555,scan time=107.3361
THBS1_S394_Fuc W385_Man
Ambiguous site localization
CWPSDSADDGWSPWSEWTSCSTSCGNGIQQR
Intensity
m/z
8.000e+5
6.000e+5
4.000e+5
2.000e+5
0.000e+0
500
1000
1500
imm_W
y1
y2
a2
b2
b3
y3
y8++
y4
y5
b5
y6
b6
y7
b7
~y15++
y8
~y9
b9
b10
~y10
~y11
a11
b11
b12
~y12
~y13
~y14
~y15
y15-17
~y17

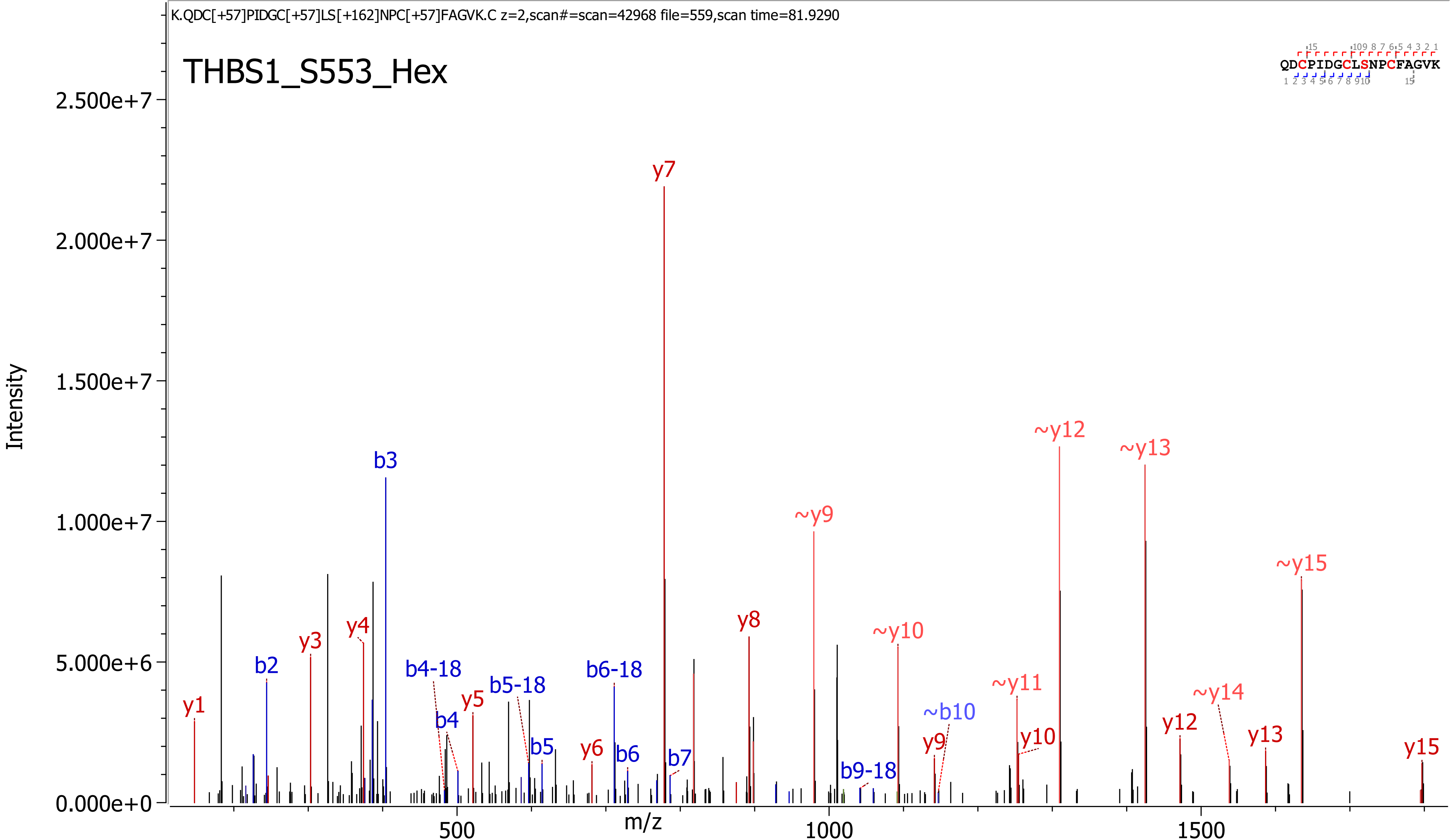
K.QDC[+57]PIDGC[+57]LS[+162]NPC[+57]FAGVK.C z=2,scan#=scan=42968 file=559,scan time=81.9290
THBS1_S553_Hex
QDCPIDGCLSNPCFAGVK
Intensity
m/z
y7
b3
~y12
~y13
~y9
~y15
y8
~y10
y4
y3
b2
b4-18
b6-18
b5-18
y5
b4
~y11
~y14
~b10
y1
y12
y10
y13
y15
b5
y6
b6
b7
y9
b9-18
2.500e+7
2.000e+7
1.500e+7
1.000e+7
5.000e+6
0.000e+0
500
1000
1500

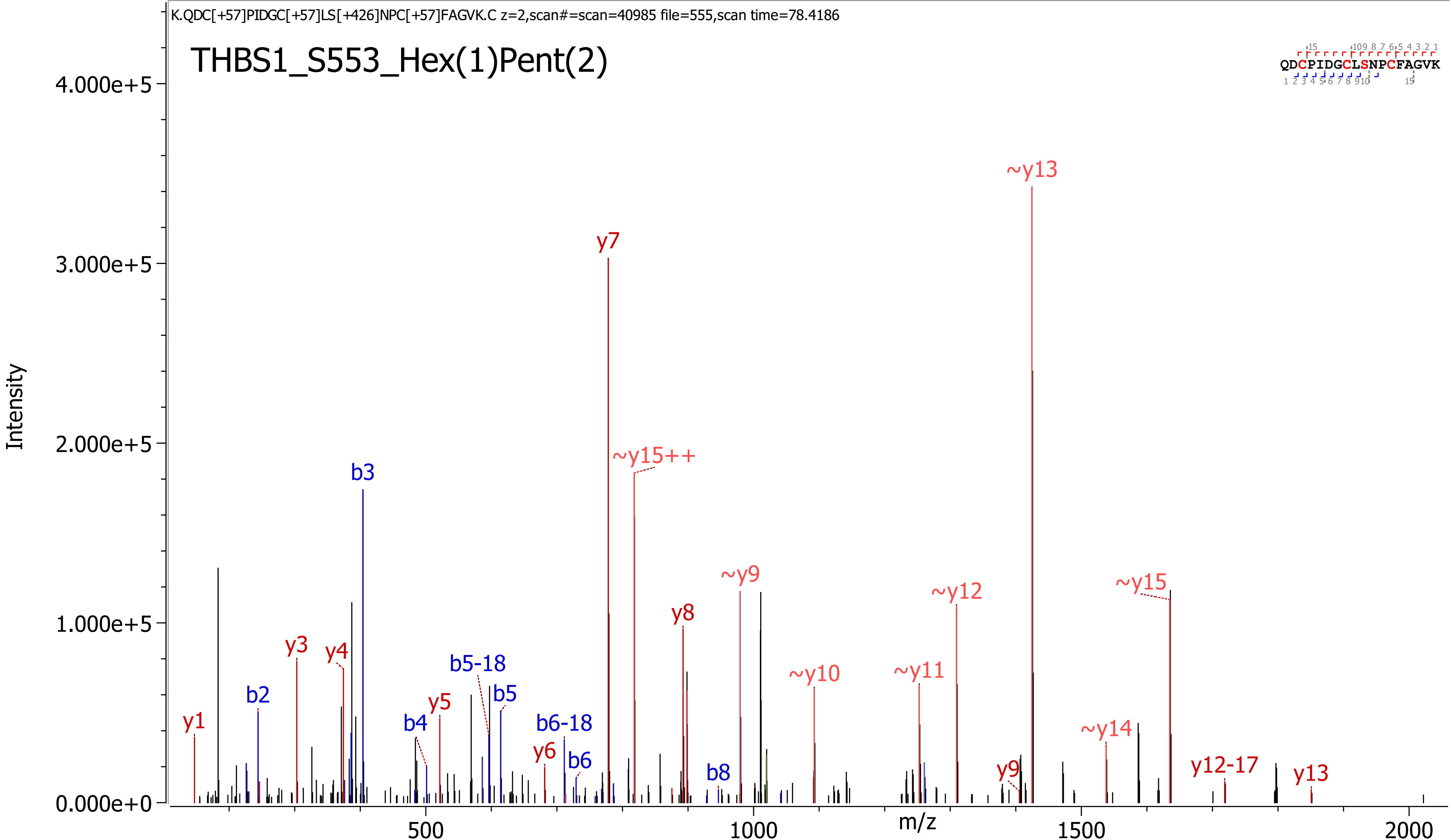
K.QDC[+57]PIDGC[+57]LS[+426]NPC[+57]FAGVK.C z=2,scan#=scan=40985 file=555,scan time=78.4186
THBS1_S553_Hex(1)Pent(2)
QDCPIDGCLSNPCFAGVK
Intensity
m/z
4.000e+5
3.000e+5
2.000e+5
1.000e+5
0.000e+0
500
1000
1500
2000
y1
b2
y3
y4
b3
b4
y5
b5-18
b5
y6
b6-18
b6
y7
~y15++
y8
b8
~y9
~y10
~y11
~y12
y9
~y13
~y14
~y15
y12-17
y13

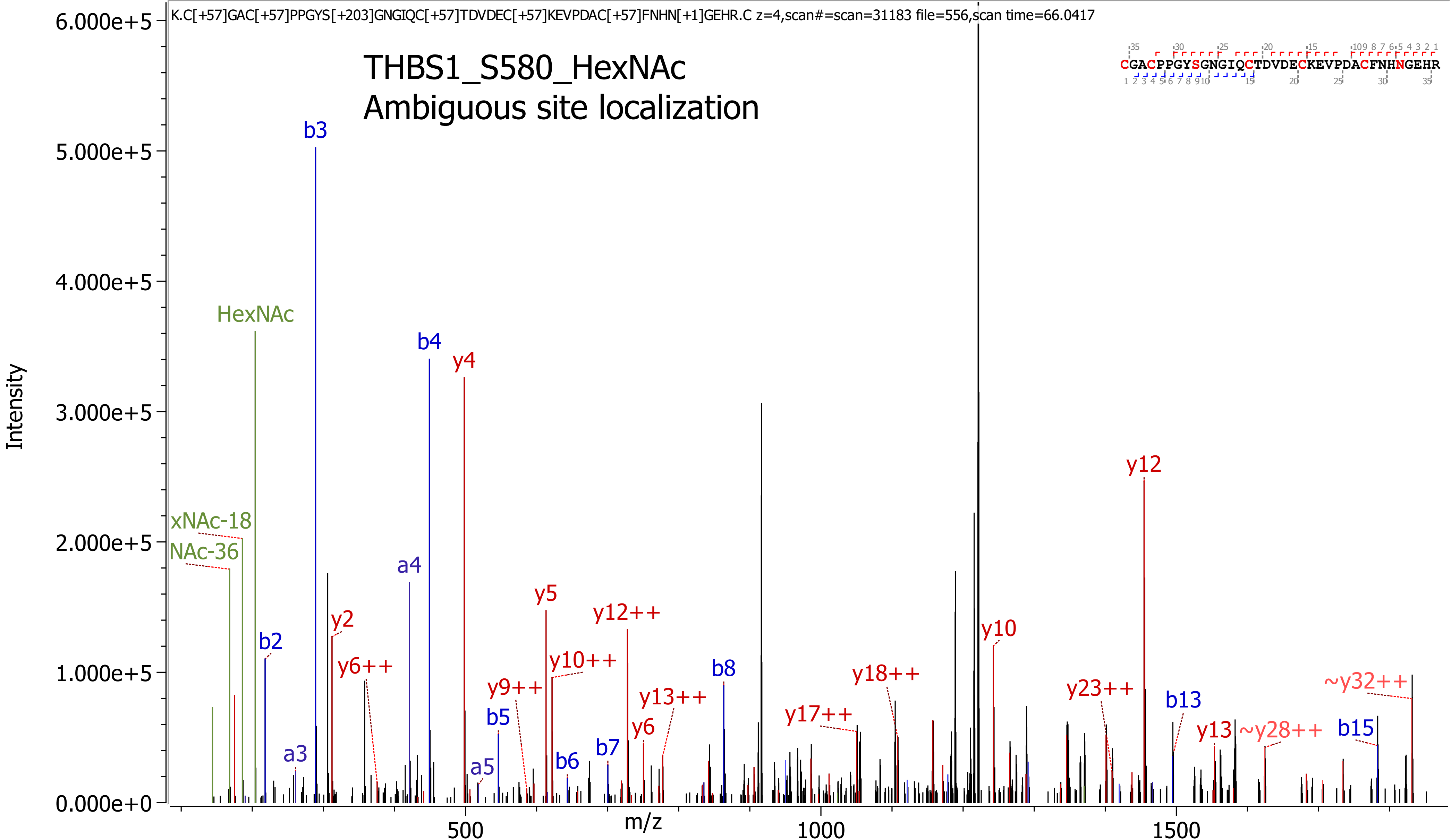

K.C[+57]GAC[+57]PPGYS[+203]GNGIQC[+57]TDVDEC[+57]KEVPDAC[+57]FNHN[+1]GEHR.C z=4,scan#=scan=31183 file=556,scan time=66.0417
THBS1_S580_HexNAc
Ambiguous site localization
CGACPPGYSGNGIQCTDVDECKEVPDACFNHNGEHR
Intensity
m/z
HexNAc
xNAc-18
NAc-36
b2
b3
b4
b5
b6
b7
b8
b13
b15
a3
a4
a5
y2
y4
y5
y6
y6++
y9++
y10
y10++
y12
y12++
y13
y13++
y17++
y18++
y23++
~y28++
~y32++

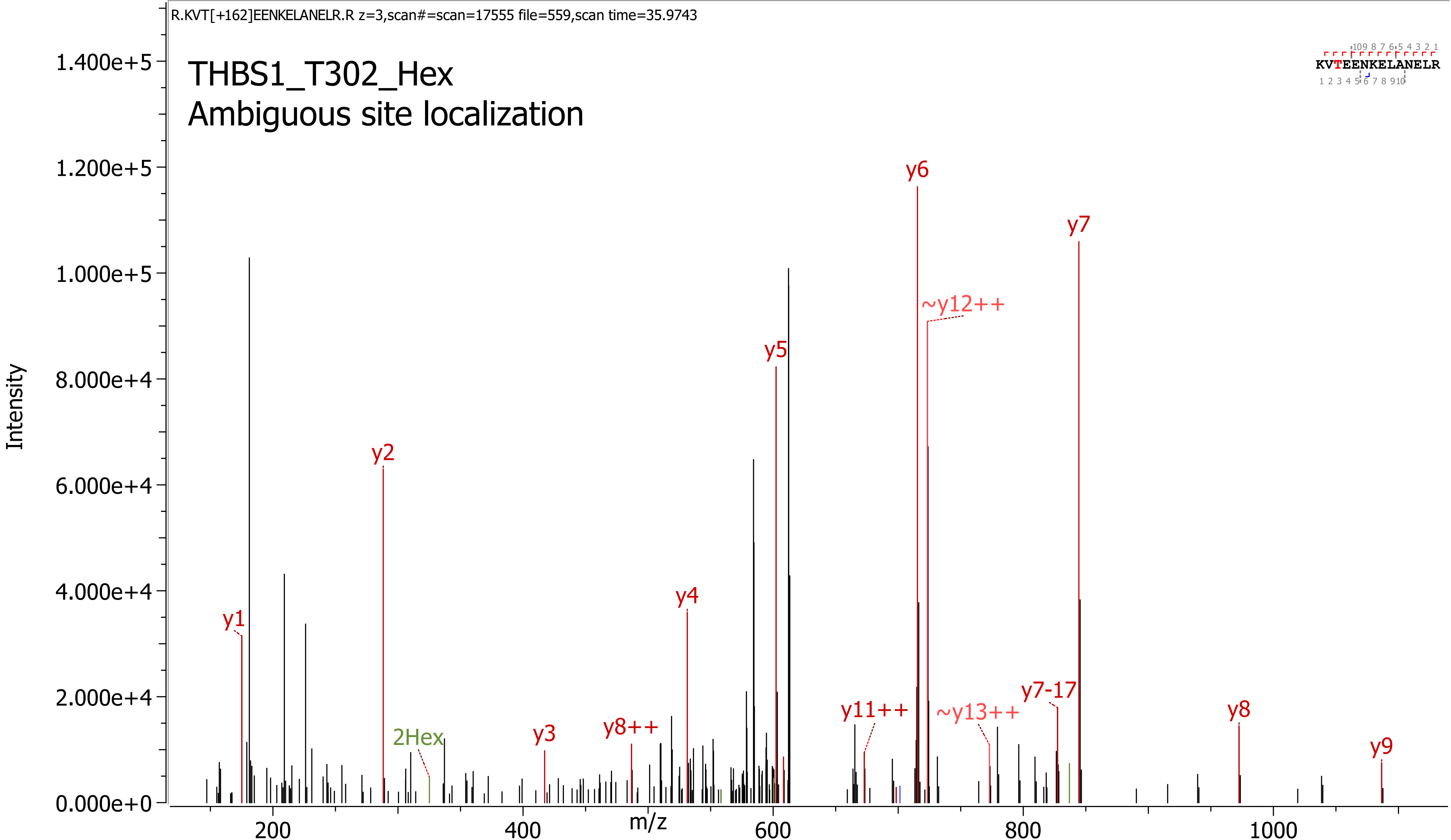

R.KVT[+162]EENKELANELR.R z=3,scan#=scan=17555 file=559,scan time=35.9743
THBS1_T302_Hex
Ambiguous site localization
KVTEENKELANELR
Intensity
m/z
y1
y2
2Hex
y3
y8++
y4
y5
y6
~y12++
y7
y11++
~y13++
y7-17
y8
y9

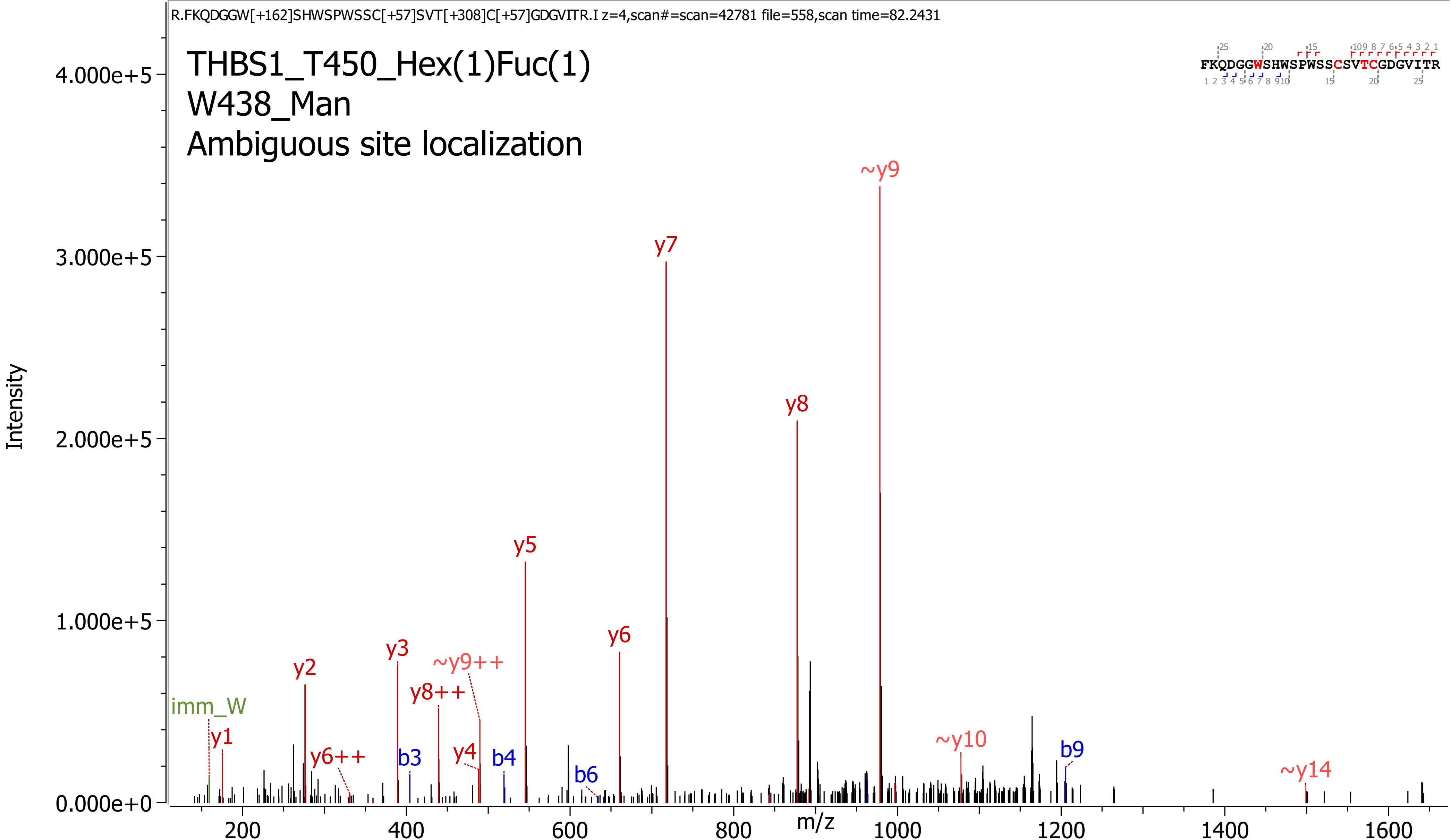
R.FKQDGGW[+162]SHWSPWSSC[+57]SVT[+308]C[+57]GDGVITR.I z=4,scan#=scan=42781 file=558,scan time=82.2431
THBS1_T450_Hex(1)Fuc(1)
W438_Man
Ambiguous site localization
FKQDGGWSHWSPWSSCSVTCGDGVITR
Intensity
m/z
~y9
y7
y8
y5
y6
y3
y2
~y9++
y8++
imm_W
y1
y6++
b3
y4
b4
b6
~y10
b9
~y14

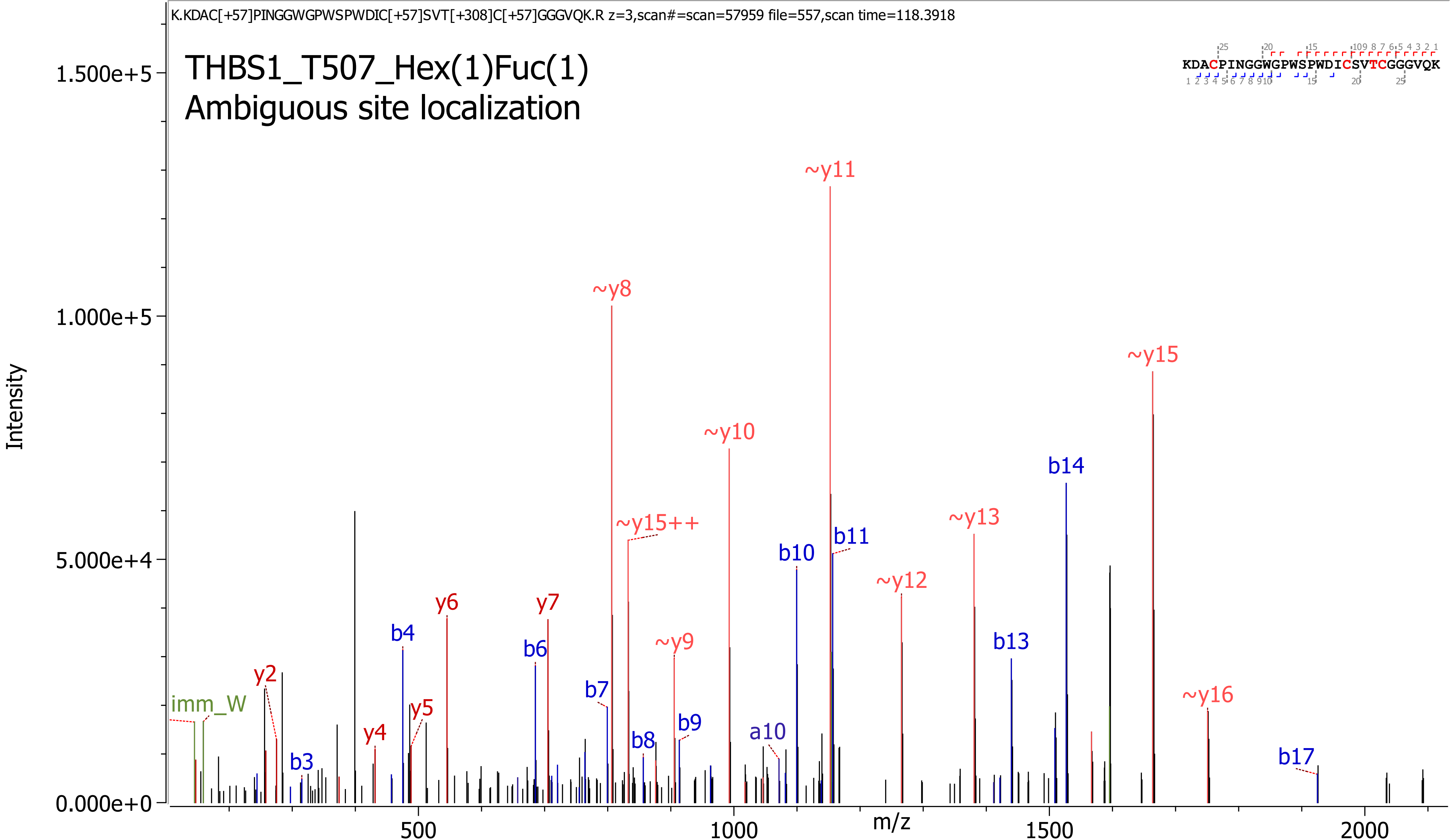
K.KDAC[+57]PINGGWGPWSPWDIC[+57]SVT[+308]C[+57]GGGVQK.R z=3,scan#=scan=57959 file=557,scan time=118.3918
THBS1_T507_Hex(1)Fuc(1)
Ambiguous site localization
KDACPINGGWGPWSPWDICSVTCGGGVQK
Intensity
m/z
~y11
~y8
~y10
~y15
b14
~y15++
~y13
b11
b10
~y12
y6
y7
b4
~y9
b6
b13
y2
~y16
imm_W
b7
y5
b9
a10
b8
y4
b17
b3
1.500e+5
1.000e+5
5.000e+4
0.000e+0
500
1000
1500
2000

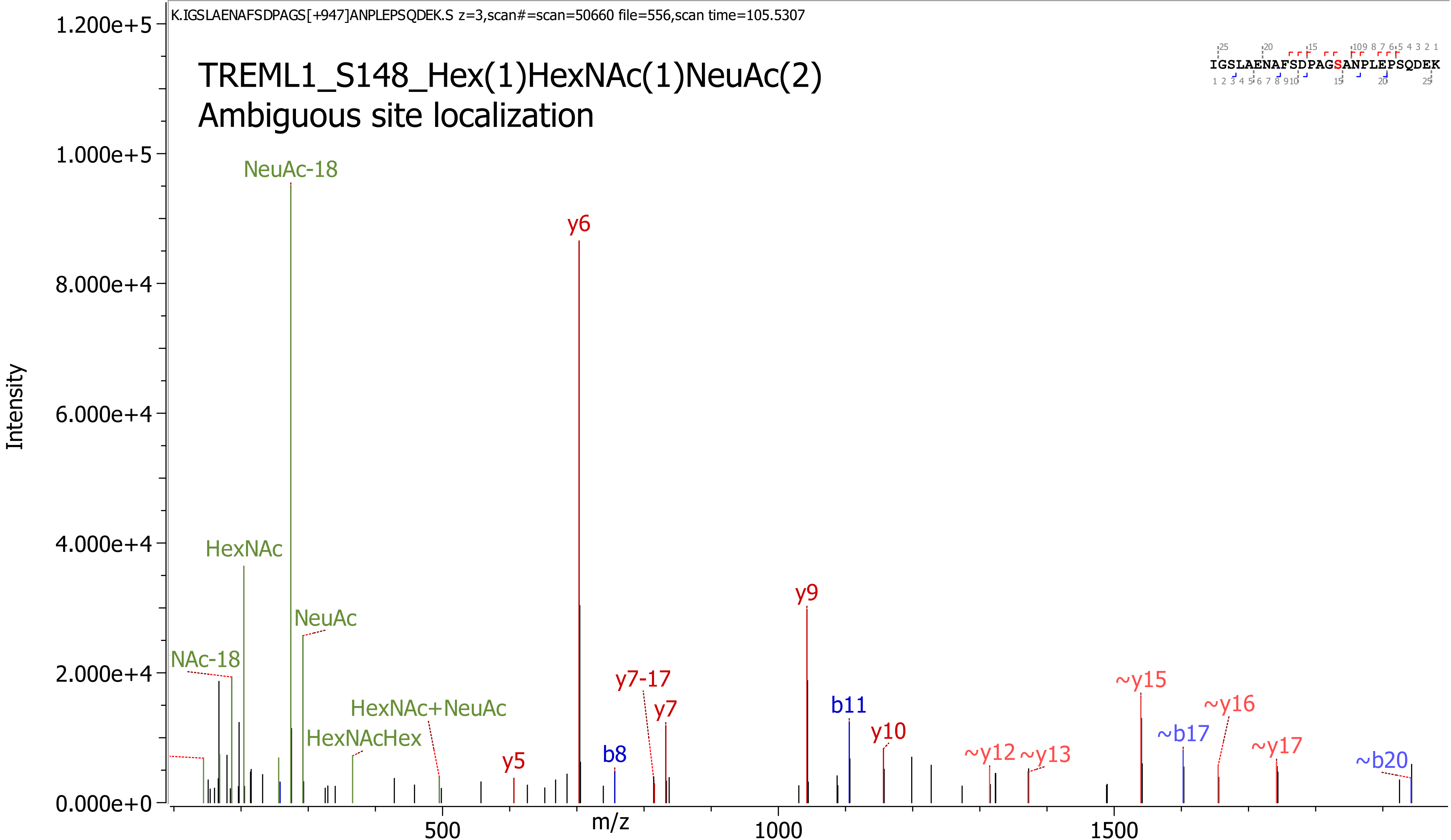

K.IGSLAENAFSDPAGS[+947]ANPLEPSQDEK.S z=3,scan#=scan=50660 file=556,scan time=105.5307
TREML1_S148_Hex(1)HexNAc(1)NeuAc(2)
Ambiguous site localization
IGSLAENAFSDPAGSANPLEPSQDEK
NeuAc-18
y6
HexNAc
NeuAc
NAc-18
HexNAc+NeuAc
HexNAcHex
y5
b8
y7-17
y7
y9
b11
y10
~y12
~y13
~y15
~y16
~b17
~y17
~b20
Intensity
m/z
1.200e+5
1.000e+5
8.000e+4
6.000e+4
4.000e+4
2.000e+4
0.000e+0
500
1000
1500

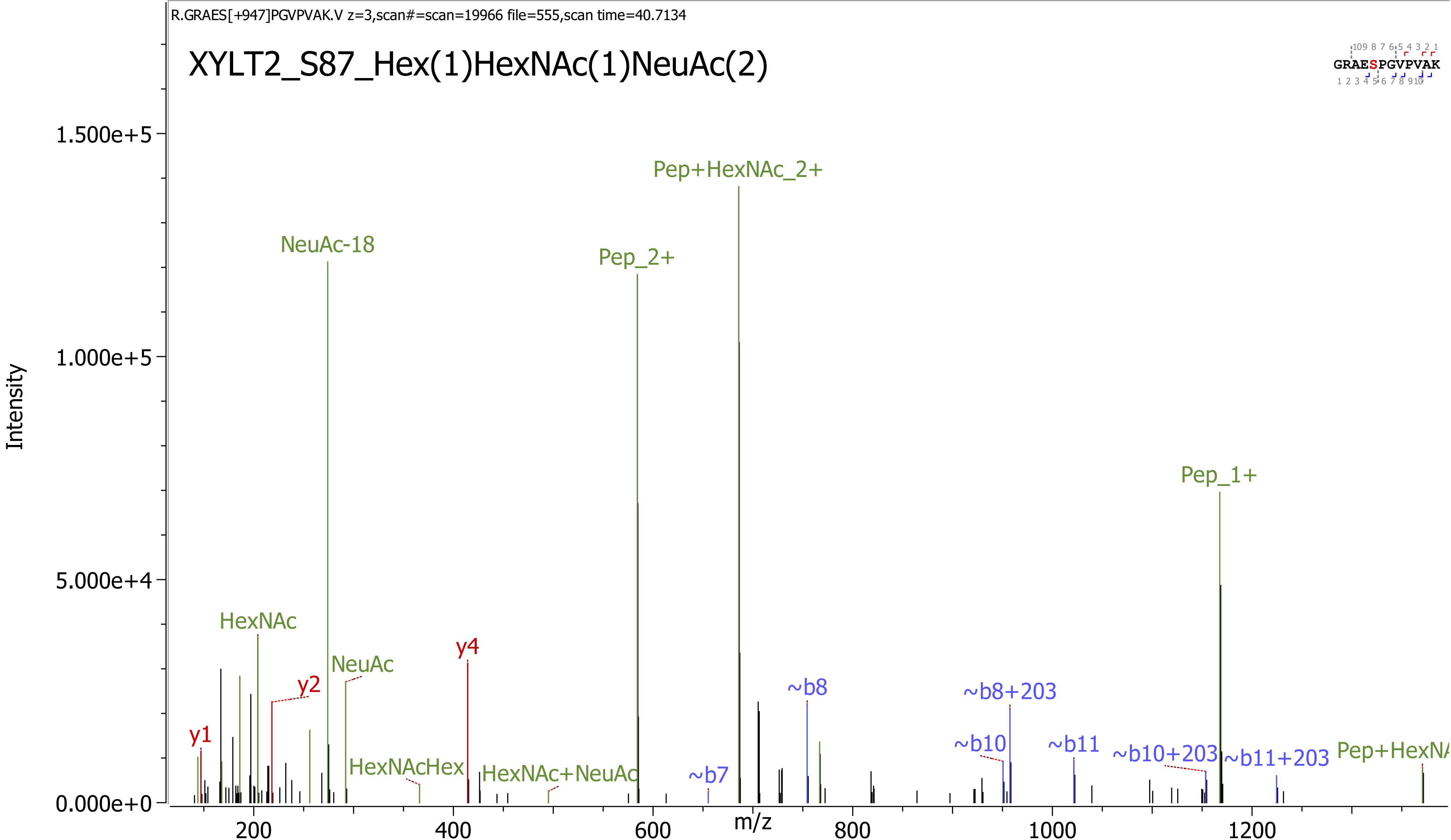

R.GRAES[+947]PGVPVAK.V z=3,scan#=scan=19966 file=555,scan time=40.7134
XYLT2_S87_Hex(1)HexNAc(1)NeuAc(2)
GRAESPGVPVAK
Intensity
m/z
NeuAc-18
Pep_2+
Pep+HexNAc_2+
Pep_1+
HexNAc
NeuAc
y1
y2
y4
HexNAcHex
HexNAc+NeuAc
~b7
~b8
~b8+203
~b10
~b11
~b10+203
~b11+203
