# Supplementary material for: Analysis of the Healthy Platelet Proteome Identifies a New Form of Domain-Specific O-Fucosylation
Source: Mol Cell Proteomics. 2024 Jan 16;23(2):100717. doi: 10.1016/j.mcpro.2024.100717 (PMC10879016; doi:10.1016/j.mcpro.2024.100717)
Supplement: Supplemental Figure 1 [file mmc9.pdf]

Supplementary Figure 1

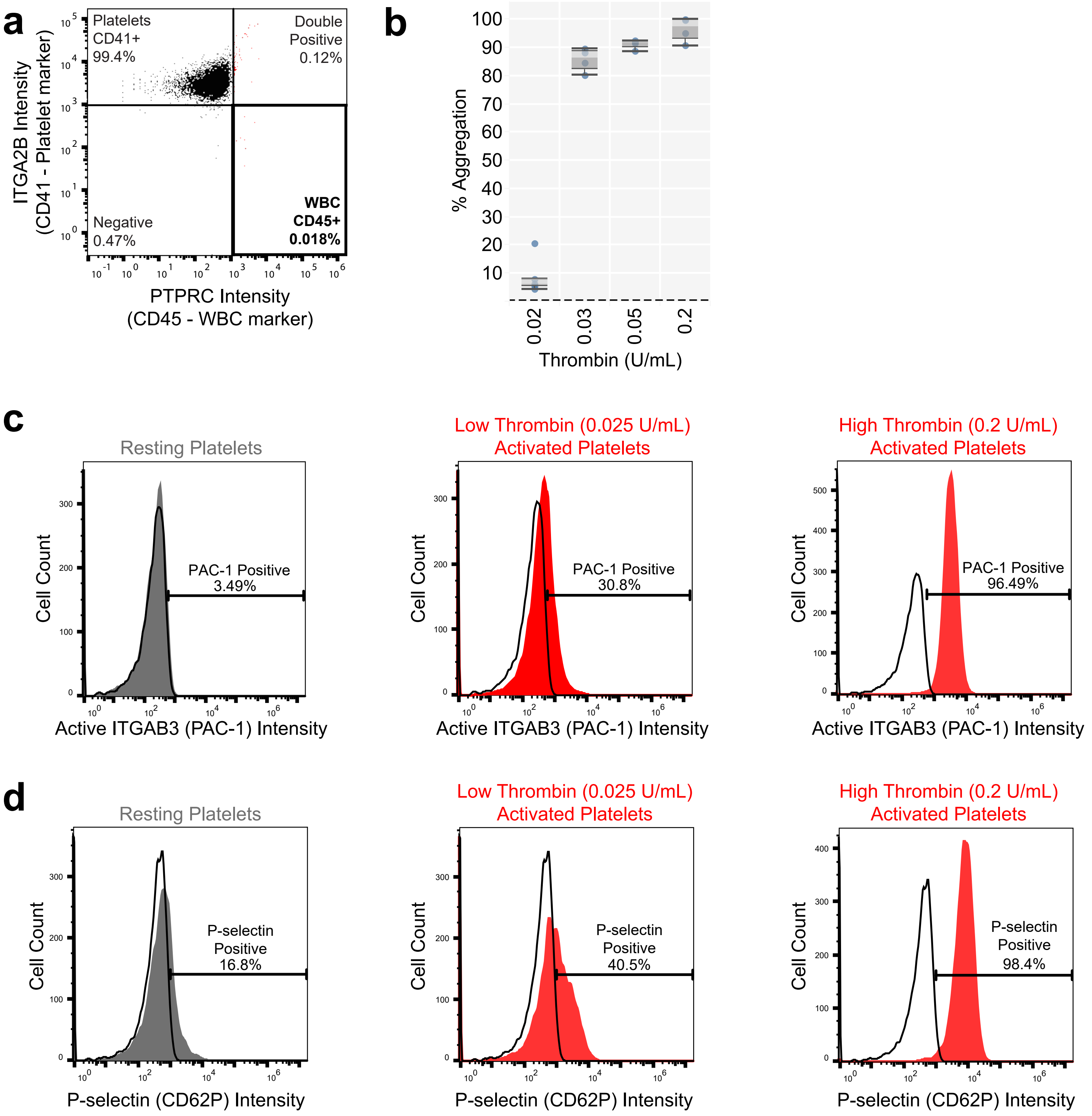

Supplementary Figure 1. Isolation of platelets in either the resting or thrombin-activated state for generation of platelet lysates and releasates. (a) Scatterplot of flow cytometry analysis for platelet contamination by white blood cells (WBC). (b) Analysis of platelet activation with a dose response of thrombin using aggregometry analysis. (c) Histograms of platelet activation using PAC-1 intensity (x-axis). Resting platelets are shown in grey, platelets stimulated with either 0.025 or 0.2 U/mL thrombin shown in red. (d) Histograms of platelet activation using P-selectin intensity (x-axis). Resting platelets are shown in grey, platelets stimulated with either 0.025 or 0.2 U/mL thrombin shown in red.
