# Supplementary material for: Analysis of the Healthy Platelet Proteome Identifies a New Form of Domain-Specific O-Fucosylation
Source: Mol Cell Proteomics. 2024 Jan 16;23(2):100717. doi: 10.1016/j.mcpro.2024.100717 (PMC10879016; doi:10.1016/j.mcpro.2024.100717)
Supplement: Supplemental Figure 2 [file mmc10.pdf]

Supplementary Figure 2

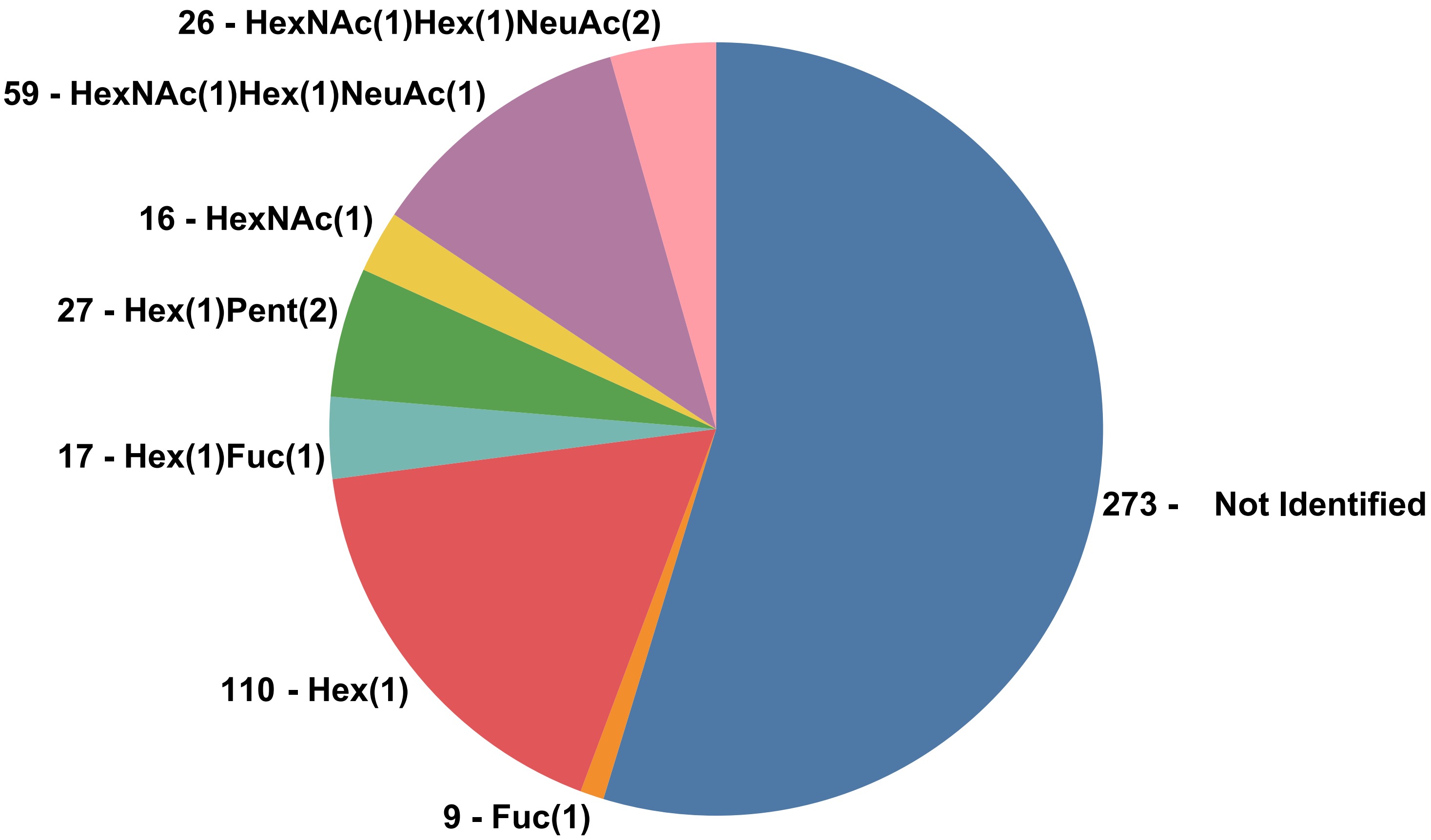

Supplementary Figure 2. Comparison of open-search and glycan-specific search for analysis of HCD spectra. Pie chart of the most frequently identified mass additions corresponding to *O*-glycosylation from the open search and the proportion of these also identified in a glycan-specific search. The first number indicates the the number of PSMs in that group, followed by the exact O-glycan modification identified by the glycan-specifc search. All data is from HCD fragmentation spectra.
