# Supplementary material for: Analysis of the Healthy Platelet Proteome Identifies a New Form of Domain-Specific O-Fucosylation
Source: Mol Cell Proteomics. 2024 Jan 16;23(2):100717. doi: 10.1016/j.mcpro.2024.100717 (PMC10879016; doi:10.1016/j.mcpro.2024.100717)

Supplementary File 3

Extracted ion chromatograms and manually annotated PGC-LC-ESI-CID-MS (-) spectra of *O*-glycans of platelet releasate activated with thrombin (0.2 U/mL)

Symbol, linkage and fragmentation key

- Fucose (Fuc)
- Galactose (Gal)
- Glucose (Glc)
- *N*-Acetylglucosamine (GlcNAc)
- *N*-Acetylgalactosamine (GalNAc)
- *N*-Acetylneuraminic acid (NeuAc)
- Xylose (Xyl)

- Indicates mostly Y ions (includes oxygen of glycosidic linkage)
- Indicates mostly Z ions (excludes oxygen of glycosidic linkage)
- Reduced reducing end
- a-b Isomers

Unknown linkage*

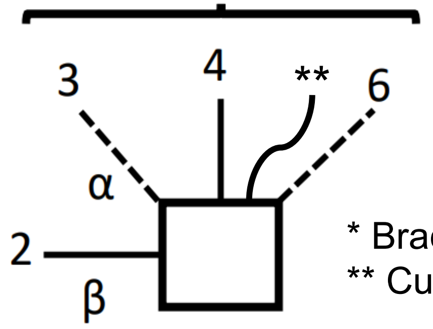

* Bracket denotes unknown branch point / topology

** Curve line denotes unknown glycosidic linkage

Glycan #1
Extracted ion chromatogram
(*m/z* 327.10-327.15, MS1)

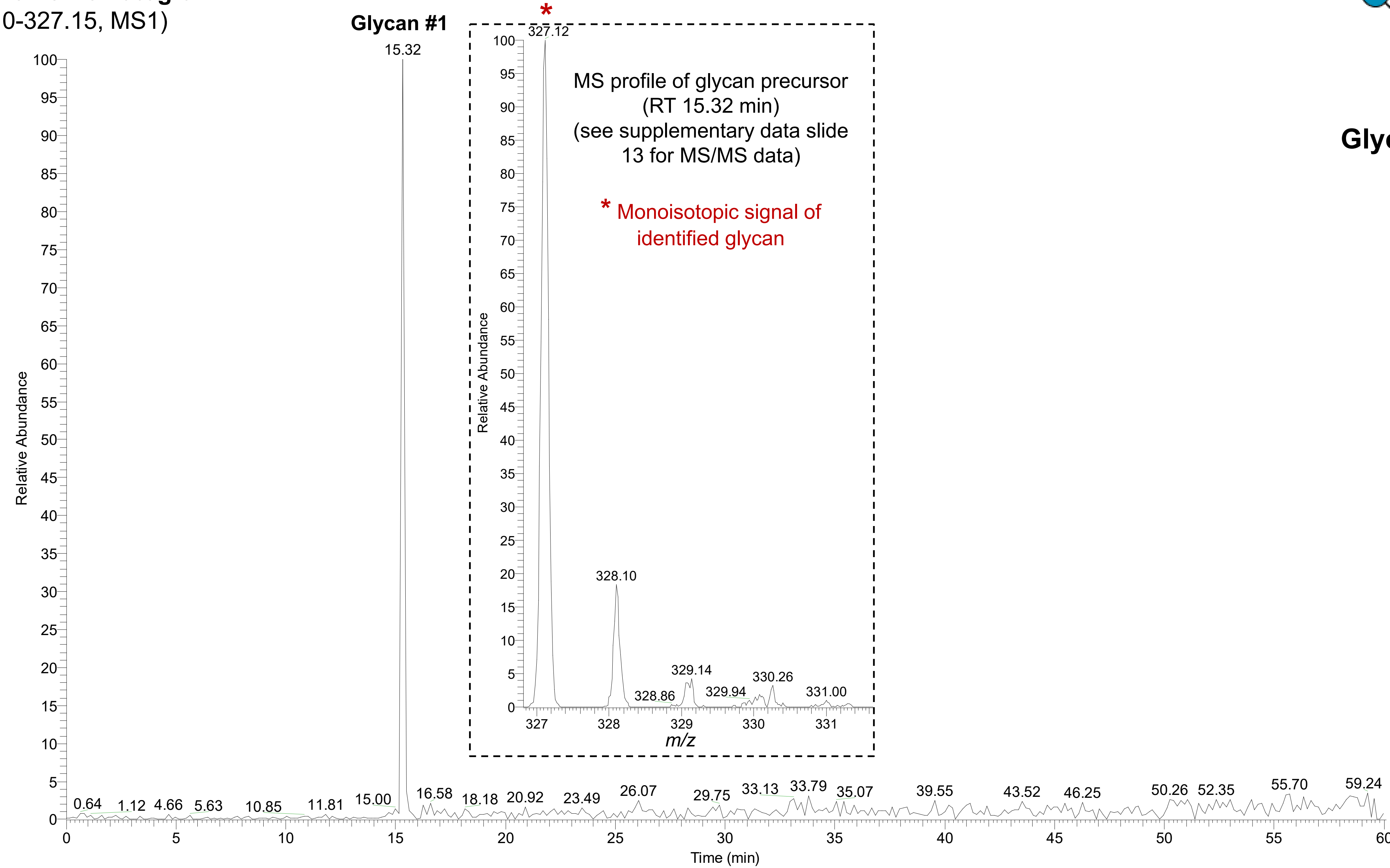

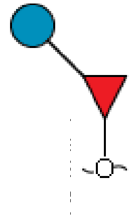

Glycan #1

Glycan #2
Extracted ion chromatogram
(*m/z* 445.10-445.15, MS1)

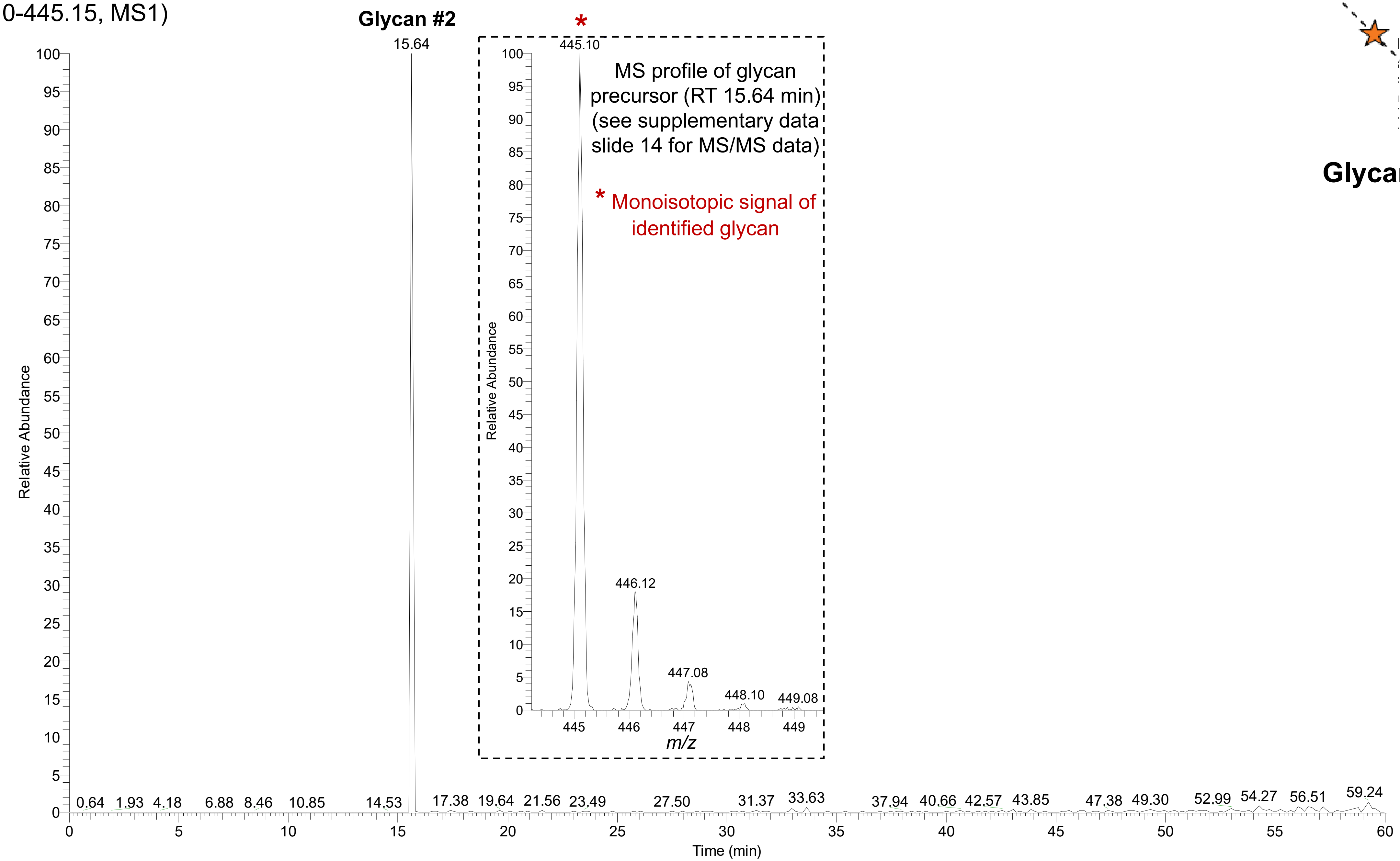

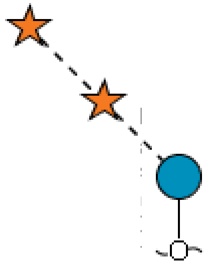

Glycan #2

Glycan #3
Extracted ion chromatogram
(*m/z* 675.15-675.20, MS1)

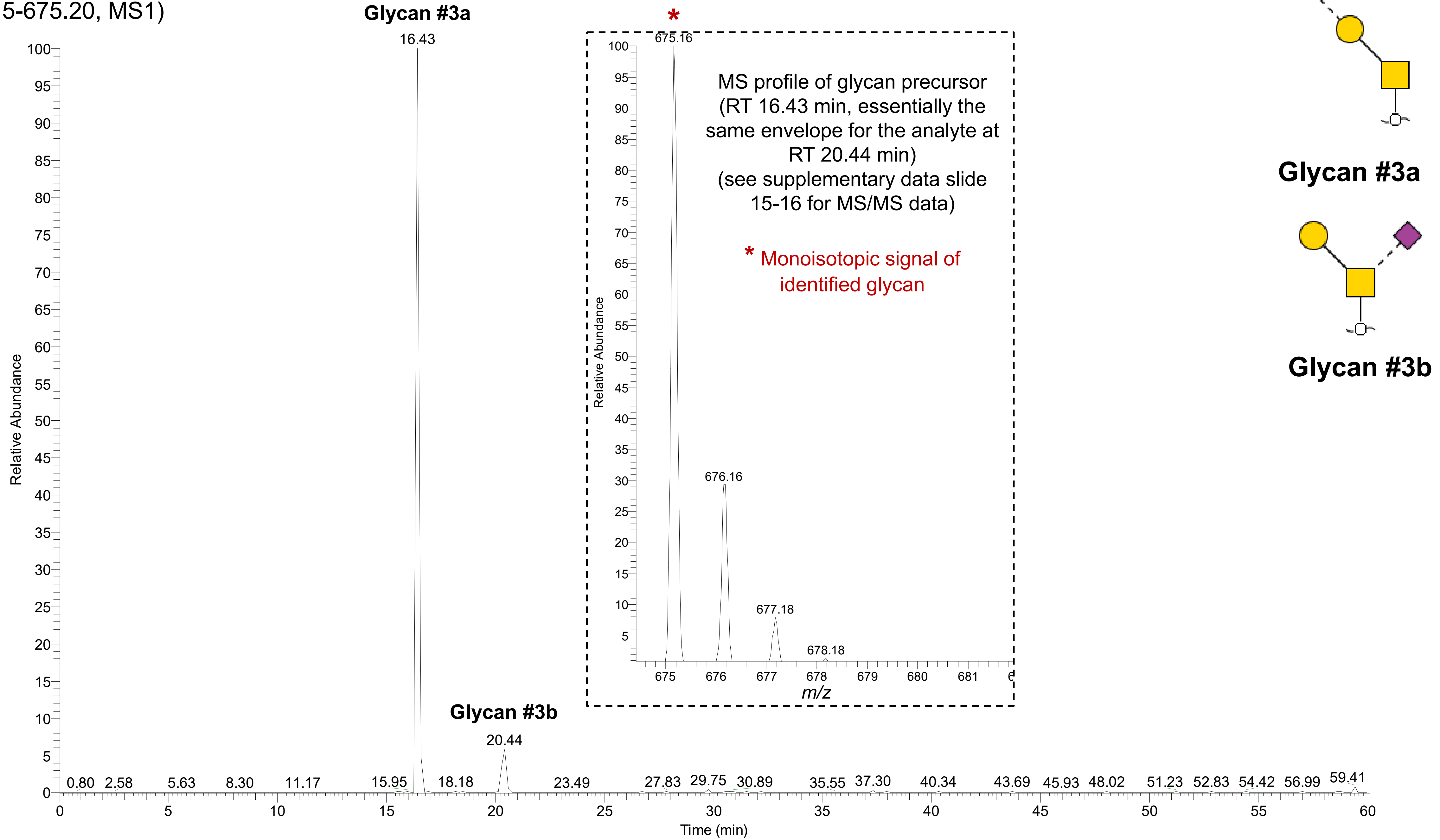

Glycan #4
Extracted ion chromatogram
(*m/z* 878.20-878.25, MS1)

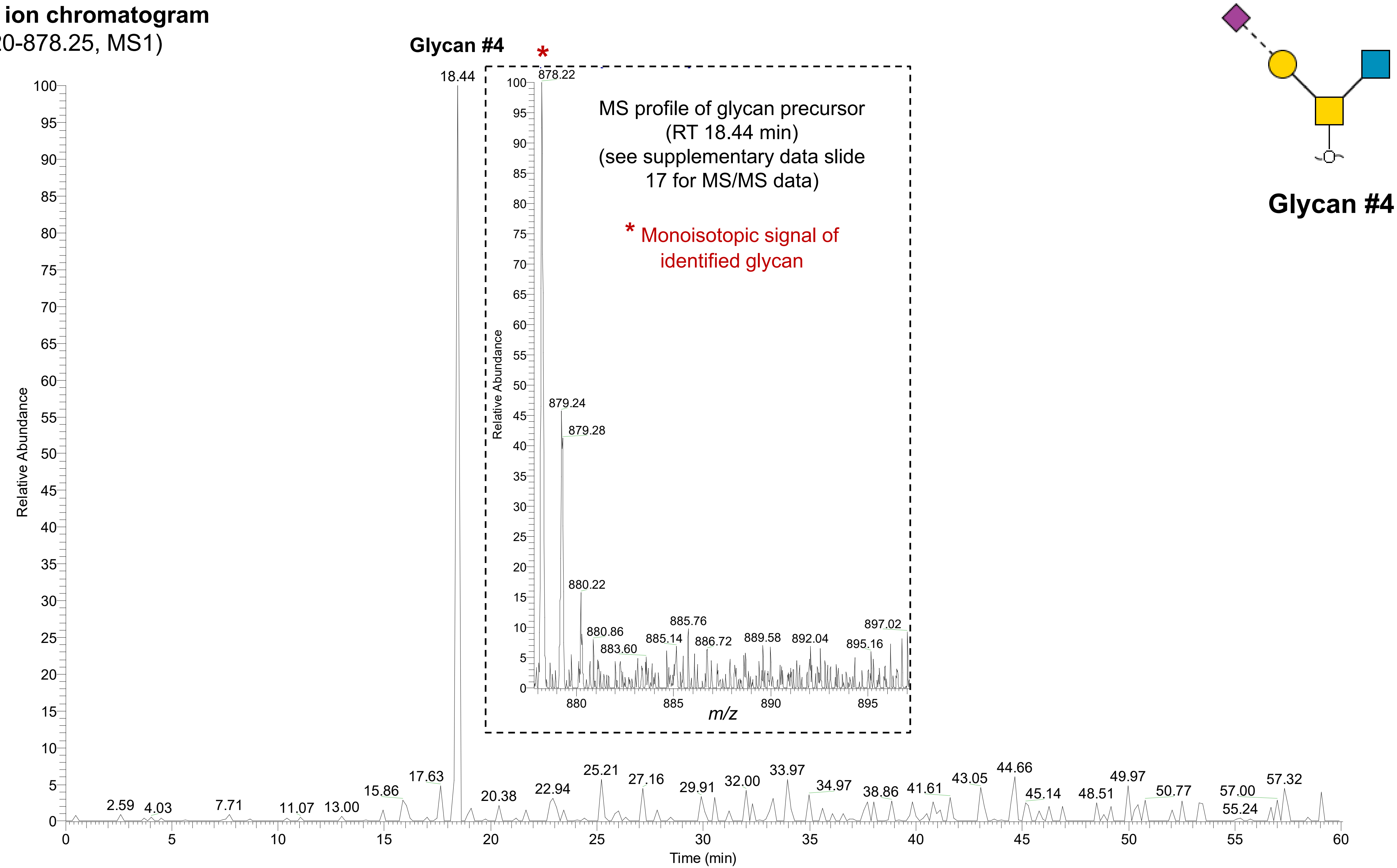

Glycan #4

Glycan #5
Extracted ion chromatogram
(*m/z* 966.25-966.28, MS1)

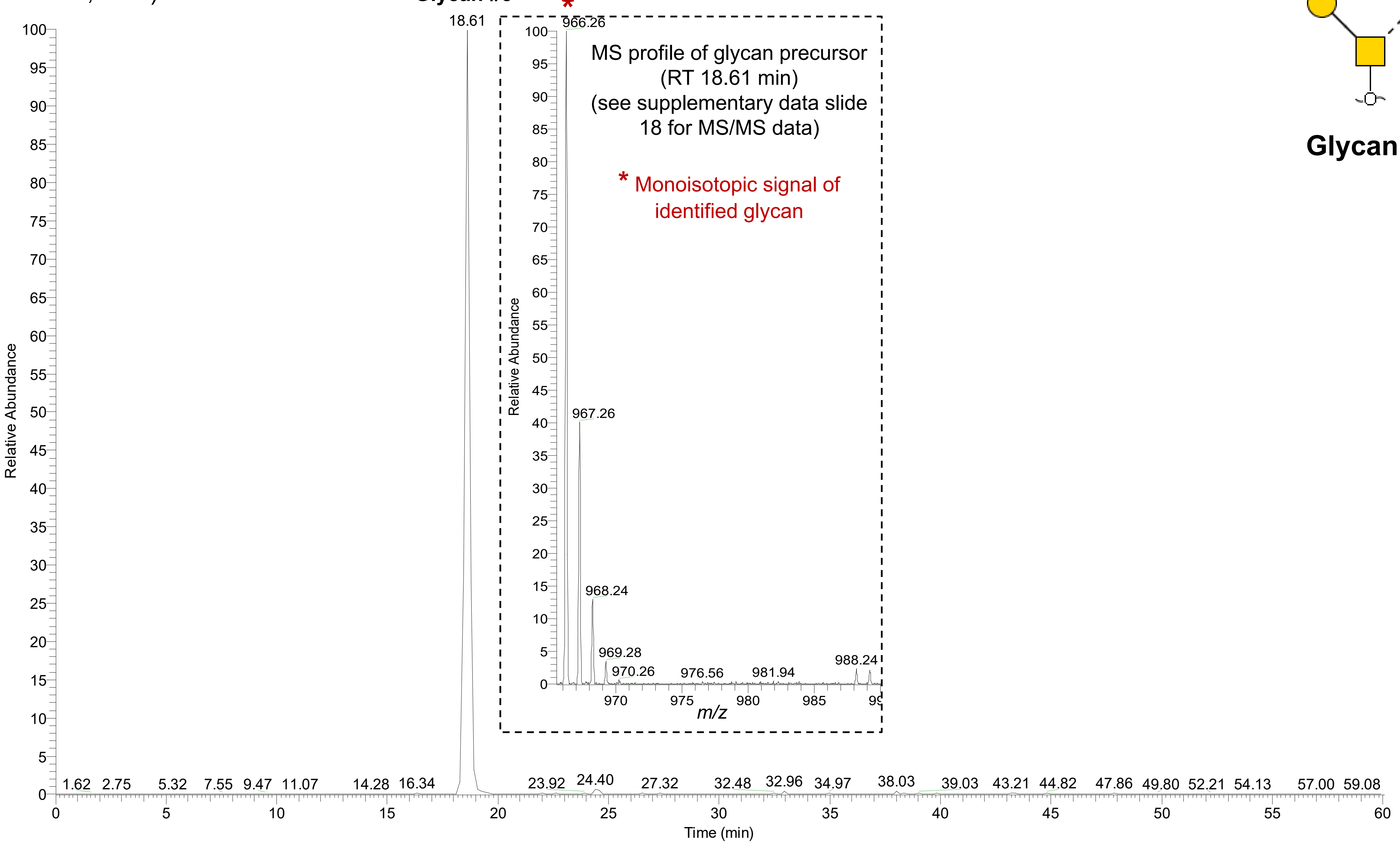

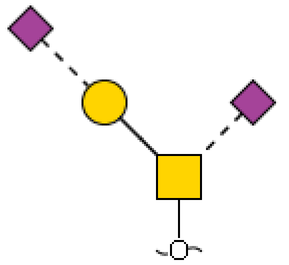

Glycan #5

Glycan #6
Extracted ion chromatogram
(*m/z* 1040.30-1040.35, MS1)

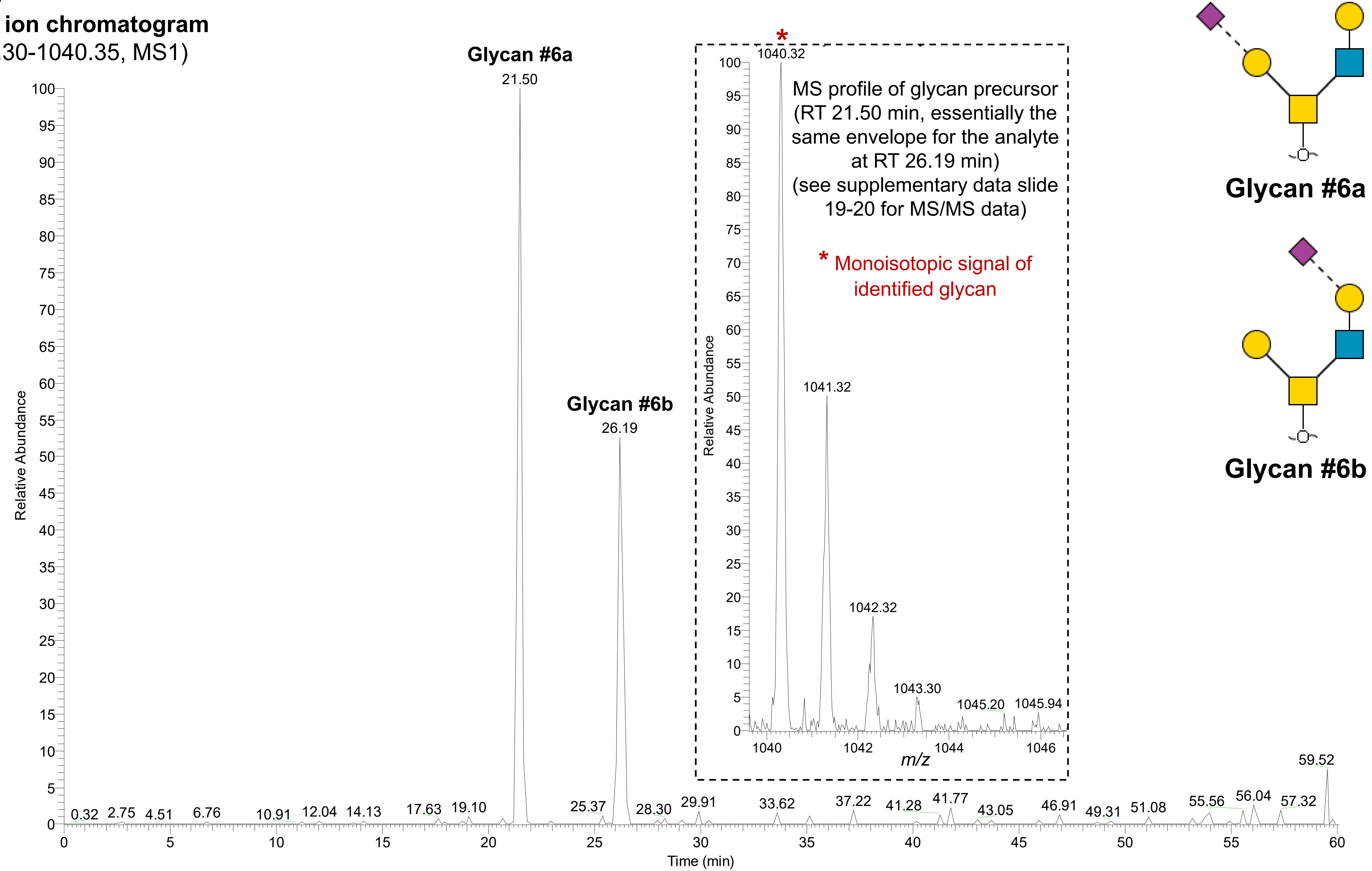

Glycan #7
Extracted ion chromatogram
(*m/z* 1186.36-1186.39, MS1)

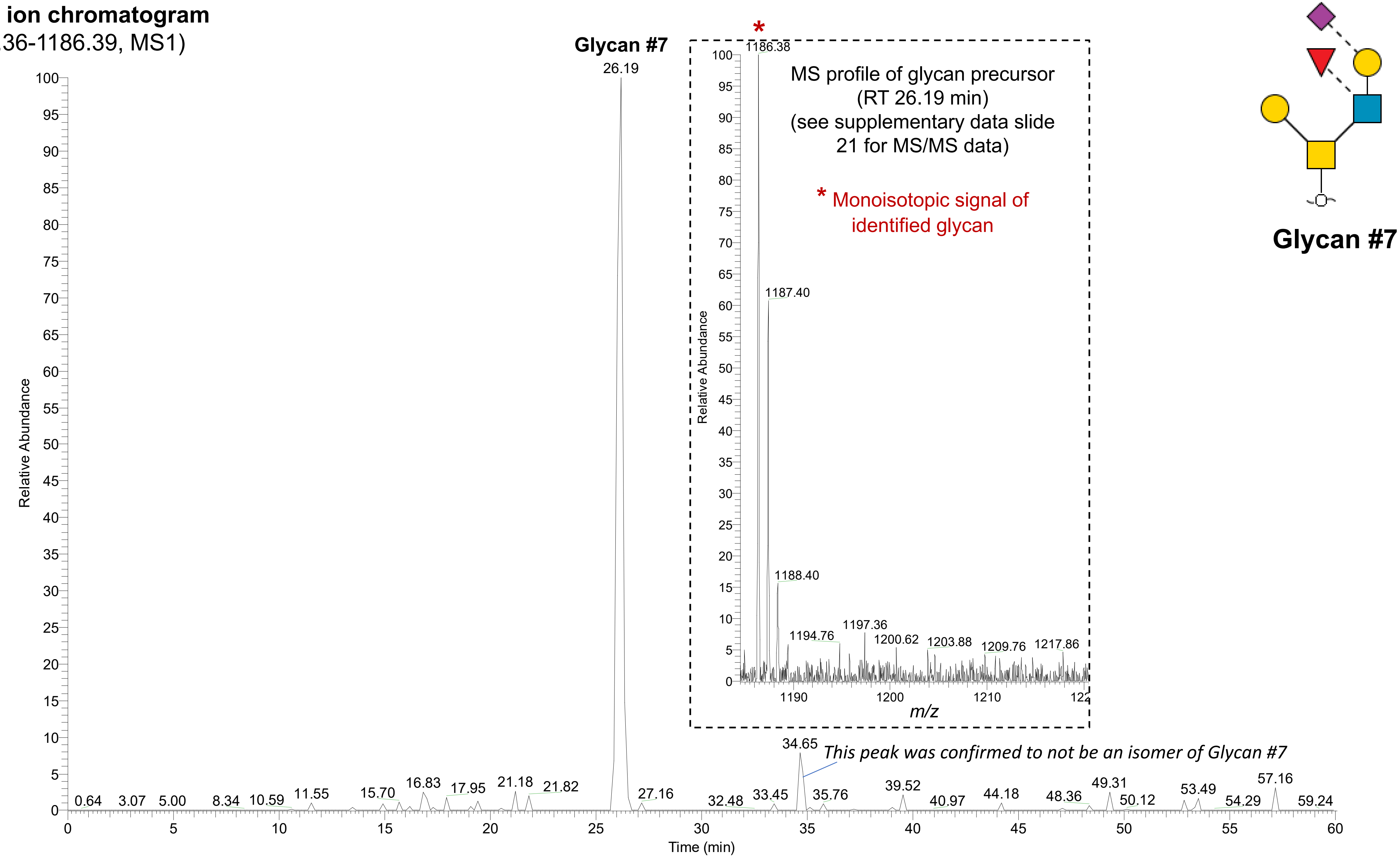

Glycan #7

Glycan #8
Extracted ion chromatogram
(*m/z* 1257.37-1257.41, MS1)

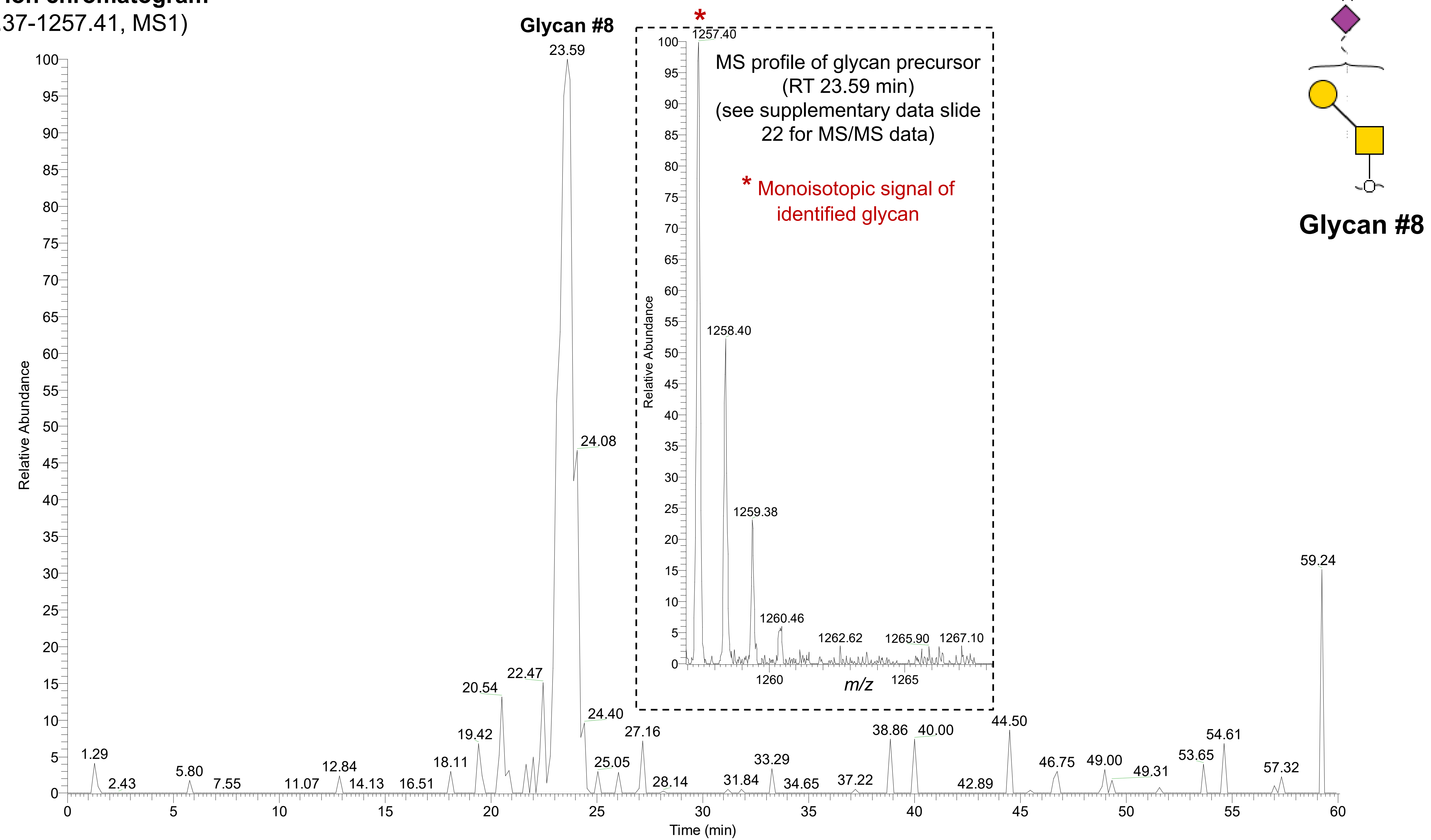

Glycan #9
Extracted ion chromatogram
(*m/z* 1331.42-1331.45, MS1)

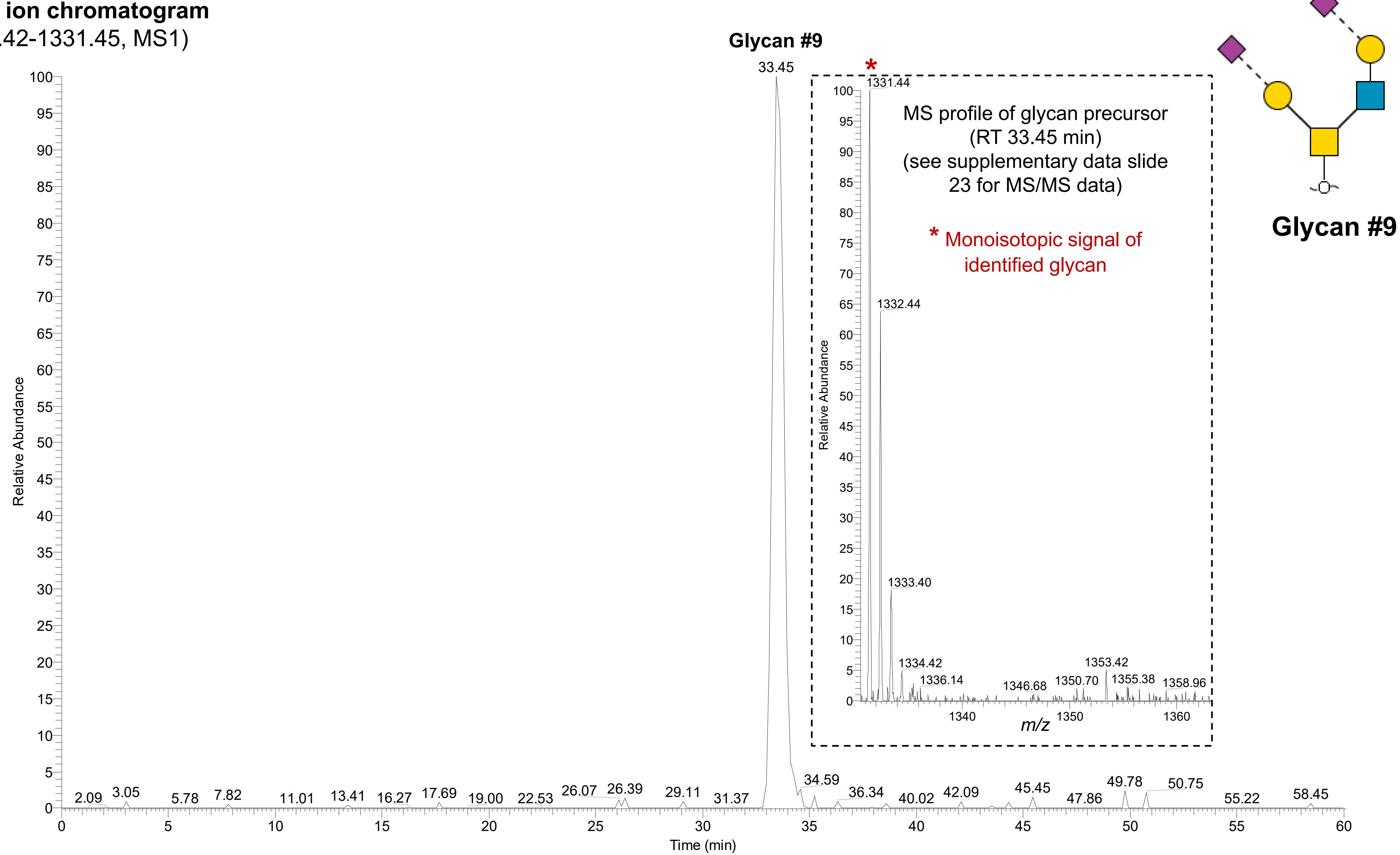

Glycan #9

Manually annotated PGC-LC-ESI-CID-MS/MS (-)
spectra of *O*-glycans of platelet releasate activated with thrombin (0.2 U/mL)

Glycan #1
Observed *m/z* 327.12 (1-), RT: ~15.44 min
Observed [M] 328.13 Da
Theoretical [M] 328.11 Da

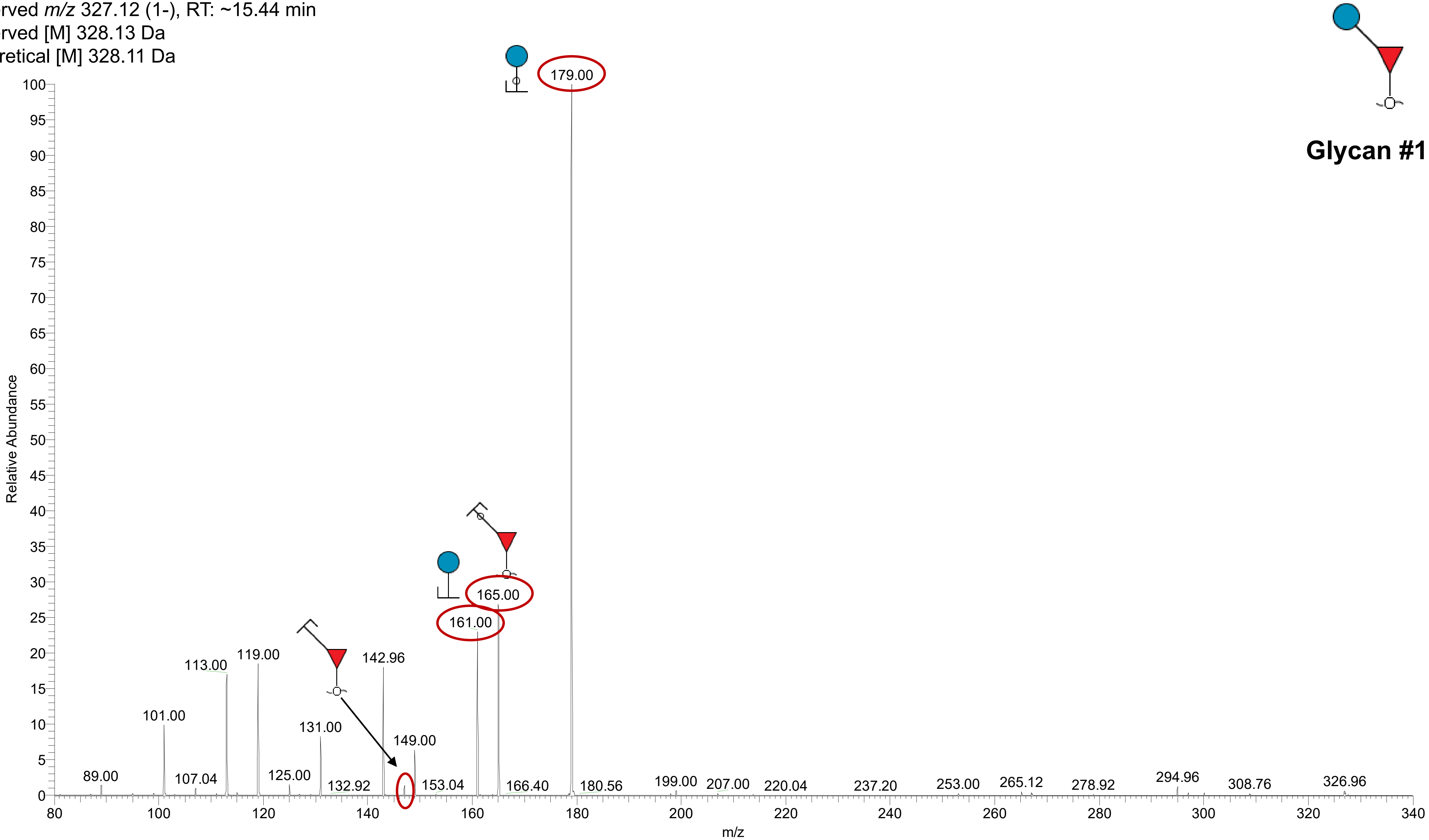

Glycan #2

Observed *m/z* 445.10 (1-), RT: ~15.68 min

Observed [M] 446.11

Theoretical [M] 446.14

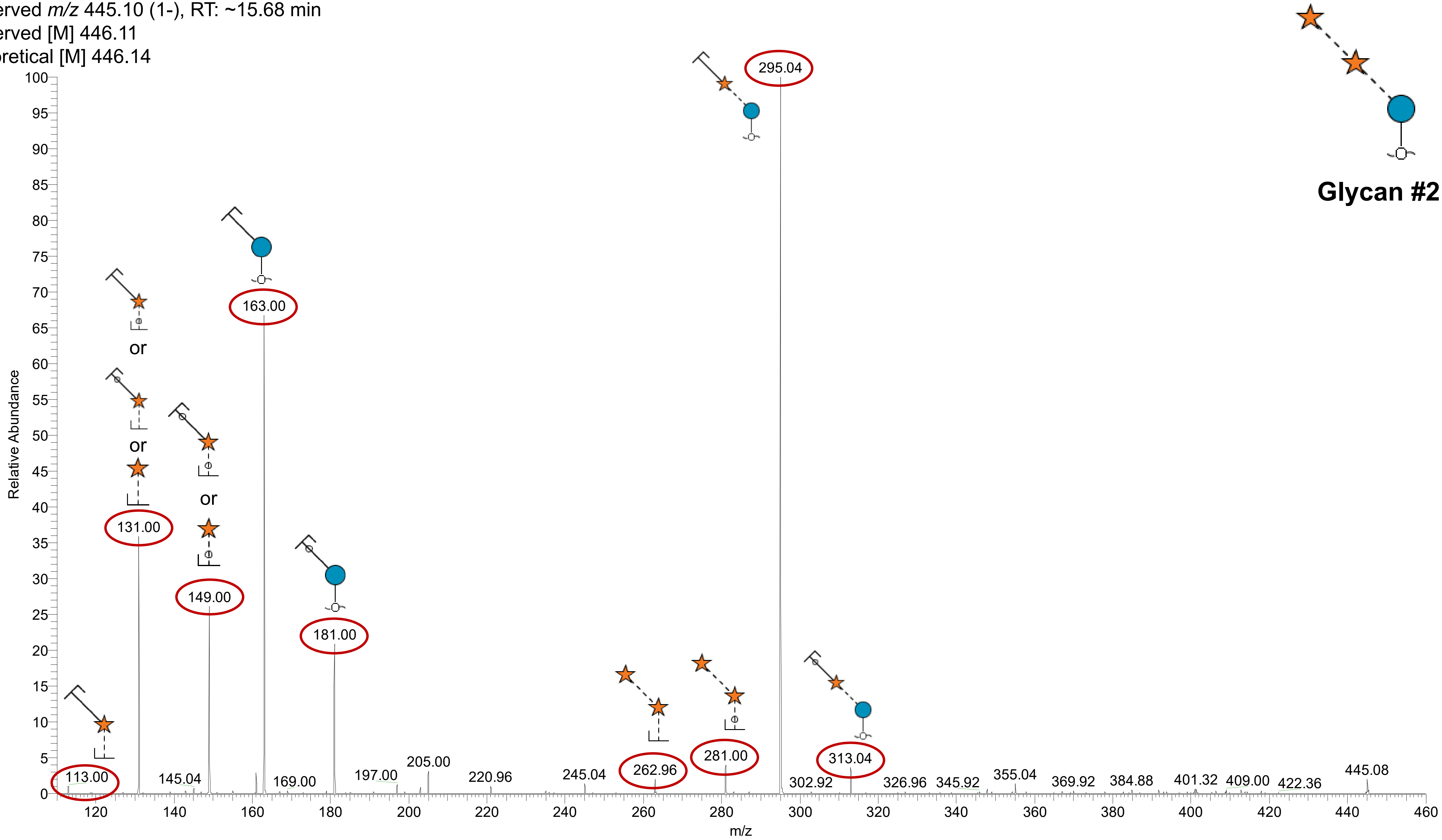

Glycan #3a
Observed *m/z* 675.16 (1-), RT: ~16.43 min
Observed [M] 676.17 Da
Theoretical [M] 676.23 Da

Note: The glycan isomers #3a and #3b are only distinguishable via PGC-LC retention time and previous literature (see Table above for details).

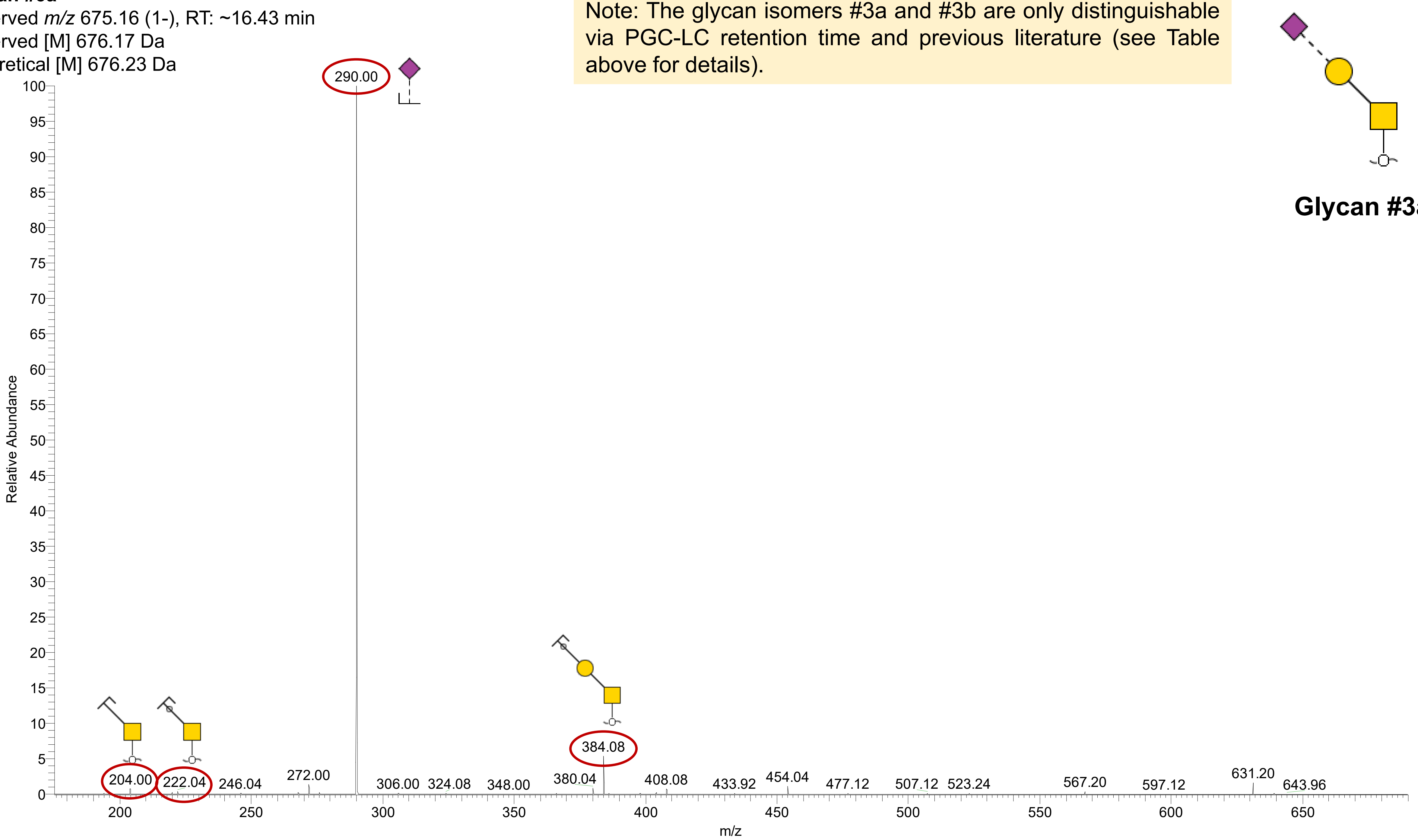

Glycan #3a

Glycan #3b
Observed *m/z* 675.16 (1-), RT: ~20.45 min
Observed [M] 676.17 Da
Theoretical [M] 676.23 Da

Note: The glycan isomers #3a and #3b are only distinguishable via PGC-LC retention time and previous literature (see Table above for details).

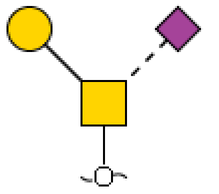

Glycan #3b

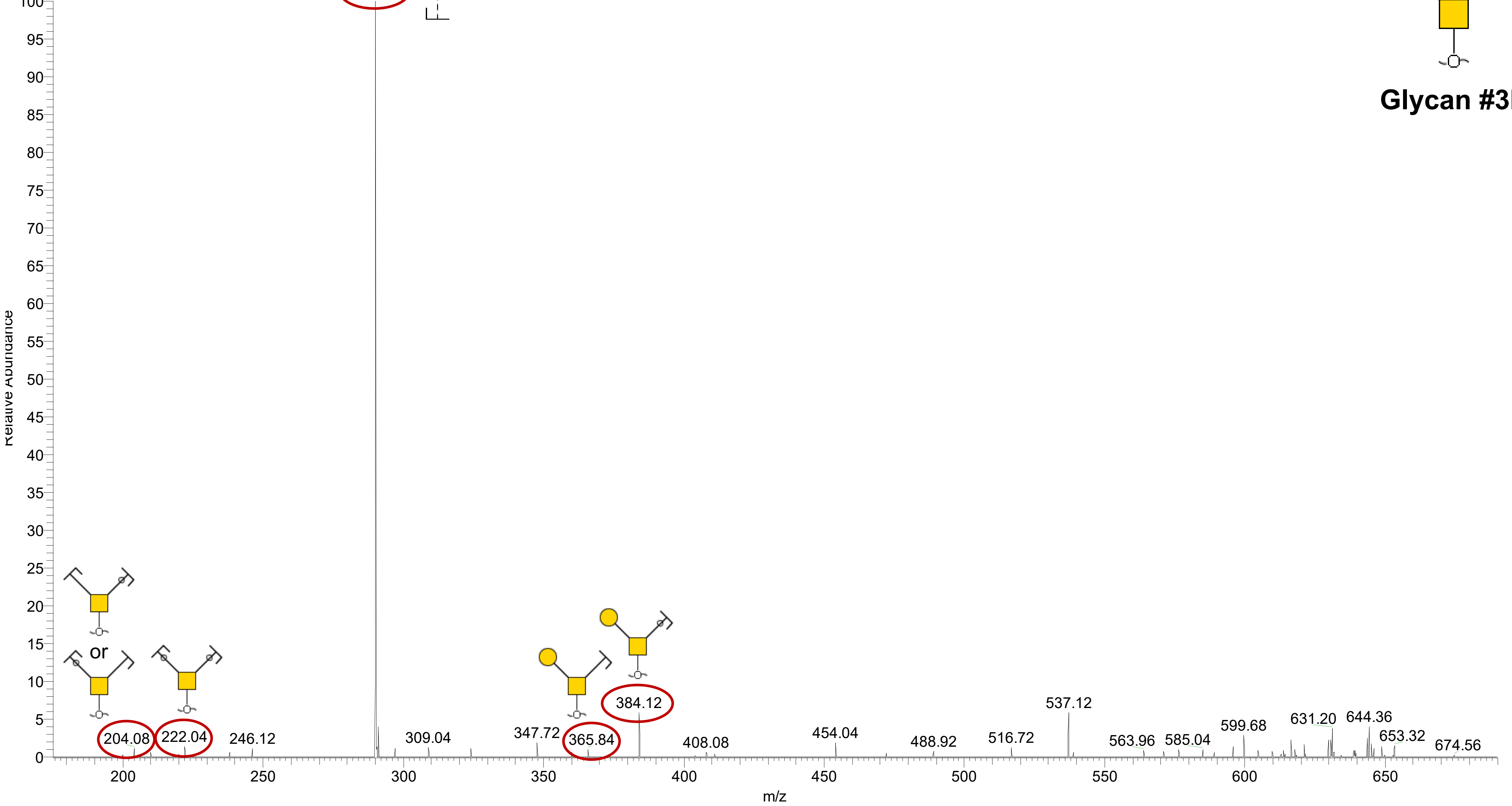

Glycan #4
Observed *m/z* 878.22 (1-), RT: ~18.49 min
Observed [M] 879.23 Da
Theoretical [M] 879.31 Da

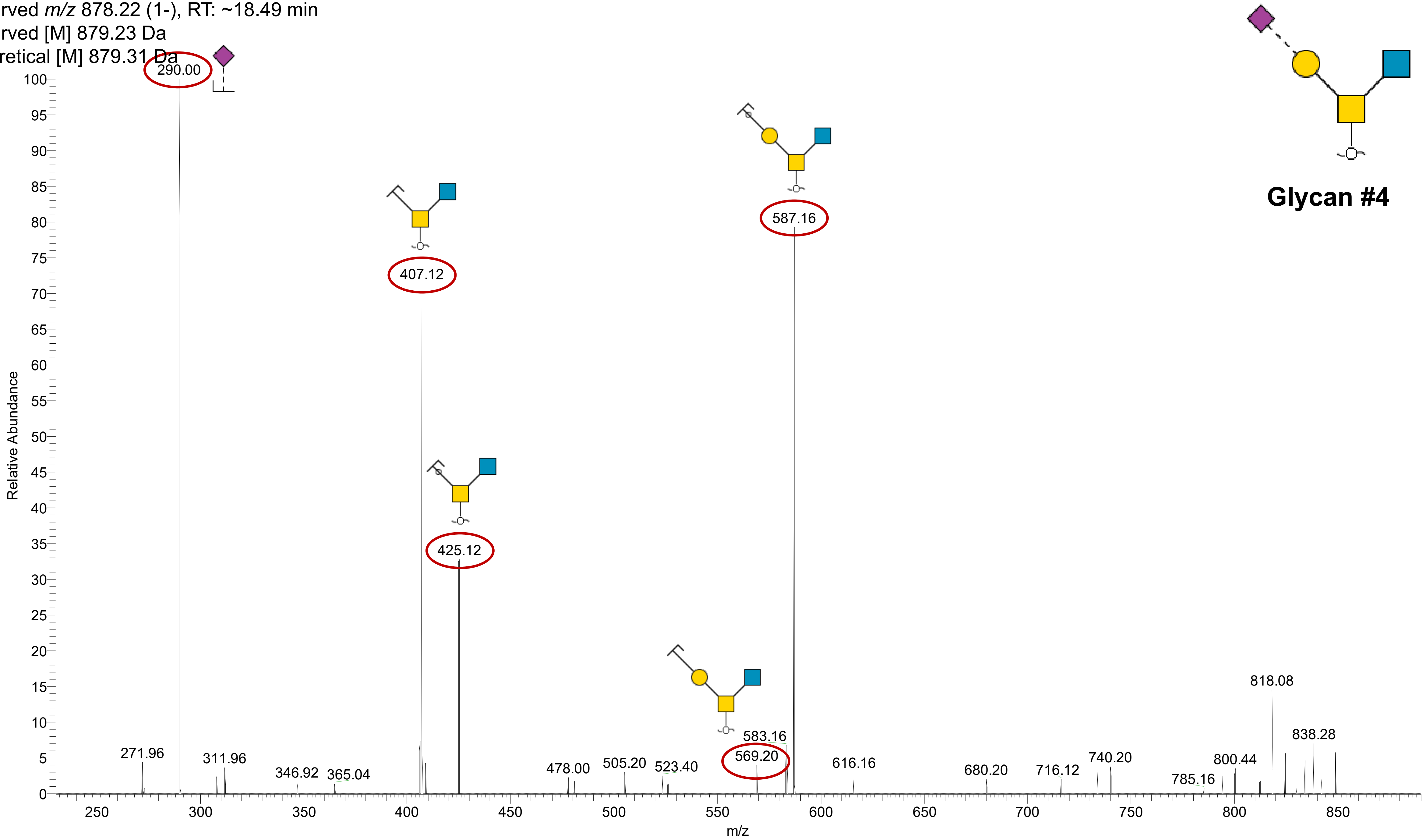

Glycan #5
Observed *m/z* 966.26 (1-), RT: ~18.35 min
Observed [M] 967.27 Da
Theoretical [M] 967.32 Da

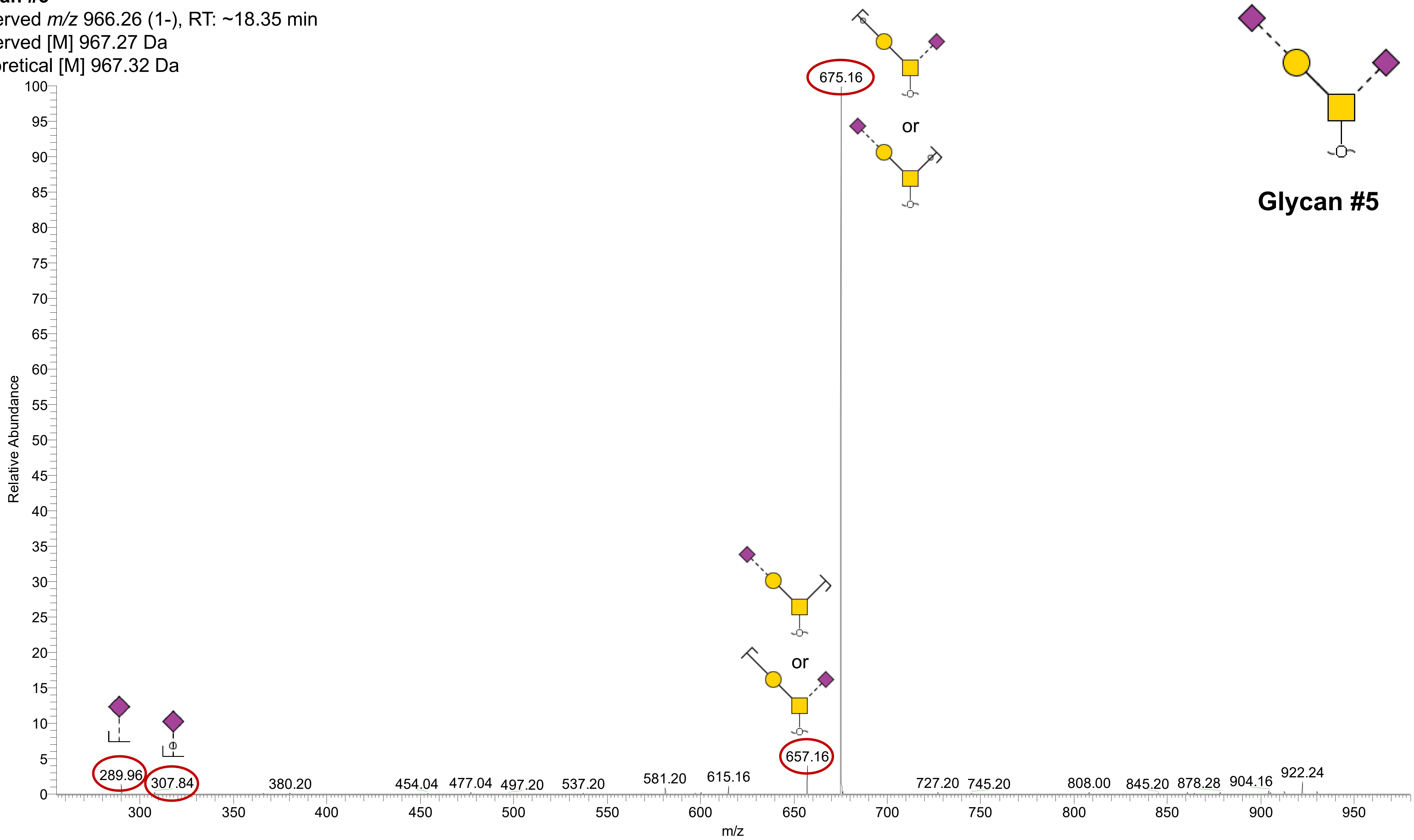

Glycan #6a
Observed *m/z* 1040.31 (1-), RT: ~21.39 min
Observed [M] 1041.33 Da
Theoretical [M] 1041.36 Da

Note: The glycan isomers #6a and #6b are only distinguishable via PGC-LC retention time.

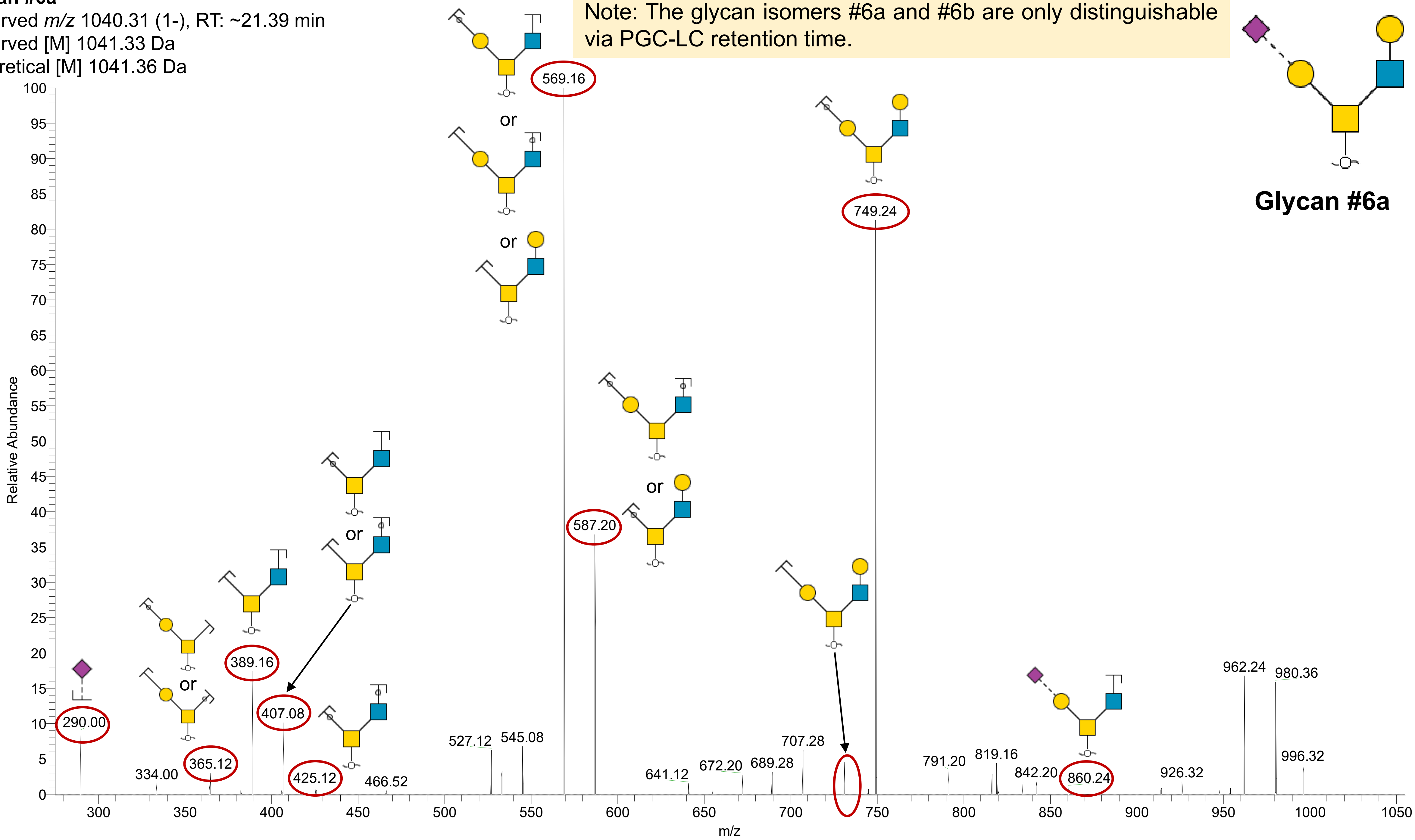

Glycan #6b
Observed *m/z* 1040.31 (1-), RT: ~26.16 min
Observed [M] 1041.33 Da
Theoretical [M] 1041.36 Da

Note: The glycan isomers #6a and #6b are only distinguishable via PGC-LC retention time.

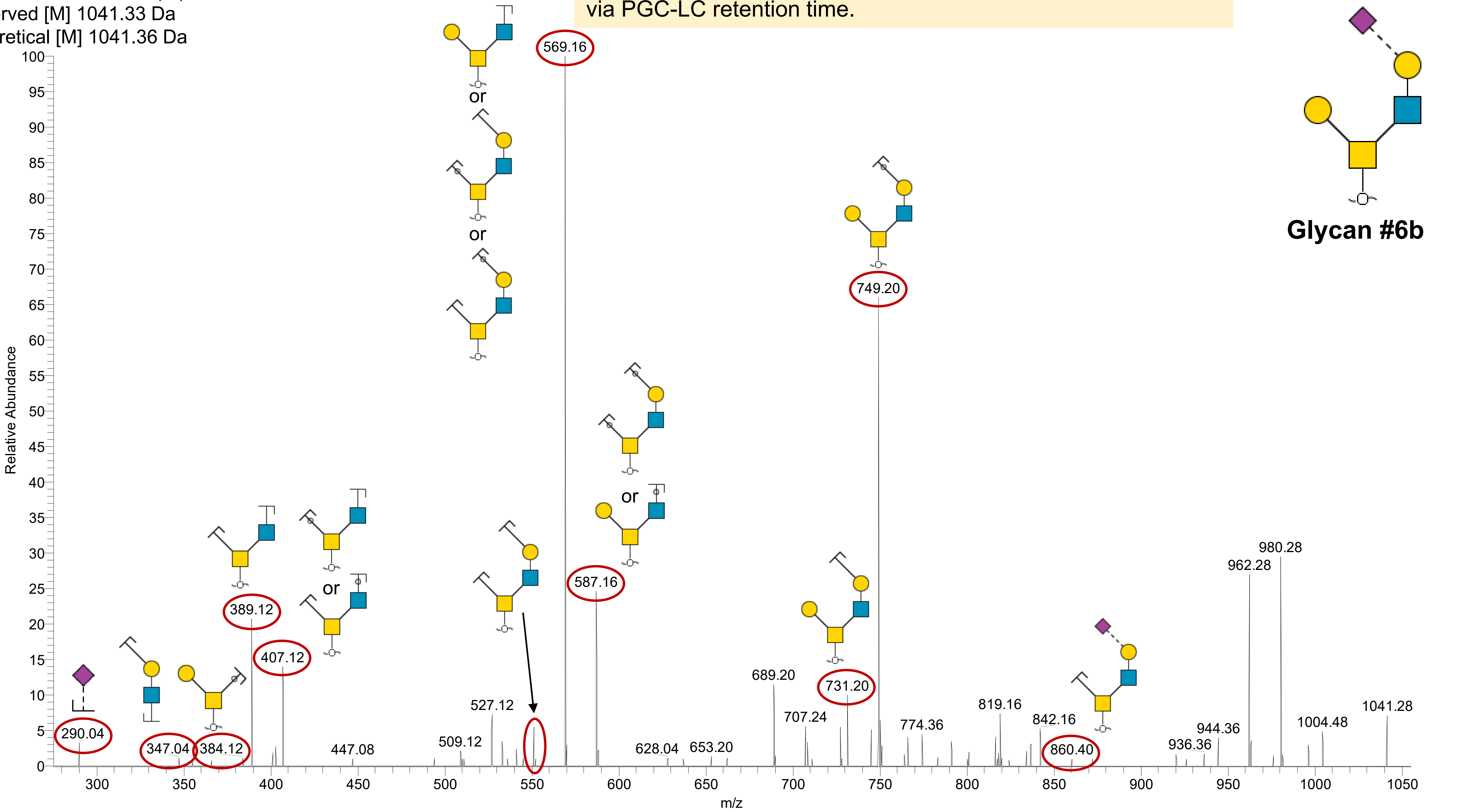

Glycan #7
Observed *m/z* 1186.38 (1-), RT: ~26.06 min
Observed [M] 1187.39 Da
Theoretical [M] 1187.42 Da

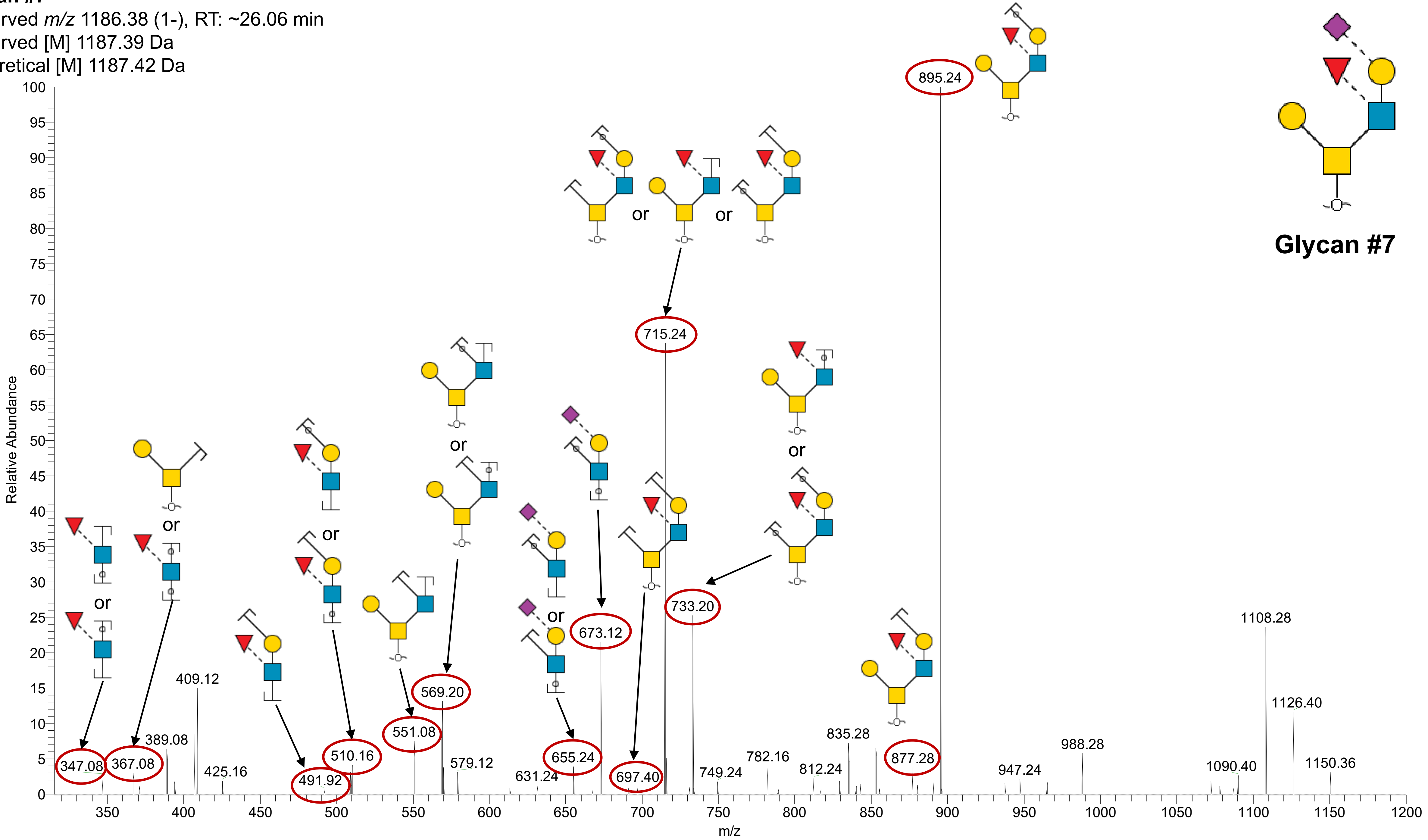

Glycan #8
Observed *m/z* 1257.40 (1-), RT: ~24.17 min
Observed [M] 1258.41 Da
Theoretical [M] 1258.42 Da

Note: This glycan has not been annotated with sialyl linkages due to not enough supporting evidence from the PGC-LC elution pattern.

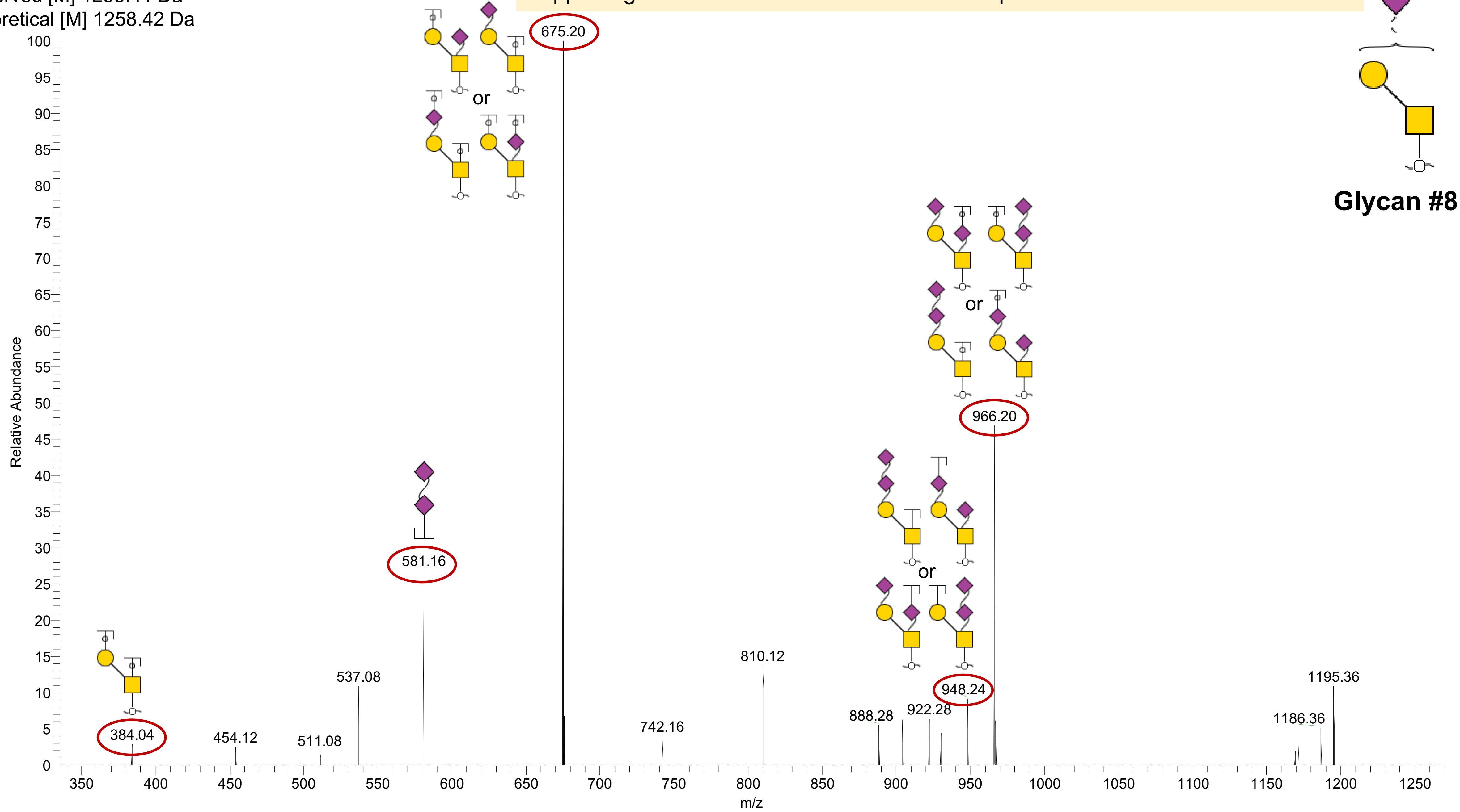

Glycan #9
Observed *m/z* 1331.44 (1-), RT: ~33.50 min
Observed [M] 1332.45 Da
Theoretical [M] 1332.46 Da

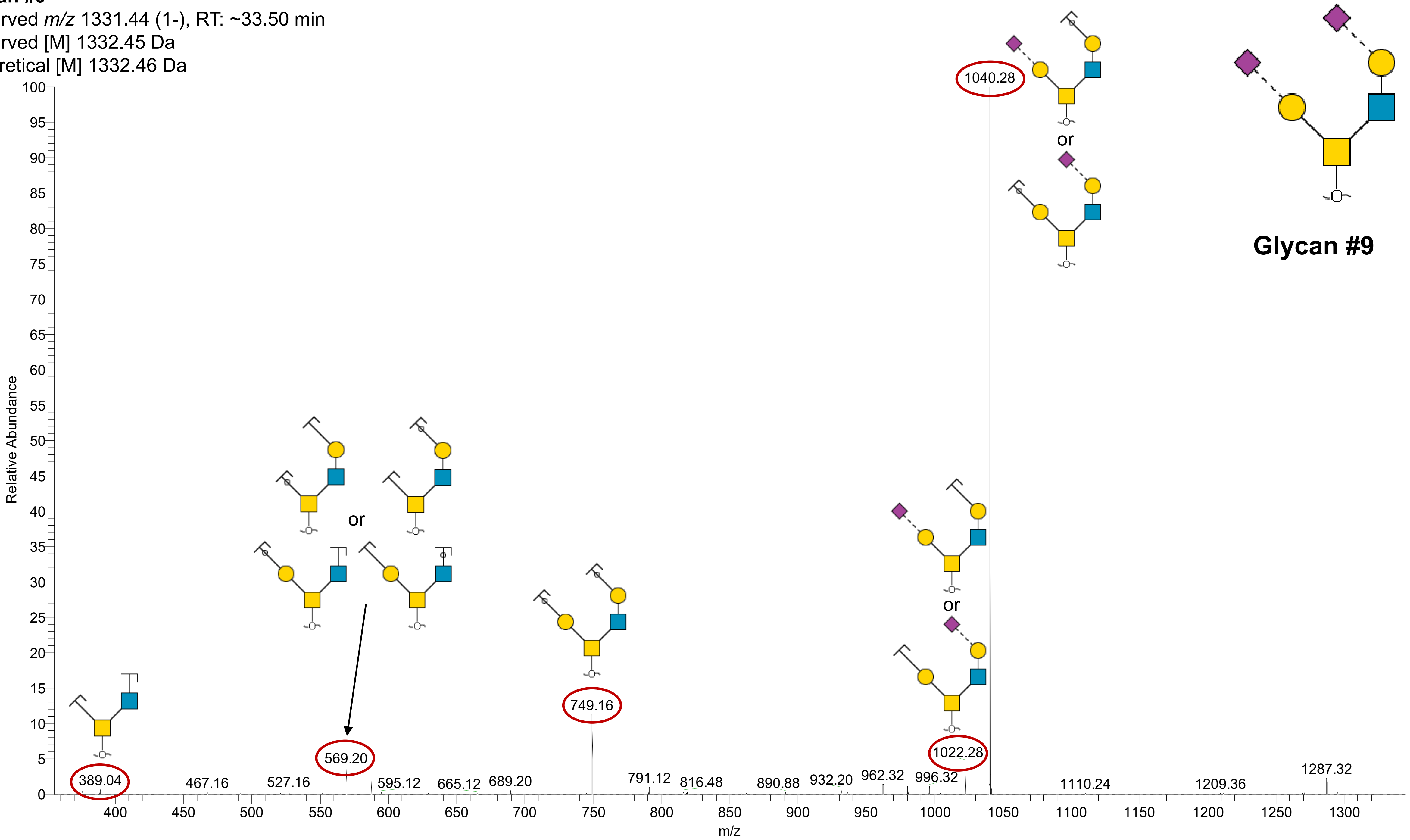

Supplement: Supplemental File 3 — PGC-LC-MS/MS-based O-Glycan characterisation including annotated spectra and peak quantification. [file mmc11.pdf]
