# Supplementary material for: Analysis of the Healthy Platelet Proteome Identifies a New Form of Domain-Specific O-Fucosylation
Source: Mol Cell Proteomics. 2024 Jan 16;23(2):100717. doi: 10.1016/j.mcpro.2024.100717 (PMC10879016; doi:10.1016/j.mcpro.2024.100717)
Supplement: Supplemental File 4 — Annotated EThcD fragmentation MS/MS spectra for identified O-glycosylated peptides. [file mmc12.pdf]

Supplementary File 4

Annotated EThcD fragmentation MS/MS spectra for identified *O*-glycosylated peptides.

Fragmentation Key:

Each MS/MS spectrum is annotated in the top left corner in the following format:
(GENE)_(Modified residue)_(Glycosylation modification)

Ions in the MS/MS spectrum are annotated as either:

- c-ions
- z.-ions
- y-ions
- b-ions
- a-ions

- ~y-ions with a neutral loss (e.g. whole glycan loss)
- ~b-ions with a neutral loss (e.g. whole glycan loss)
- ~a-ions with a neutral loss (e.g. whole glycan loss)

- M (intact precursor)
- Pep_2+ (intact precursor that has undergone a neutral loss)
- HexNAc (oxonium and immonium ions)

- Mass error for fragment matching is <20 ppm

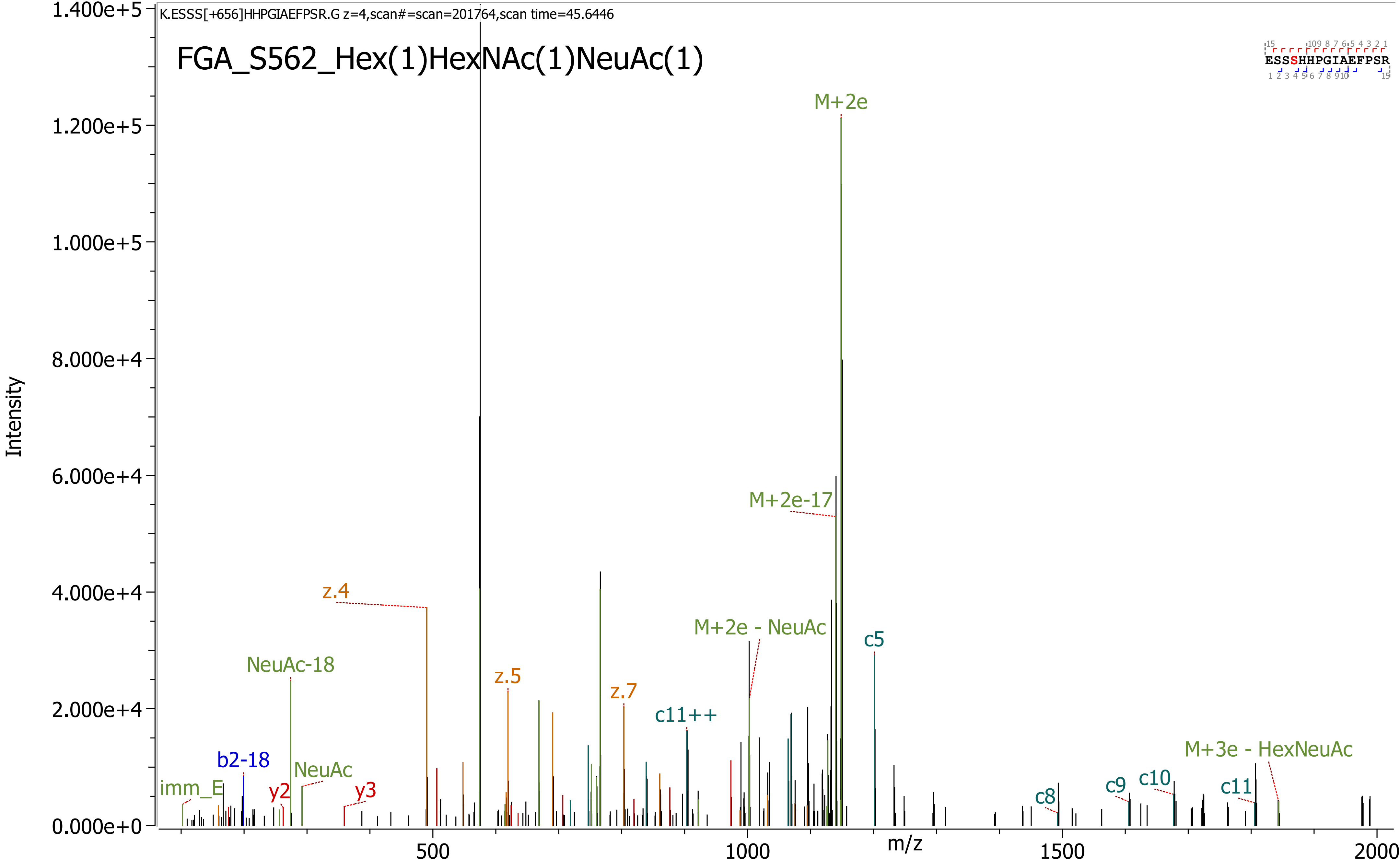
K.ESSS[+656]HHPGIAEFPSR.G z=4,scan#=scan=201764,scan time=45.6446
FGA_S562_Hex(1)HexNAc(1)NeuAc(1)
ESSSHHPGIAEFPSR
Intensity
m/z
M+2e
M+2e-17
M+2e - NeuAc
c5
z.4
z.5
z.7
c11++
NeuAc-18
NeuAc
b2-18
imm_E
y2
y3
c8
c9
c10
c11
M+3e - HexNeuAc

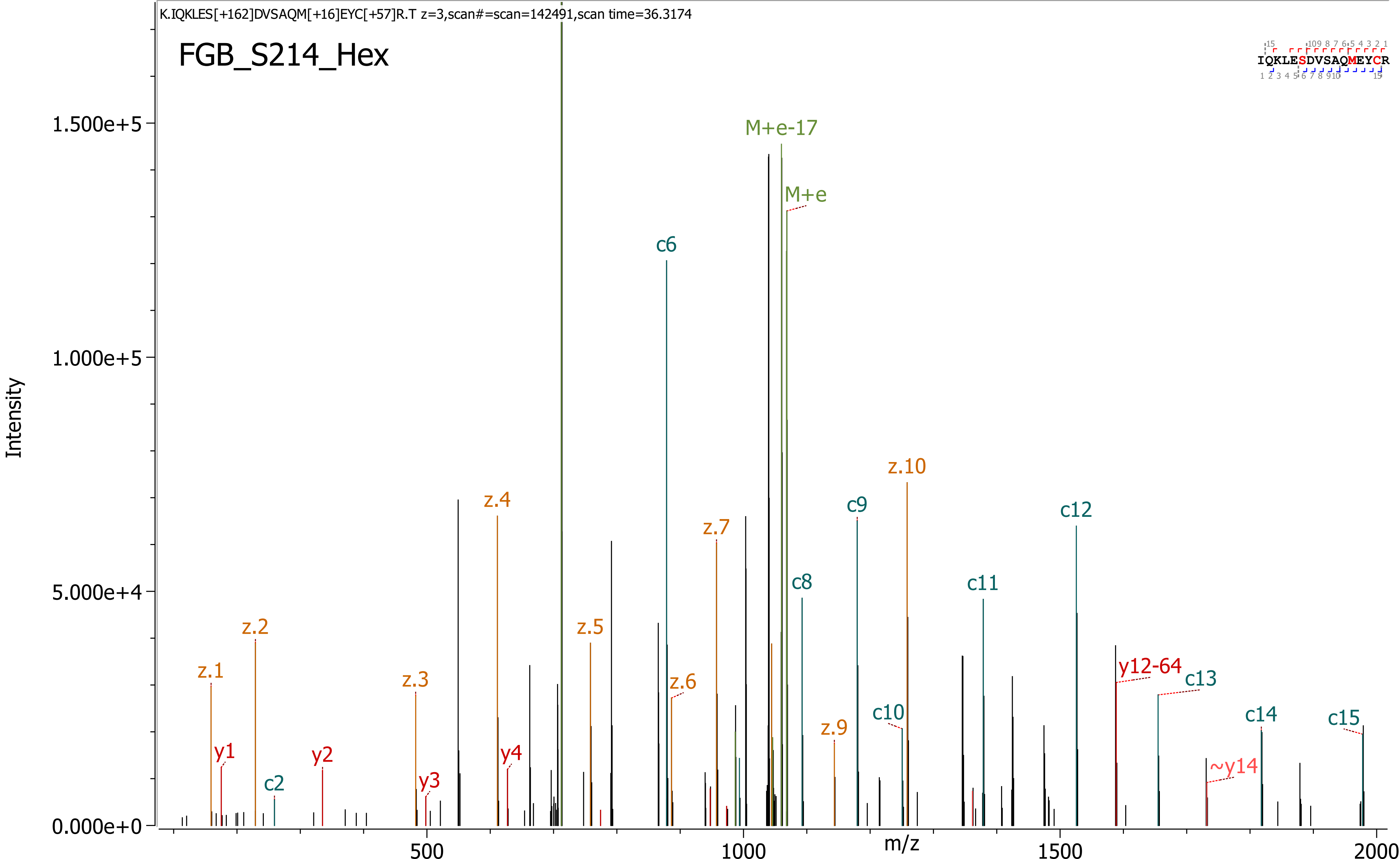
K.IQKLES[+162]DVSAQM[+16]EYC[+57]R.T z=3,scan#=scan=142491,scan time=36.3174
FGB_S214_Hex
IQKLESDVSAQMEYCR
Intensity
m/z
M+e-17
M+e
c6
z.10
c9
z.4
z.7
c12
c8
c11
z.5
z.2
z.1
z.3
z.6
y12-64
c13
y1
y2
y4
y3
c2
z.9
c10
c14
c15
~y14

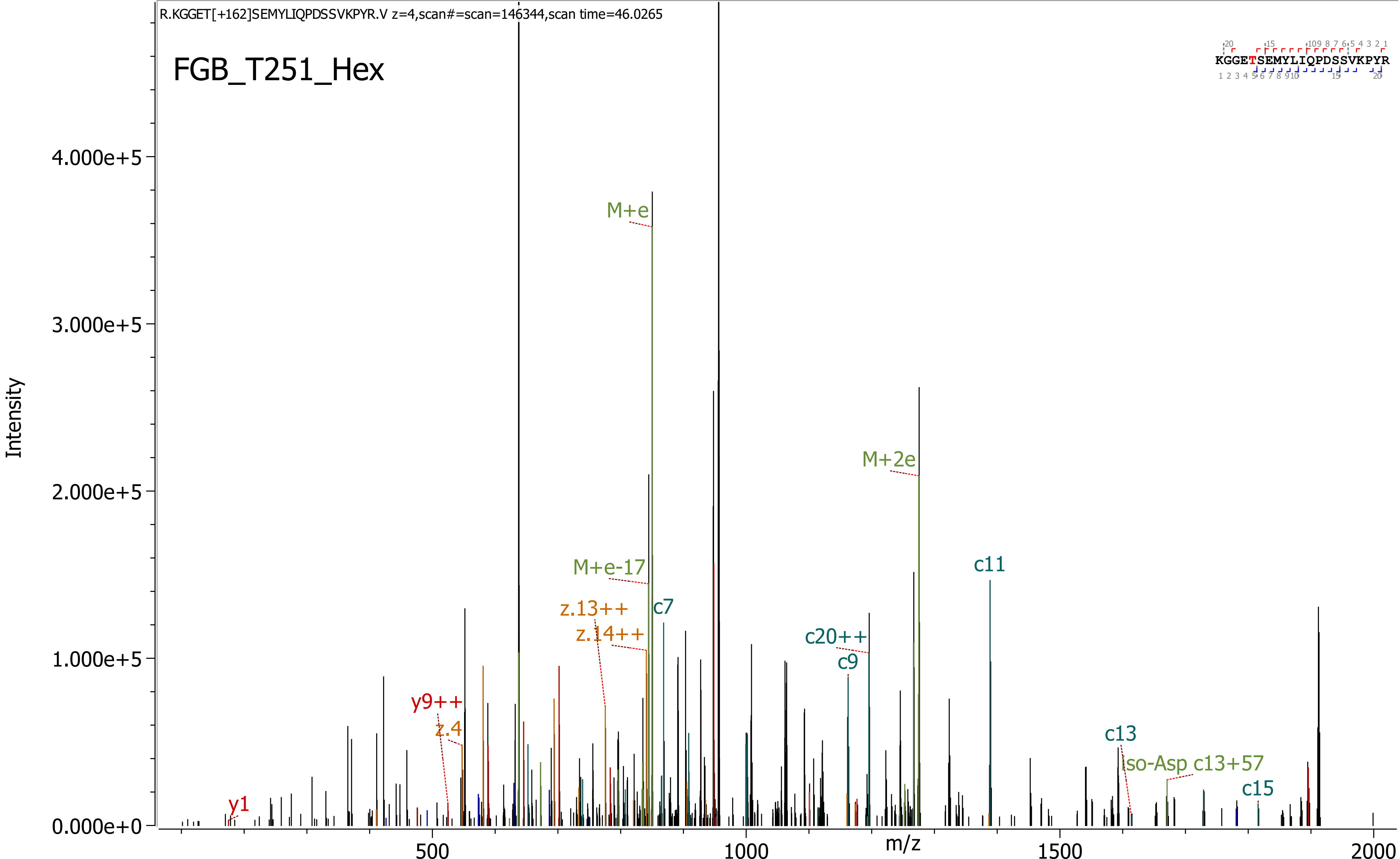
R.KGGET[+162]SEMYLIQPDSSVKPYR.V z=4,scan#=scan=146344,scan time=46.0265
FGB_T251_Hex
KGGETSEMYLIQPDSSVKPYR
Intensity
m/z
4.000e+5
3.000e+5
2.000e+5
1.000e+5
0.000e+0
500
1000
1500
2000
M+e
M+2e
M+e-17
c7
c11
z.13++
z.14++
c20++
c9
y9++
z.4
c13
Iso-Asp c13+57
c15
y1

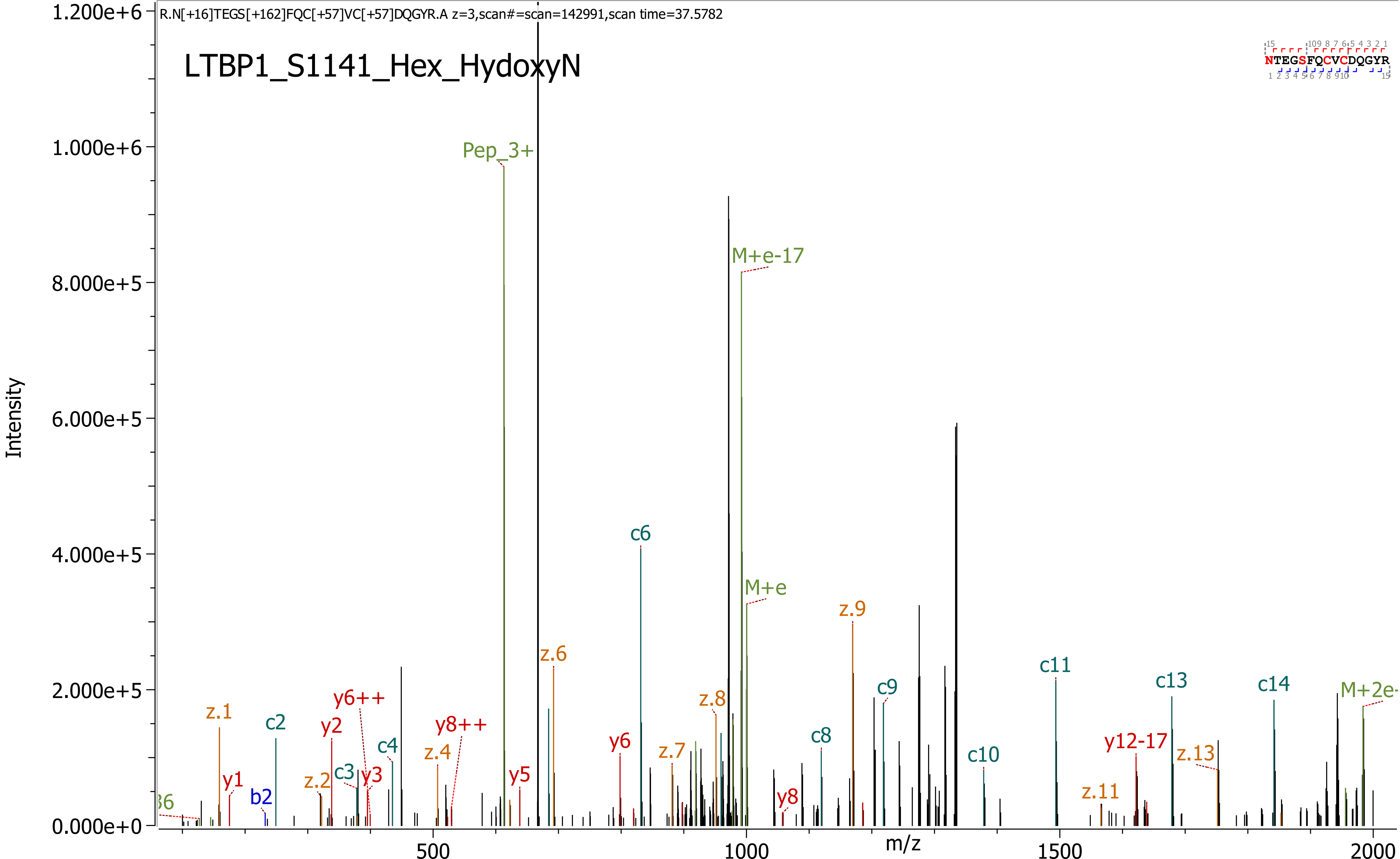
R.N[+16]TEGS[+162]FQC[+57]VC[+57]DQGYR.A z=3,scan#=scan=142991,scan time=37.5782
LTBP1_S1141_Hex_HydoxyN
NTEGSFQCVCDQGYR
Intensity
m/z
1.200e+6
1.000e+6
8.000e+5
6.000e+5
4.000e+5
2.000e+5
0.000e+0
500
1000
1500
2000
Pep_3+
M+e-17
M+e
c6
z.9
z.6
c11
c13
c14
c9
z.8
c8
c10
y12-17
z.13
z.11
y6
z.7
y8
y6++
y8++
z.1
c2
y2
c4
z.4
y1
z.2
c3
y3
y5
b2

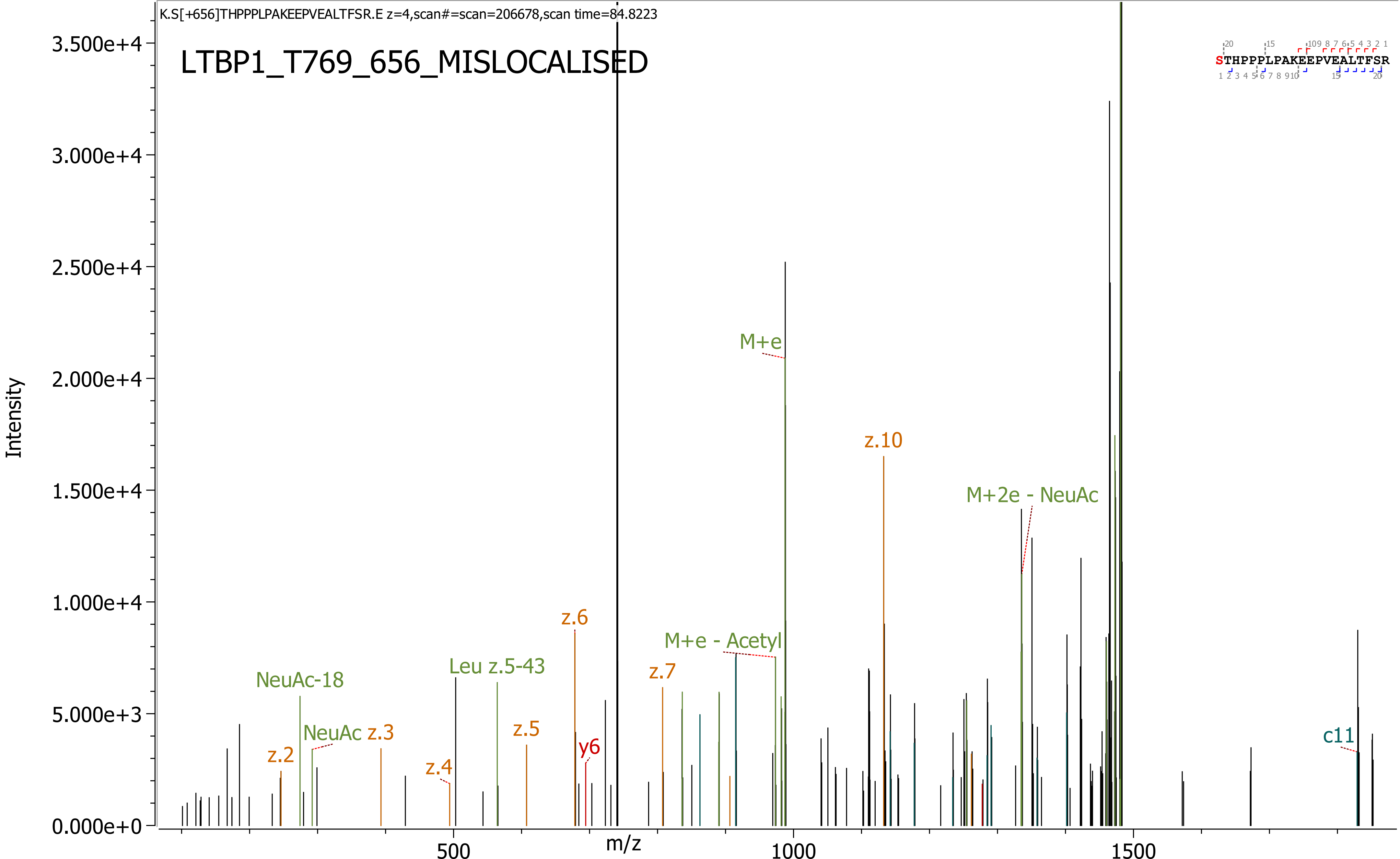
K.S[+656]THPPPLPAKEEPVEALTFSR.E z=4,scan#=scan=206678,scan time=84.8223
LTBP1_T769_656_MISLOCALISED
STHPPPLPAKEEPVEALTFSR
Intensity
m/z
M+e
z.10
M+2e - NeuAc
z.6
M+e - Acetyl
Leu z.5-43
NeuAc-18
z.7
NeuAc z.3
z.5
z.2
y6
z.4
c11
3.500e+4
3.000e+4
2.500e+4
2.000e+4
1.500e+4
1.000e+4
5.000e+3
0.000e+0
500
1000
1500

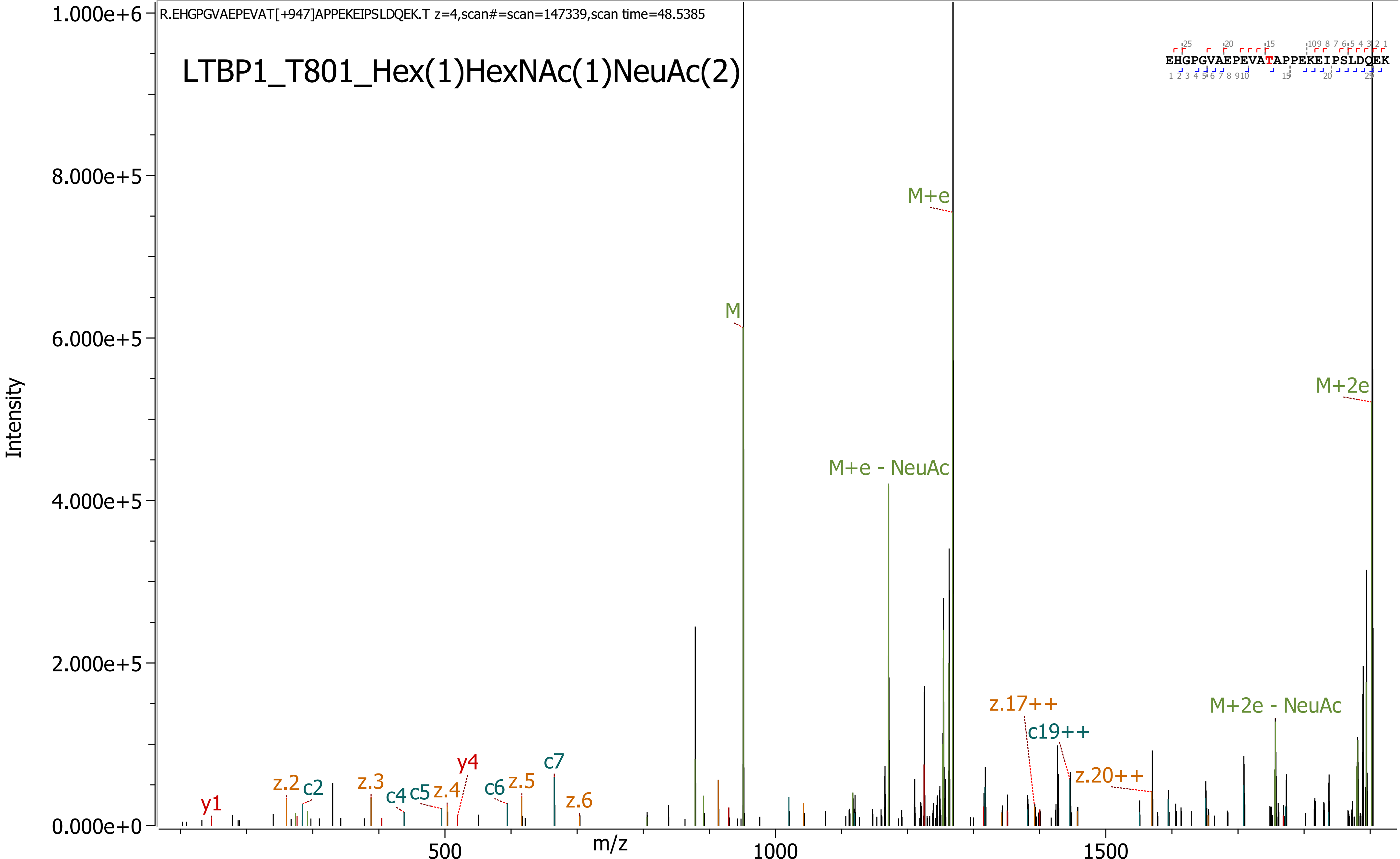

R.EHGPGVAEPEVAT[+947]APPEKEIPSLDQEK.T z=4,scan#=scan=147339,scan time=48.5385
LTBP1_T801_Hex(1)HexNAc(1)NeuAc(2)
EHGPGVAEPEVATAPPEKEIPSLDQEK
Intensity
m/z
1.000e+6
8.000e+5
6.000e+5
4.000e+5
2.000e+5
0.000e+0
500
1000
1500
M
M+e
M+2e
M+e - NeuAc
M+2e - NeuAc
z.17++
c19++
z.20++
y1
z.2
c2
z.3
c4
c5
z.4
y4
c6
z.5
c7
z.6

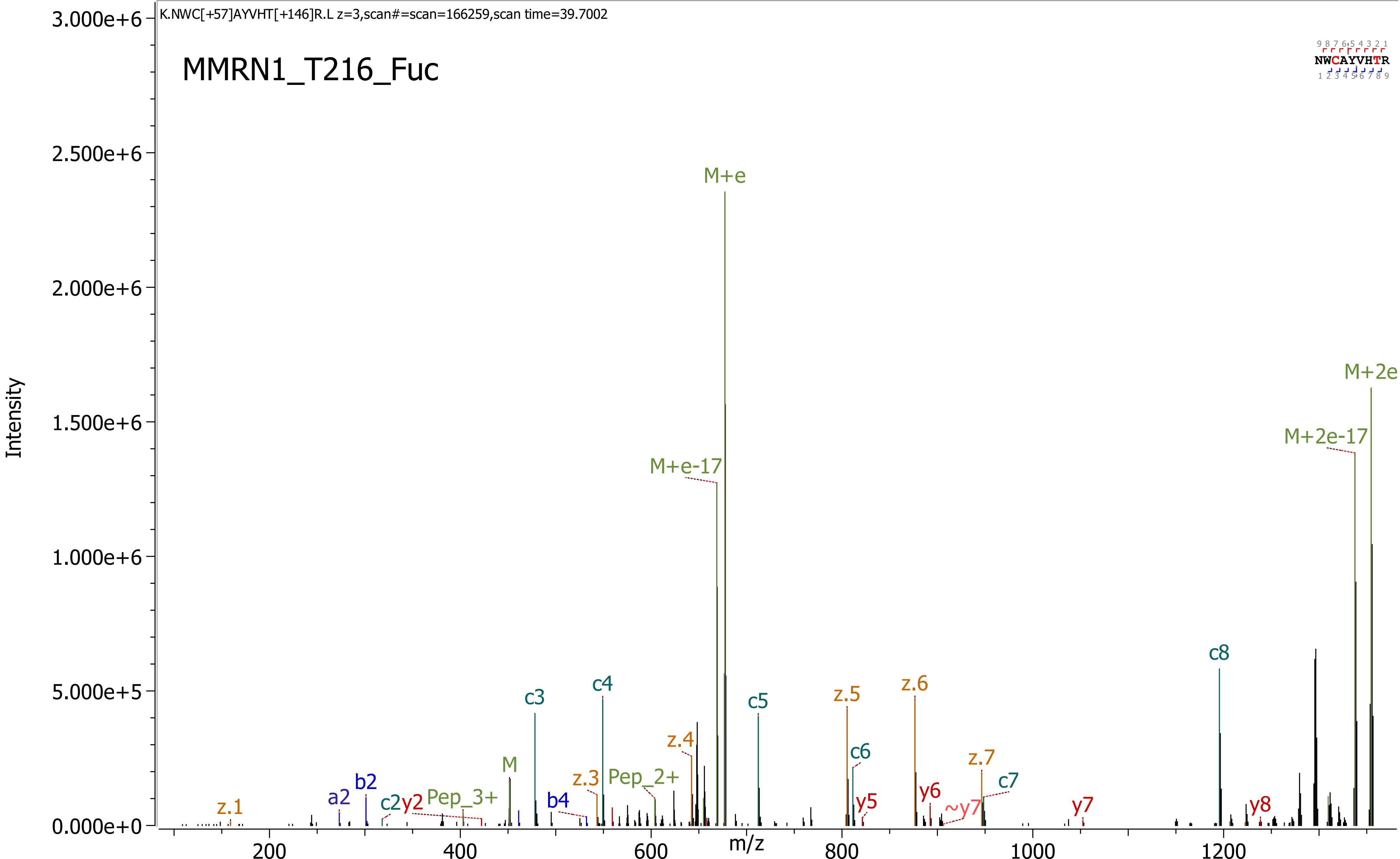
K.NWC[+57]AYVHT[+146]R.L z=3,scan#=scan=166259,scan time=39.7002
MMRN1_T216_Fuc
NWCAYVHTR
Intensity
m/z
M+e
M+2e
M+2e-17
M+e-17
c8
z.6
z.5
c5
c4
c3
c6
z.4
z.7
c7
M
z.3
Pep_2+
b2
a2
b4
c2
y2
Pep_3+
z.1
y5
y6
~y7
y7
y8

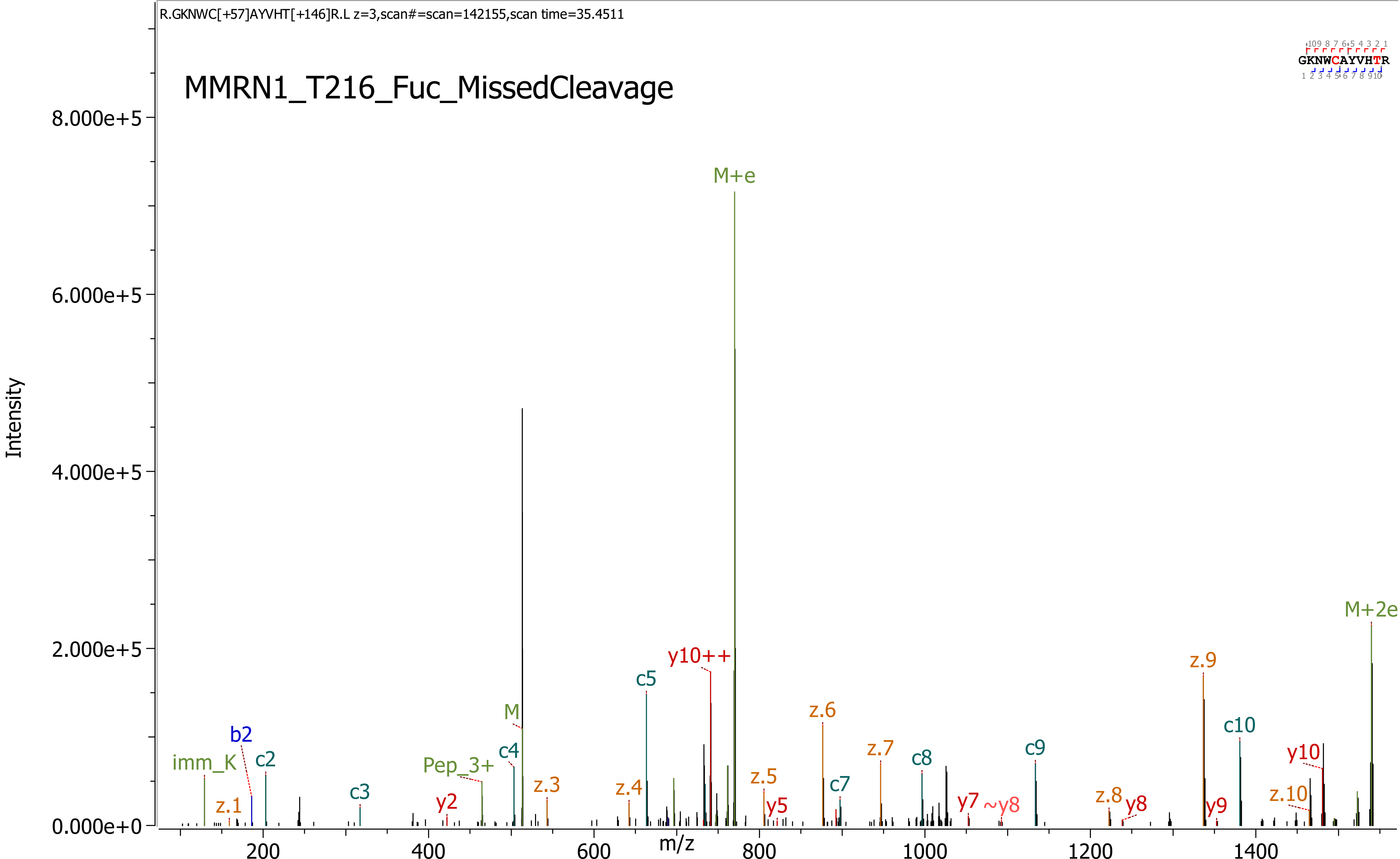
R.GKNWC[+57]AYVHT[+146]R.L z=3,scan#=scan=142155,scan time=35.4511
MMRN1_T216_Fuc_MissedCleavage
GKNWCAYVHTR
Intensity
m/z
8.000e+5
6.000e+5
4.000e+5
2.000e+5
0.000e+0
200
400
600
800
1000
1200
1400
M+e
M+2e
M
Pep_3+
imm_K
b2
c2
c3
c4
c5
c7
c8
c9
c10
z.1
z.3
z.4
z.5
z.6
z.7
z.8
z.9
z.10
y2
y5
y7
~y8
y8
y9
y10
y10++

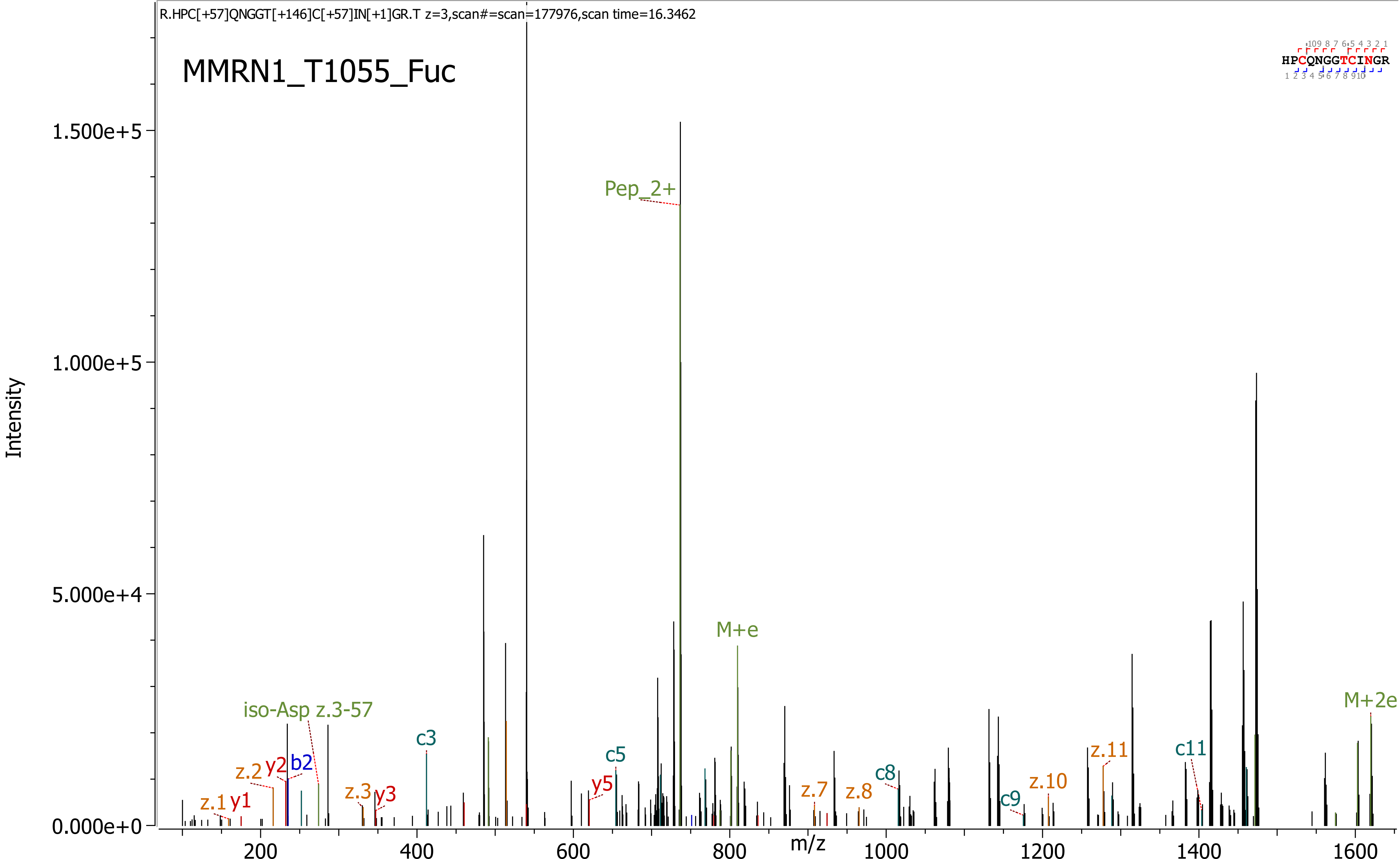
R.HPC[+57]QNGGT[+146]C[+57]IN[+1]GR.T z=3,scan#=scan=177976,scan time=16.3462
MMRN1_T1055_Fuc
HPCQNGGTCINGR
Intensity
m/z
1.500e+5
1.000e+5
5.000e+4
0.000e+0
200
400
600
800
1000
1200
1400
1600
Pep_2+
M+e
M+2e
iso-Asp z.3-57
z.1
y1
z.2
y2
b2
z.3
y3
c3
y5
c5
z.7
z.8
c8
c9
z.10
z.11
c11

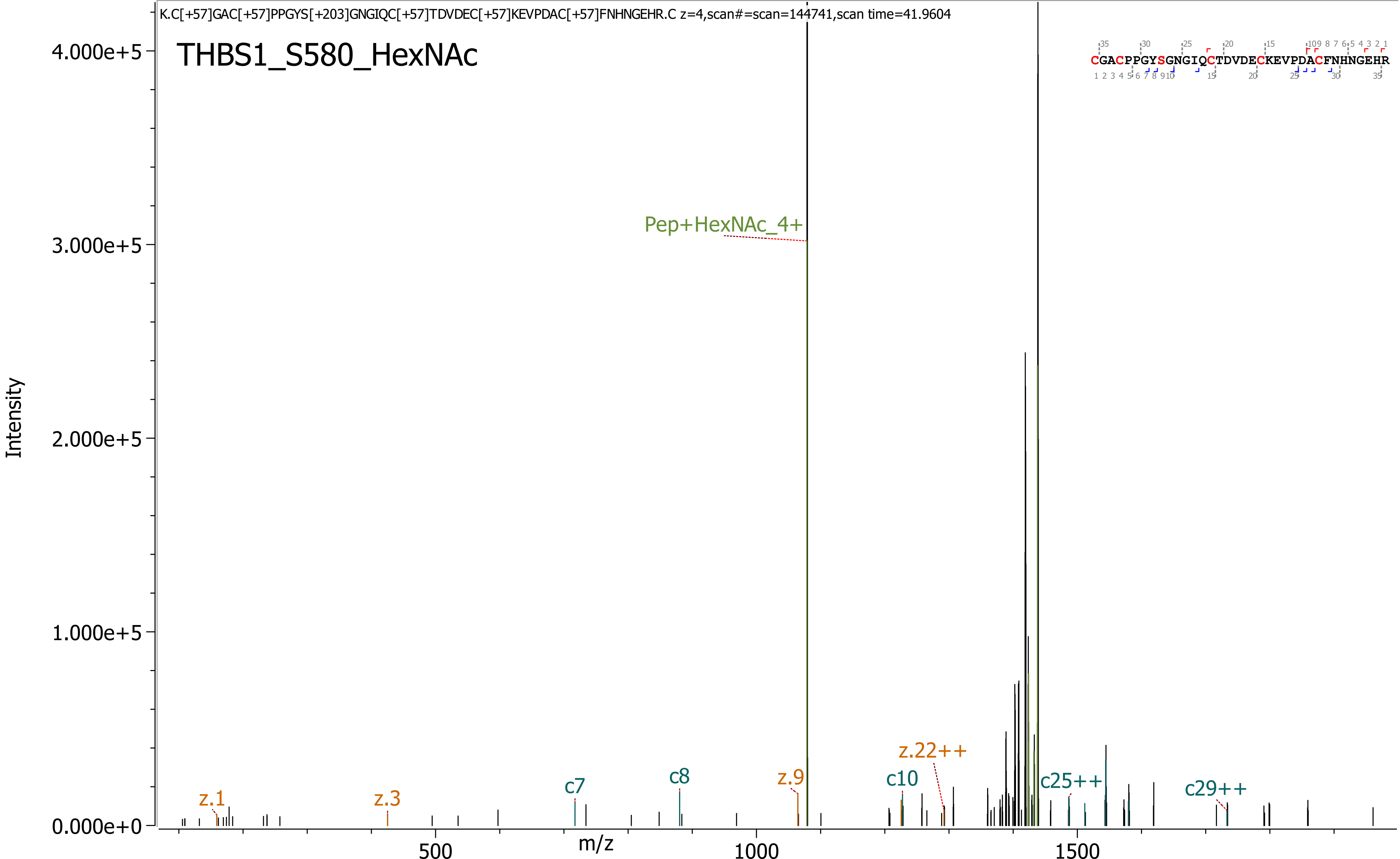
K.C[+57]GAC[+57]PPGYS[+203]GNGIQC[+57]TDVDEC[+57]KEVPDAC[+57]FNHNGEHR.C z=4,scan#=scan=144741,scan time=41.9604
THBS1_S580_HexNAc
CGACPPGYSGNGIQCTDVDECKEVPDACFNHNGEHR
Intensity
4.000e+5
3.000e+5
2.000e+5
1.000e+5
0.000e+0
Pep+HexNAc_4+
z.1
z.3
c7
c8
z.9
c10
z.22++
c25++
c29++
500
1000
1500
m/z

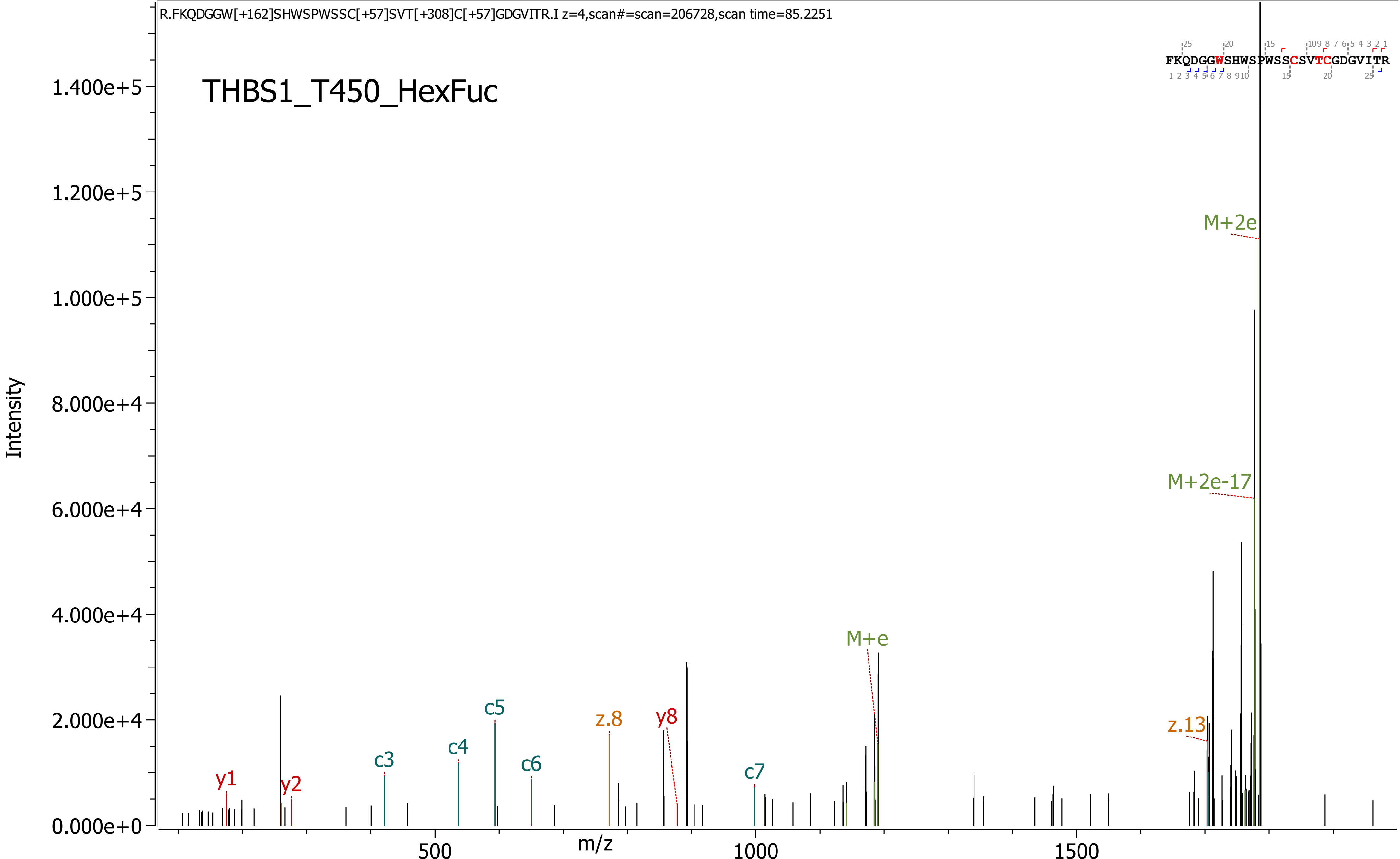
R.FKQDGGW[+162]SHWSPWSSC[+57]SVT[+308]C[+57]GDGVITR.I z=4,scan#=scan=206728,scan time=85.2251
THBS1_T450_HexFuc
FKQDGGWSHWSPWSSCSVTCGDGVITR
Intensity
m/z
M+2e
M+2e-17
M+e
z.13
z.8
y8
c7
c5
c4
c3
c6
y1
y2
0.000e+0
2.000e+4
4.000e+4
6.000e+4
8.000e+4
1.000e+5
1.200e+5
1.400e+5
500
1000
1500

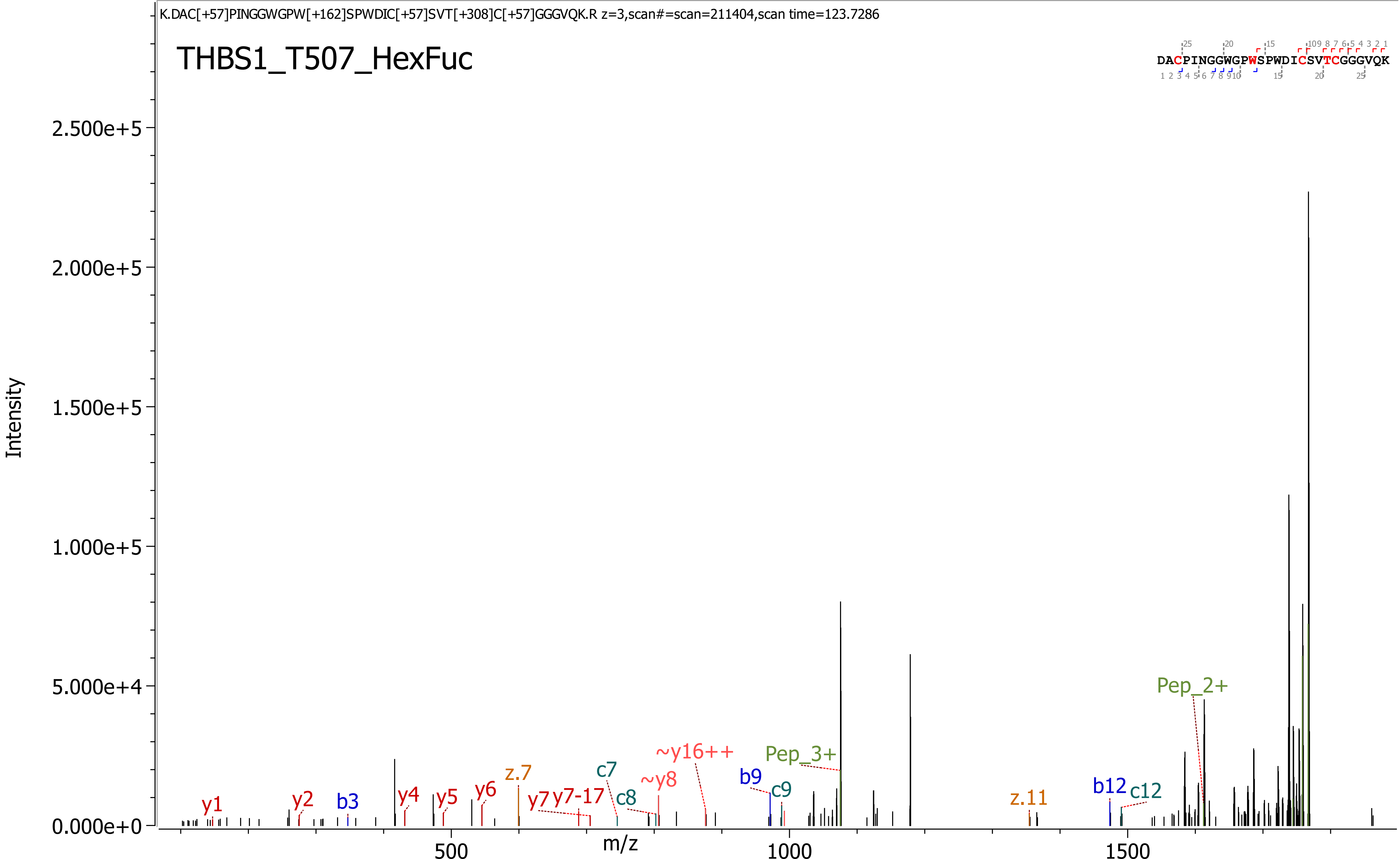
K.DAC[+57]PINGGWGPW[+162]SPWDIC[+57]SVT[+308]C[+57]GGGVQK.R z=3,scan#=scan=211404,scan time=123.7286
THBS1_T507_HexFuc
DACPINGGWGPWSPWDICSVTCGGGVQK
Intensity
m/z
2.500e+5
2.000e+5
1.500e+5
1.000e+5
5.000e+4
0.000e+0
500
1000
1500
y1
y2
b3
y4
y5
y6
z.7
y7
y7-17
c7
c8
~y8
~y16++
b9
c9
Pep_3+
z.11
b12
c12
Pep_2+

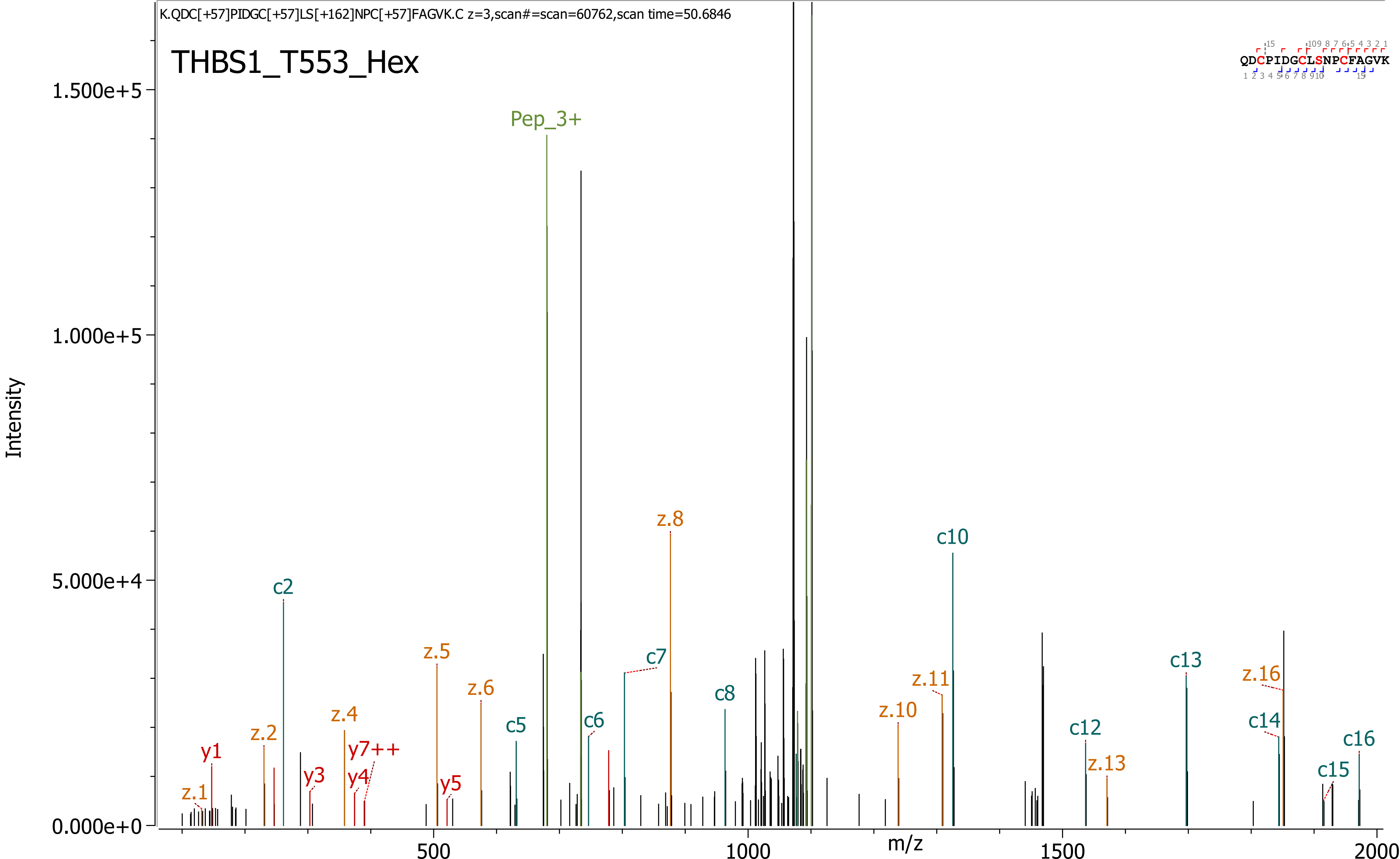
K.QDC[+57]PIDGC[+57]LS[+162]NPC[+57]FAGVK.C z=3,scan#=scan=60762,scan time=50.6846
THBS1_T553_Hex
QDCPIDGCLSNPCFAGVK
Pep_3+
z.1
y1
z.2
c2
y3
z.4
y4
y7++
z.5
y5
z.6
c5
c6
c7
z.8
c8
z.10
z.11
c10
c12
z.13
c13
z.16
c14
c15
c16
Intensity
m/z
0.000e+0
5.000e+4
1.000e+5
1.500e+5
500
1000
1500
2000

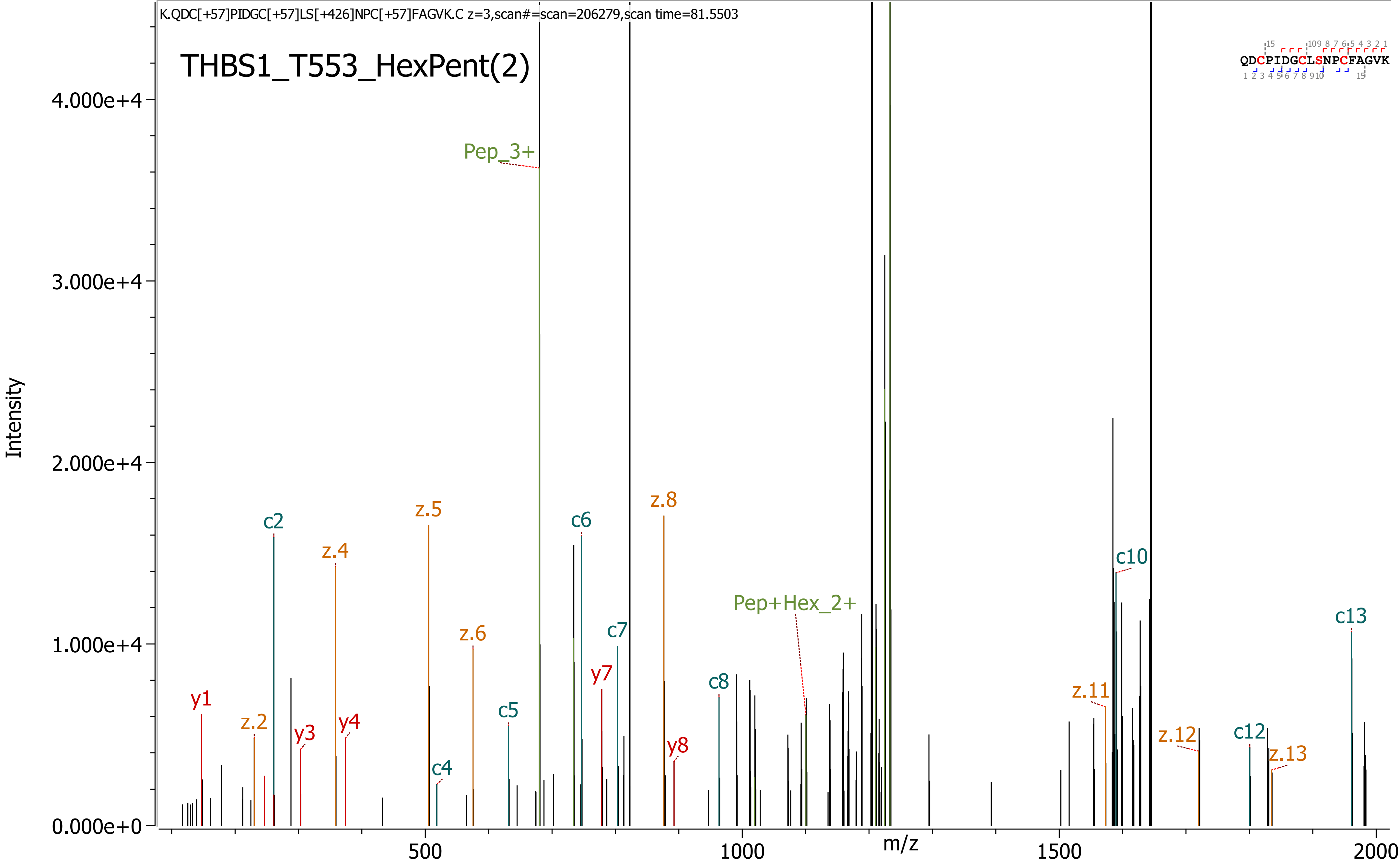
K.QDC[+57]PIDGC[+57]LS[+426]NPC[+57]FAGVK.C z=3,scan#=scan=206279,scan time=81.5503
THBS1_T553_HexPent(2)
QDCPIDGCLSNPCFAGVK
Intensity
m/z
Pep_3+
Pep+Hex_2+
c2
c4
c5
c6
c7
c8
c10
c12
c13
z.2
z.4
z.5
z.6
z.8
z.11
z.12
z.13
y1
y3
y4
y7
y8
0.000e+0
1.000e+4
2.000e+4
3.000e+4
4.000e+4
500
1000
1500
2000
